# An off-target effect of class A CpG-oligonucleotides on suppressing the cyclic GMP-AMP synthase signaling in fibroblastic reticular cells

**DOI:** 10.3389/fphar.2025.1576151

**Published:** 2025-04-23

**Authors:** Preethi Jayakumar, Ting Jiang, Hai Huang, Meihong Deng

**Affiliations:** ^1^ Center for Immunology and Inflammation, The Feinstein Institutes for Medical Research, Manhasset, NY, United States; ^2^ Institute of Molecular Medicine, Feinstein Institutes for Medical Research, New York, NY, United States; ^3^ Tsinghua University School of Medicine, Beijing, China; ^4^ Departments of Molecular Medicine and Surgery, Zucker School of Medicine at Hofstra University/Northwell, Hempstead, NY, United States

**Keywords:** class A ODN, TLR9 agonist, CGAS, ZNF598, fibroblastic reticular cells

## Abstract

**Background:**

Class A CpG-oligonucleotides (ODNs), a Toll-like receptor 9 (TLR9) agonist, have been applied for treating inflammatory diseases and cancer in preclinical studies and clinical trials. A recent study has reported that class A ODNs can activate the Cyclic GMP-AMP synthase (cGAS) signaling to regulate the inflammatory response in human monocytes. However, it remains unknown whether class A ODNs can activate the cGAS pathways in other cell types, such as fibroblastic reticular cells (FRC), which play critical roles in modulating the immune environments during inflammatory diseases and cancer.

**Methods:**

To understand the role of class A ODN in regulating the cGAS signaling in FRC, we treated mouse FRC and human fibroblast with class A ODN, a cGAS agonist (HT-DNA), and combined class A and HT-DNA.

**Results:**

Unexpectedly, we found that class A ODNs suppress the cGAS level and downstream signaling in human and murine FRC. The class A ODN-induced suppression effect on cGAS is limited in FRC, but not other immune cell types, and is independent of TLR9. Performing pulldown assay and Mass spectrum, we found that class A ODNs regulate the cGAS level post translationally by interacting with cGAS and ZNF598, an E3 ubiquitin ligase.

**Conclusion:**

Our data reveal an unrecognized off-target effect of class A ODN on suppressing the cGAS signaling in FRCs, which should be considered when designing class A ODN regimens for inflammatory diseases and cancer.

## Introduction

CpG oligodeoxynucleotides (ODNs) are short, single-stranded synthetic DNA made up of unmethylated cytosine-guanine (CpG) motifs, which mimic bacterial DNA to activate the TLR9 signaling (Bode et al., 2011). TLR9 is an endosomal receptor sensing endogenous and exogenous single-stranded (SS) unmethylated CpG DNA to initiate immune responses (Wagner, 2004). There are 4 classes of ODN (class A, B, C, P) based on structural characteristics and activity in human peripheral blood mononuclear cells, mainly B cells and plasmacytoid dendritic cells (Bode et al., 2011). Preclinical studies have demonstrated that CpG ODNs can act as a vaccine adjuvant for infections and cancer (Krieg, 2006; Bode et al., 2011; Luchner et al., 2021; Zhang et al., 2021). As the TLR9 signaling is critical in controlling immune responses during health and diseases (Wagner, 2004), CpG ODNs are attractive pharmacological therapeutics targeting the TLR9 signaling to control inflammatory responses in diseases.

Fibroblastic reticular cells (FRC) are a subpopulation of stromal cells in lymphoid and non-lymphoid tissues (Alvarenga and Marti, 2014; Fletcher et al., 2015). As stromal cells, FRC are known to provide scaffold support for organ and tissue formation. Emerging evidence has demonstrated that FRC modulates the immune environment in organs and tissues during health and diseases (Fletcher et al., 2015; Fletcher et al., 2020; Acton et al., 2021). FRC regulate tumorigenesis and metastasis via modulating the immune microenvironment (Jiang et al., 2022; Lin and Gordon, 2023; Onder et al., 2024). These make FRC an attractive therapeutic target for inflammatory diseases and cancer. However, molecular mechanisms involved in controlling the immunoregulatory functions of FRC (Thompson, 1966) are not fully understood. We have shown that activation of TLR9 with class A, B, or C ODN can suppress the cytokine and chemokine production in FRC, of which class A ODN shows the most potent suppressive effect (Xu et al., 2019). These make class A ODN an attractive candidate for targeting the TLR9 signaling in FRC to control inflammatory responses during inflammation and cancers.

However, a recent study has demonstrated that cytosolic class A ODN can bind to Cyclic GMP-AMP synthase (cGAS) and activate its downstream signaling to initiate inflammatory responses in human monocytes (Bode et al., 2021). The cGAS-Stimulator of Interferon Genes (STING) is another crucial DNA sensing signaling pathway controlling pathophysiology in tissues and organs (Decout et al., 2021; Samson and Ablasser, 2022). Upon engaging with double-stranded DNA in the cytosol, cGAS produces Cyclic GMP-AMP (cGAMP), which acts as a second messenger and subsequently triggers STING and its downstream transcriptional factors IRF3 or NFκB to initiate inflammatory responses (Decout et al., 2021). The cGAS-STING signaling is implicated in the inflammatory response and senescence in fibroblasts during tumorigenesis (Yang et al., 2017; Hou et al., 2021; Liu et al., 2024). However, whether class A ODN can activate the cGAS signaling in FRC remains unknown.

Unexpectedly, we found that the treatment of class A ODN, but not class B or C ODNs, negatively regulates the cGAS level and its downstream signaling in FRC. The negative regulatory effect of class A ODN on cGAS signaling in FRC was independent of TLR9. Class A ODN regulated the cGAS level post translationally by interacting with cGAS and ZNF598, an E3 ubiquitin ligase. Our results reveal an unrecognized off-target effect of class A ODN on downregulating the cGAS levels in FRC. Considering the immunoregulatory role of FRC and the preclinical and clinical applications of class A ODN in inflammatory diseases and cancer in preclinical studies and clinical trials (Krieg, 2006; Kayraklioglu et al., 2021; Luchner et al., 2021; Zhang et al., 2021; Dongye et al., 2022), these data will provide critical information for designing class A ODN-based therapies to treat inflammatory diseases and cancer.

## Materials and methods

### Reagent

ODN1585 (Invivogen # tlrl-1585), ODN2336 (Invivogen # tlrl-2336), ODN2216 (Invivogen # tlrl-2216), ODN1826 (Invivogen # tlrl-1826), ODN2395 (Invivogen # tlrl-2395), ODN1585-biotin (Alpha diagnostics #ODN1585-B), LPS-EB biotin (Invivogen # tlrl-lpsbiot), HT-DNA (DNA from herring testes Sigma Aldrich #D6898), Cycloheximide (Sigma Aldrich #C4859), MG-132 (Invivogen #tlrl-mg132), Morpholinos (Genetools, Vivo-morpholino-znf598).

### Mice

WT C57BL/6J mice, were purchased from the Jackson laboratory. C57BL/6(C)-Cgas^tm1d(EUCOMM)Hmgu/J^ (cGAS^−/−^, stock #026554) (Schoggins et al., 2014) and C57BL/6-*Tlr9* ^*em*1.1*Ldm*^/J (TLR9^−/−^, stock #034449) (Querrey et al., 2022) mice on a C57BL/6 background were purchased from Jackson Laboratory and were bred in our animal facility. All mice were maintained under pathogen-free conditions. Mice were randomly assigned to different experimental or control groups between 6 and 8 weeks of age. Upon arriving, all mice were acclimated in our animal facility for 3 or more days before any experiments.

All animal studies were approved by the Institutional Animal Care and Use Committees of the Animal Care and Use Committee of The Feinstein Institute for Medical Research. Experiments were performed in adherence to the National Institutes of Health Guidelines.

### Isolation and *ex vivo* expansion of mice FRC

Mice were sacrificed, and blood was extracted by cardiac puncture. Omentum was collected. Mesenteric adipose tissue was carefully excised from the small intestine, large intestine, and cecum using scissors without rupture of the intestines. Mesenteric lymph nodes were removed. The pancreas was kept untouched. Omentum and mesentery adipose tissue were minced in RPMI 1640 Medium (Gibco) containing 2% FBS (Biotechne), 60 μg/mL Liberase TL (millpore sigma), 250 μg/mL DNase I (millpore sigma). Minced adipose tissue was placed at 37°C for 30–45 min, shaking. Digestion was stopped by adding 4x volume RPMI 1640 with 10% FBS. Digested tissue was filtered through a 70 μM sterile filter, then centrifuged for 5 min at 250 × g. The supernatant was discarded, and cells were resuspended in MesenCult™ Expansion medium (stemcell). Cells were cultured at 37°C with 5% CO_2_ and used for *in vitro* experiments after 7 days.

### RNA isolation and quantitative real-time PCR (qRT-PCR)

Total RNA was extracted using Qiagen kit (Cat# 74136). The total amount of RNA in each sample was quantified using Take3 plate spectrophotometer (BioTek Synergy LX reader). One microgram of total RNA in each sample was reverse transcribed with iScript cDNA synthesis kit (Biorad Cat# 1708891) per manufacturer’s instructions.

Quantitative PCR was performed using the SSO Advanced SYBR Green Master Mix (Cat# 1725271) with the Biorad PCR detection system. The samples were set up in triplicate in a 10 μL total volume. Specific primers were used as shown in Table 1 below. All primer sequences were further verified using BLAST search. The following two-step PCR conditions were applied: 95°C for 10 min before 40 cycles of 95°C for 15 s, 60°C for 30 s as per manufacturer’s instructions. Samples were compared using the relative Ct method. The results of real-time RT-PCR were presented as target gene expression (fold induction), which were calculated using gapdh as internal control.

**TABLE 1:** Primer list for real-time PCR.

Name	Sequence/description	
Ms_cGAS	Forward Primer	GAG​GCG​CGG​AAA​GTC​GTA​A
	Reverse Primer	TTG​TCC​GGT​TCC​TTC​CTG​GA
Ms_CXCL10	Forward Primer	CGA​TGA​CGG​GCC​AGT​GAG​AAT​G
	Reverse Primer	TCA​ACA​CGT​GGG​CAG​GAT​AGG​CT
Ms_IL6	Forward Primer	TAG​TCC​TTC​CTA​CCC​CAA​TTT​CC
	Reverse Primer	TTG​GTC​CTT​AGC​CAC​TCC​TTC
Ms_TNFα	Forward Primer	GAT​CGG​TCC​CCA​AAG​GGA​TG
	Reverse Primer	GGT​TTG​CTA​CGA​CGT​GGG​C
Ms_GAPDH	Forward Primer	AAC​TTT​GGC​ATT​GTG​GAA​GG
	Reverse Primer	ACA​CAT​TGG​GGG​TAG​GAA​CA
Ms_IFNβ	Product Name: Mm_IFNb1_1_SG QuantiTect Primer Assay GeneGlobe Id: QT00249662 Catalog Number: 429244378	

### Western blot

After treatments, cells were harvested by briefly washing with PBS and scraping into ice-cold RIPA buffer that contained a mammalian protease and phosphatase inhibitor cocktail (Sigma-Aldrich; Cat#PPC1010). Insoluble material was removed by centrifuging (16,000 ×g) and discarding the pellet. The protein concentration in the supernatant was determined with a Bradford assay (ThermoFisher Scientific; Cat# 23225), and lysates were stored at −80°C. Just before use, proteins were denatured (100°C for 5 min in a dry-bath incubator) in a reducing sample buffer containing loading dye. Samples were loaded at 30 μg protein/lane on 4%–12% Bis-Tris gels (Thermofisher Cat# NW04125BOX) and subjected to SDS-PAGE for 1.5–2 h at 120 mV. Proteins were then transferred to a PVDF membrane and blocked for 5 min in EveryBlot Biorad blocking buffer (Cat# 12010020).

Protein levels were measured for cGAS, p-STING/T-STING, p-IRF3/T-IRF3, p-NF-κB p65, ZNF598, GAPDH, Actin, and Tubulin. Primary antibodies (incubated overnight at 4°C) were diluted in Tris-buffered saline with Tween 20 (TTBS) with 1% non-fat dry milk, as follows: rabbit anti-cGAS (1:1000; Cell signaling Cat# D3080 Mouse; proteintech Cat# 26416-1-AP human), rabbit anti-p-STING (1:1000; Cell signaling Cat# 72971), rabbit anti-T-STING (1:1000; Cell signaling Cat# 50494), rabbit anti-p-IRF3 (1:1000; Cell signaling Cat# 29047), rabbit anti-T-IRF3 (1:1000; Cell signaling Cat# 43023), rabbit anti-P- p-NF-κB p65 (1:1000; Cell signaling Cat# 3033), rabbit anti-ZNF598 (1:1000; Bethyl laboratories Inc. Cat# A305-108A), rabbit anti-αTubulin (1:10000; Cell signaling Cat# 2144), mouse anti-β-Actin (1:10000; Cell signaling Cat#3598R-100) and mouse anti-GAPDH (1:10000; Cell signaling Cat#97166). After washing in TTBS (3 × 10 min), membranes were incubated at room temperature for 2 h in horseradish peroxidase-labeled secondary antibodies (1:4000; goat anti-rabbit IgG: Cell signaling, Cat# 7074S; goat anti-mouse IgG: Biorad, Cat # 0300-0108P) in TTBS with 1% non-fat dry milk. After washing (5 × 10 min), membranes were developed with HRP substrate using Azure 300 chemiluminescent Western blot imager (AZI300-01).

### Biotinylated ODN1585 pulldown assay

For pulldown of endogenous cGAS, 1 × 10^6^ FRCs were lysed in RIPA lysis buffer (#, Cell Signaling Technology). Cell debris was removed by centrifugation. Total cell lysate was incubated with 5 μg 3′-biotinylated ODN1585 or 3′-biotinylated LPS as control for 1 h at 4°C followed by prewashed streptavidin-agarose beads (50% w/v) overnight at 4°C. Bead pellets were washed, boiled in sample loading dye, and run on 4%–12% Bis-Tris gels (Thermofisher Cat# NW04125BOX). Blots were probed with rabbit anti-cGAS (Cell signaling Cat# D3080).

### Immunoprecipitation

Total protein were extracted from 3 × 10^6^ FRCs cells treated with or without ODN1585 for 30 min using 1 × IP buffer (Dynabeads Co-Immunoprecipitation kit from invitrogen Cat# 14321D). Then, the lysates were incubated with anti-cGAS coupled magnetic beads overnight at 4°C as per the manufacturer instructions. The next day, beads were washed, protein was eluted in elution buffer, and the sample was run on 4%–12% Bis-Tris gels (Thermofisher Cat# NW04125BOX). The blots were probed with rabbit anti-Ubiquitin (Cell signaling Cat# 3936) and anti-cGAS (Cell signaling Cat# D3080).

### Mass Spectrometry

The above biotinylated ODN1585 pulldown sample proteins were validated by proteomic analysis using Mass Spectrometry (MS) at the Mass Spectrometry and Proteomics core facility, Ohio State University, Ohio, United States. The resulting files were searched using Mascot Daemon by Matrix Science version 2.2.1 accessed through the Proteome Discoverer (PD) software. The data was further analyzed using Scaffold viewer (Scaffold_5.1.2).

### Cytokine array

Cytokine production was performed using human cytokine antibody arrays (Raybio C-Series C3). According to the manufacturer’s recommendation, cytokine antibody-coated membranes were incubated with blocking solution, followed by incubation with culture supernatants for overnight at 4°C. After extensive washings, membranes were incubated with HRP-streptavidin for 2 h at room temperature. After washing, membranes were developed with detection buffer mixture using Azure 300 chemiluminescent Western blot imager (AZI300-01). Signal intensity was calculated by subtracting the background signal using UN-Scan-IT-gel 7.1 software.

### Statistical analysis

Data were expressed as the mean ± S.D. of a minimum of twice or three independent experiments, and graphs were plotted with prism, version 10.0 (GraphPad Software Inc., San Diego, CA, United States). Significant differences were evaluated by one-way ANOVA followed by Tukey *post hoc* multiple comparison test. p-value <0.05 was considered statistically significant.

## Results

### Class A ODN suppresses cGAS-mediated inflammatory response in FRC

**FIGURE 1:**
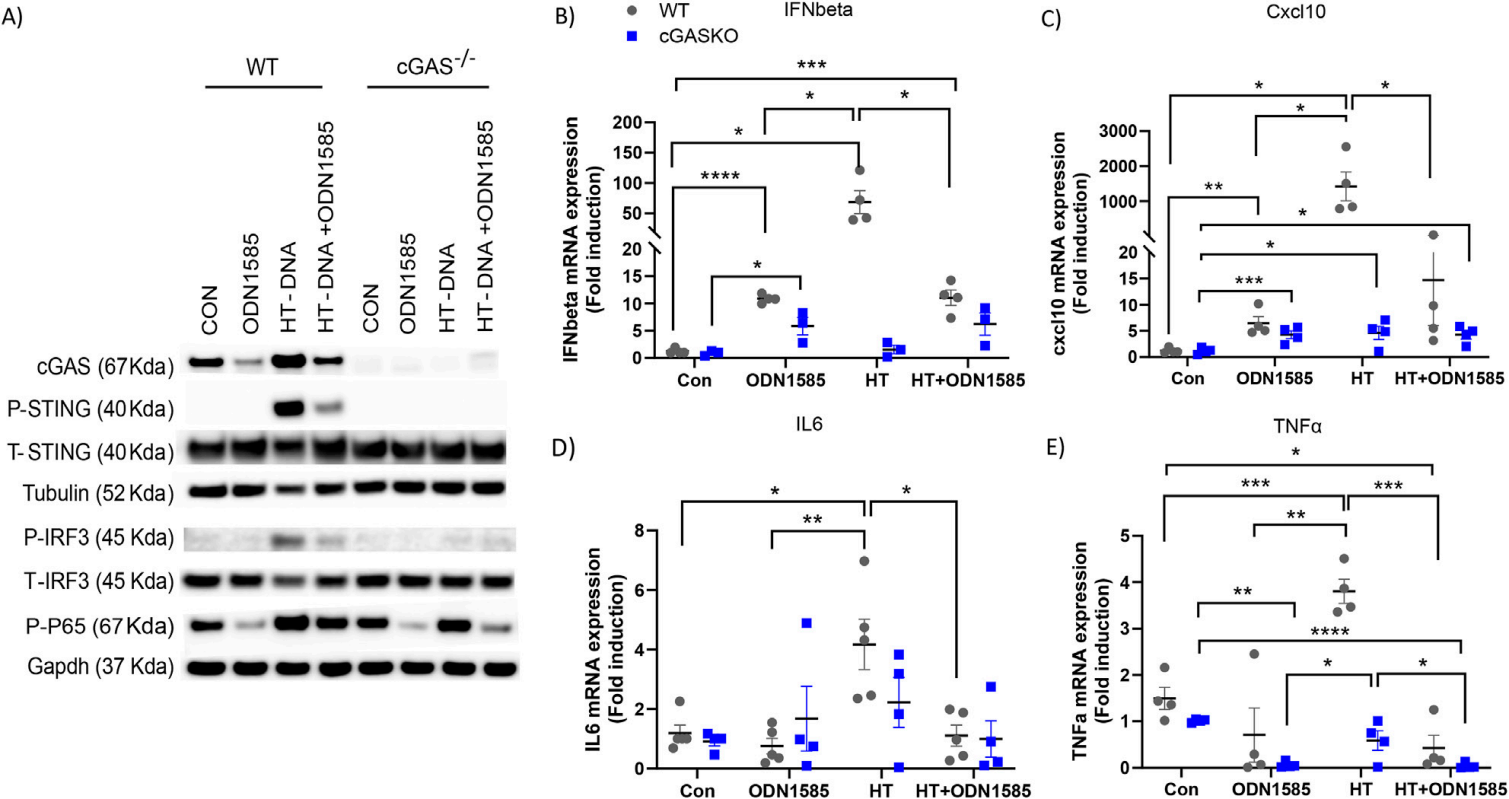
Class A ODN suppresses cGAS-mediated inflammatory response in FRC. FRC were isolated from the mesenteric adipose tissue of WT and cGAS^−/−^ mice and treated with PBS(CON), ODN1585 (5 µM), HT-DNA (1 μg/mL), or combined ODN1585 and HT-DNA for 18 h **(A)** Western blot analysis of cGAS, phosphorylated STING (p-STING), total STING (T-STING), Tubulin (loading control), phosphorylated IRF3 (p-IRF3), total IRF3 (T-IRF3), phosphorylated p65 (p-P65) and GAPDH (loading control) **(B–E)** mRNA levels of Ifnβ **(B)**, Cxcl10 **(C)**, Il6 **(D)**, and Tnfα **(E)** were quantified by qRT-PCR and presented as fold induction relative to untreated control. Experiments have been repeated at least twice. Data are shown as mean ± SEM. Symbols indicate individual values of biological replicates. *p < 0.05, **p < 0.01, ***p < 0.001, ****p < 0.0001.

To study the impact of Class A CpG ODN on the cGAS-mediated response in FRC, we isolated FRC from the mesenteric adipose tissue of WT and cGAS^−/−^ mice and treated them with PBS, ODN1585 (a class A ODN), HT-DNA (Herring testes DNA; a cGAS agonist), or combined ODN1585 and HT-DNA. As expected, treatment of HT-DNA but not ODN1585 activated the downstream signaling of the cGAS-STING signaling pathways in WT-FRC, evidenced by increased levels of cGAS, phospho-STING (p-STING), p-IRF3, and p-P65 (Figure 1A; Supplementary Figures S1A–D). Consistently, treatment of HT-DNA substantially increased the expression levels of cGAS-mediated inflammatory genes in WT FRC, including *Ifnb*, *Cxcl10*, *Il6*, and *Tnfα* (Figures 1B–E). Knockout of cGAS abrogated the HT-DNA effect on activating the cGAS-STING pathway (Figure 1A; Supplementary Figures S1A–D). Surprisingly, treatment with ODN1585 decreased levels of cGAS in WT-FRCs compared with the control (Figure 1A; Supplementary Figures S1A–D). Furthermore, addition of ODN1585 dramatically suppressed HT-DNA-induced activation of cGAS-STING signaling, evidenced by decreased levels of cGAS, p-STING, p-IRF3, and p-P65 (Figure 1A; Supplementary Figures S1A–D) and expression levels of *Ifnb*, *Cxcl10*, *Il6*, and *Tnfα* compared with HT-DNA only treatment (Figures 1B–E). These data indicate that ODN1585 suppresses the cGAS-STING signaling pathway in FRC.

### Class A ODNs downregulate the cGAS level in FRC in a TLR9-independent manner

**FIGURE 2:**
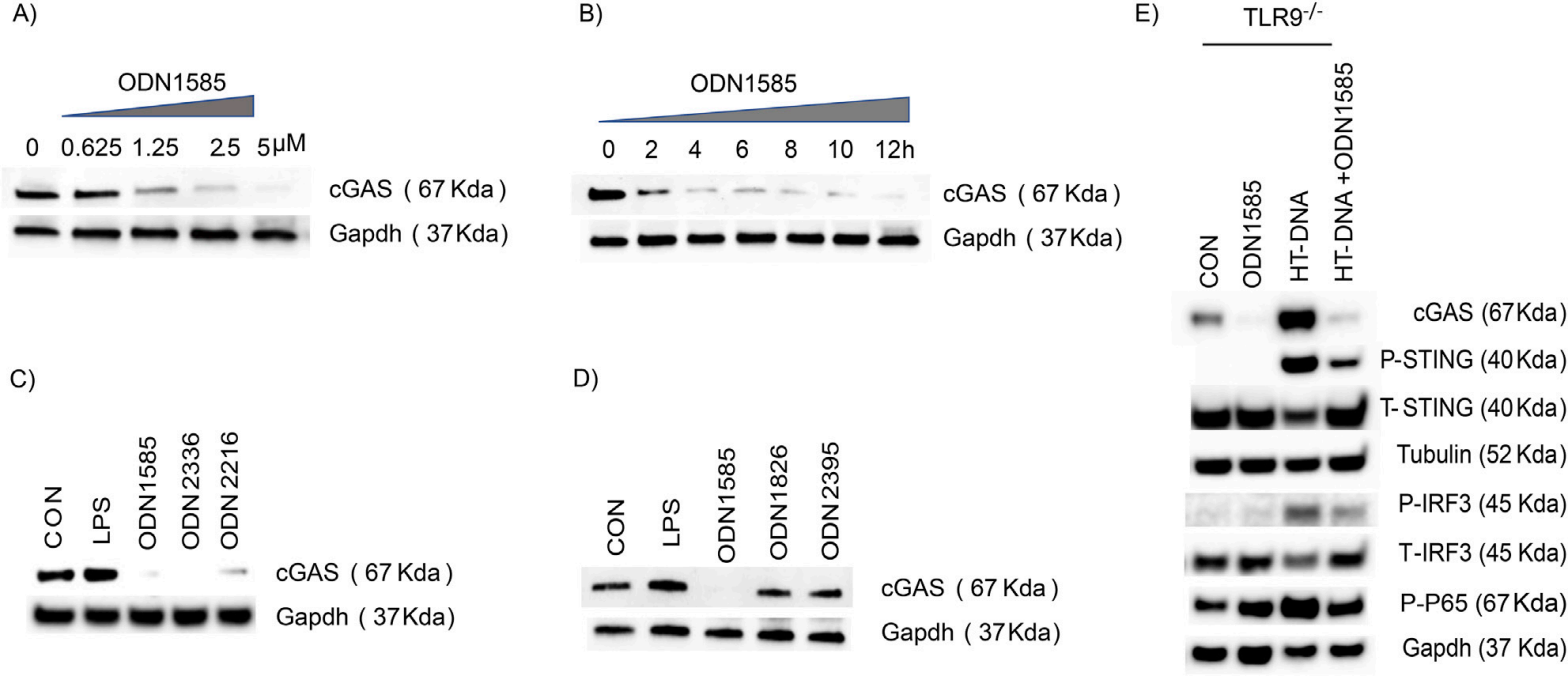
Class A ODNs downregulate the cGAS level in FRC in a TLR9-independent manner **(A–D)** The level of cGAS and GAPDH in WT-FRC was assessed using Western blot analysis **(A)** WT-FRC were treated with indicated concentrations of ODN1585 (0–5 µM) for 18 h **(B)** WT-FRC were treated with ODN1585 (5 µM) for indicated periods (0–12 h) **(C)** WT-FRC were treated with PBS(CON), LPS (1 μg/mL), ODN1585 (5 µM), ODN2336 (5 µM), or ODN2216 (5 µM) for 18 h **(D)** WT-FRC were treated with PBS(CON), LPS (1ug/mL), ODN 1826 (5 µM), or ODN2395 (5 µM) for 18 h. **(E)** The levels of cGAS, p-STING, T-STING, Tubulin, p-IRF3, T-IRF3, p-P65, and GAPDH were assessed in TLR9^−/−^FRC after treatment of PBS(CON), ODN1585 (5 µM), HT-DNA (1 μg/mL), or combined ODN1585 and HT-DNA for 18 h. Data are representative blots of at least two repeated experiments.

We observed that treatment of ODN1585 decreased levels of cGAS in FRC above (Figures 1A,B). To further understand the regulation of class A ODN in the FRC cGAS level, we treated WT FRCs with ODNA1585 at doses ranging from 0.625 µM to 5 µM for 18 h. The levels of cGAS in FRCs decreased in a dose-dependent manner (Figure 2A). Using the highest dose of ODN1585 (5 µM), we found that the levels of cGAS started to decrease as early as 2 h after ODN1585 treatment and were barely detected after 12 h of treatment (Figure 2B). To test if other class A ODNs decreased cGAS levels in FRC, we treated WT FRC with two different class A ODNs, ODN2336 and ODN2216. Treatment with these class A ODNs also decreased the cGAS level in FRC (Figure 2C). To test the effect of other classes ODN on the cGAS level in FRCs, we treated WT-FRC with ODN1585, ODN 1826 (a class B ODN), ODN2395 (a class C ODN), or LPS (a TLR4 agonist serving as a TLR control). We found that only ODN1585, but not ODN1826, ODN2395, or LPS, decreased cGAS levels in FRCs (Figure 2D). Class A ODN is known as an agonist for the TLR9 DNA sensing pathway (Bode et al., 2011). To test if the class A ODN activates the TLR9 signaling to downregulate the cGAS levels in FRC, we treated Tlr9^−/−^FRC with PBS control, ODN1585, HT-DNA, or combined HT-DNA and ODN1585 for 18 h. Of note, we found that treatment of ODN1585 decreased the levels of cGAS and its downstream activation signaling in Tlr9^−/−^FRC (Figure 2E). Together, we have demonstrated that class A ODN downregulated cGAS levels in FRCs in a TLR9-independent manner.

### Class A ODNs downregulate the cGAS level in FRC but not in other cell types

**FIGURE 3:**
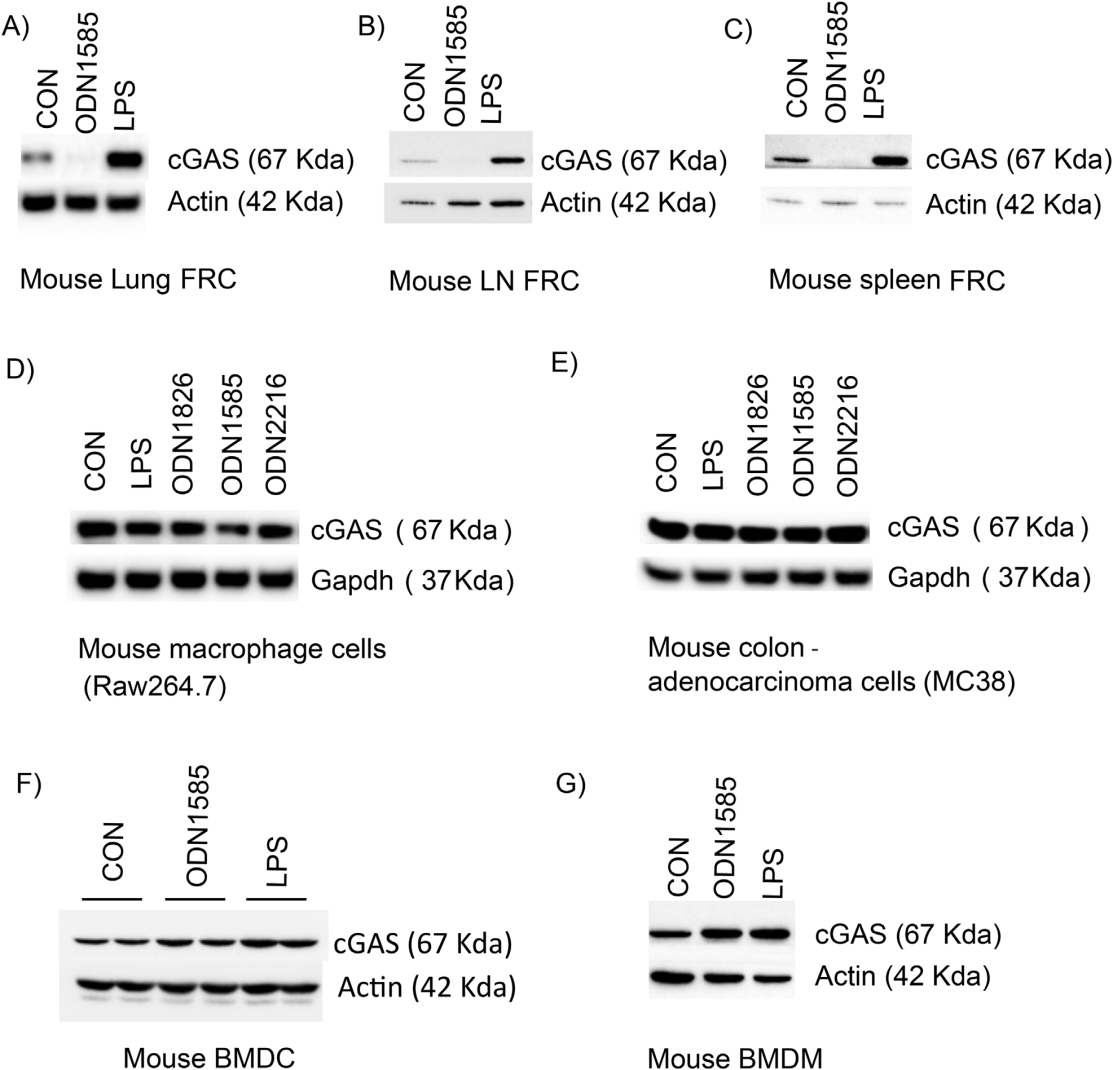
Class A ODNs downregulate the cGAS level in FRC but not in other cell types. The cGAS level was assessed using Western blot in indicated cell types after treatment of PBS (CON), ODN 1826 (5 µM), ODN1585 (5 µM), ODN2216 (5 µM) or LPS (1 μg/mL) for 18 h **(A)** Mouse lung FRC **(B)** Mouse lymph node FRC **(C)** mouse spleen FRC **(D)** Raw267.4 cells **(E)** MC38 cells **(F)** mouse bone marrow-derived dendritic cells **(G)** mouse bone marrow-derived macrophages. Data are representative blots of at least two repeated experiments.

To test if class A ODNs downregulate the cGAS level in FRC of other locations, we isolated FRCs from the lung, lymph node, and spleen, and treated them with PBS control, ODN1585, or LPS for 18 h. Consistently, levels of cGAS decreased in FRCs from the lung, lymph nodes, and the spleen after ODN1585 treatment, whereas increased after LPS treatment (Figures 3A–C; Supplementary Figures S2A–C). To test if class A ODNs regulate the cGAS level in other cell types, we treated Raw264.7 cells, a mouse macrophage cell line, and MC38, a mouse adenocarcinoma cell line, with PBS control, LPS, ODN1826, ODN1585, and ODN2216 for 18 h. Levels of cGAS in Raw264.7 or MC38 did not decrease after ODNs or LPS treatment (Figures 3D,E; Supplementary Figures S2D,E). Furthermore, we generated bone marrow-derived dendritic cells (BMDC) and bone marrow-derived macrophages (BMDM) from WT mice and treated them with PBS control, LPS, and ODN1585. The cGAS level in BMDC and BMDM did not decrease after ODN1585 or LPS treatment (Figures 3F,G; Supplementary Figures S2F,G). These data indicate that Class A ODN negatively regulated cGAS levels in FRCs but not in other cell types.

### Class A ODN does not downregulate cGAS in FRC at transcriptional or translational levels

**FIGURE 4:**
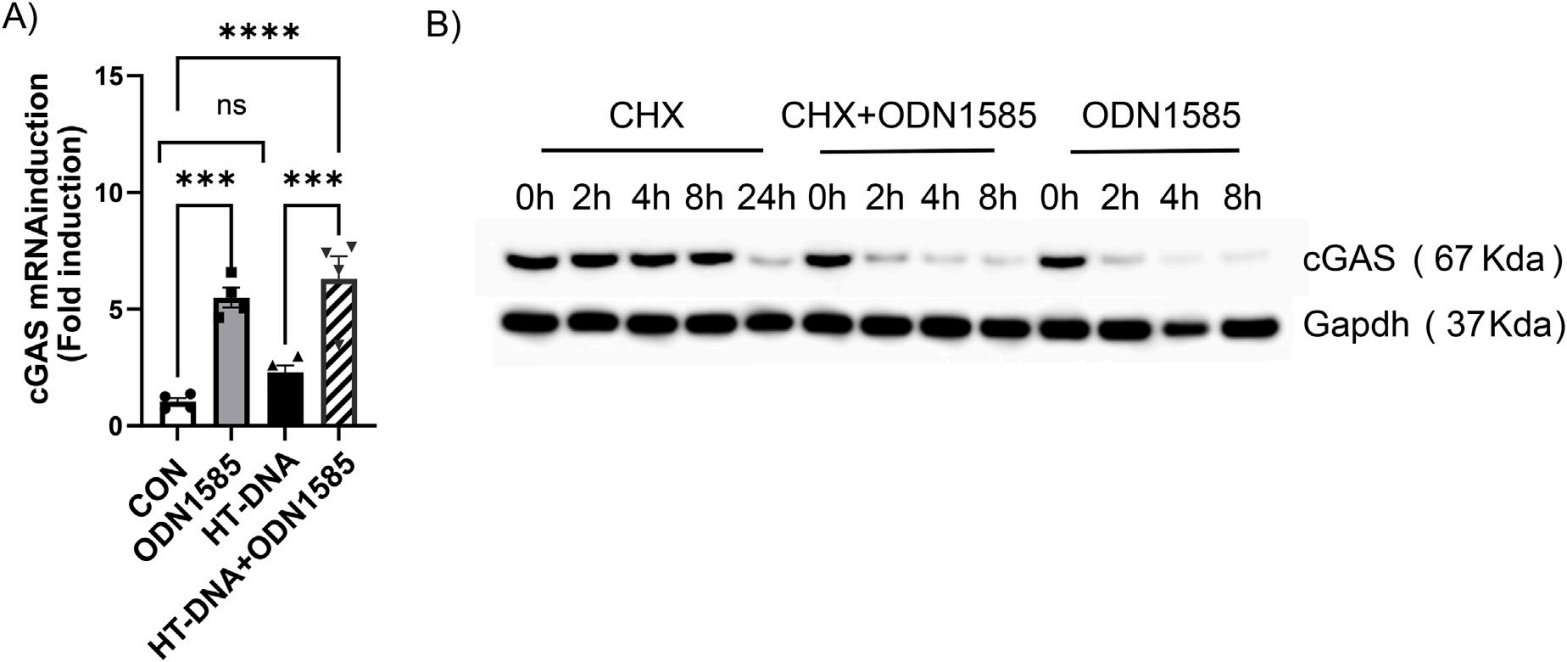
Class A ODN does not downregulate cGAS in FRC at transcriptional or translational levels **(A)** Expression levels of cGAS were assessed using qPCR in WT-FRCs treated with PBS (CON), ODN1585 (5 µM), HT-DNA (1 μg/mL), or a combination of ODN1585 and HT-DNA for 18 h. Graph presented as fold induction relative to untreated control. Experiments have been repeated at least twice, and data are shown as mean ± SEM. Symbols indicate individual values of biological replicates. ***p < 0.001, ****p < 0.0001, ns = not significant **(B)** The cGAS level was assessed using Western blot in WT-FRCs treated with cycloheximide (CHX, 40 µM) alone, CHX + ODN1585, or ODN1585 (5 µM) alone for the indicated periods. Data are representative blots of at least two repeated experiments.

To test if class A ODN regulates the transcriptional process of cGAS in FRC, we treated WT-FRC with PBS control, ODN1585, HT-DNA, or ODN1585+HT-DNA for 18 h and assessed the cGAS expression level using qPCR. Interestingly, the cGAS RNA expression level substantially increased after ODN1585 and ODN1585+HT-DNA treatment compared with the control or HT-DNA treatment (Figure 4A). These suggest that class A ODN transcriptionally upregulates but not downregulate the cGAS mRNA level in FRC. To determine whether class A ODN regulates the translational process of cGAS in FRC, we first treated WT-FRCs with cycloheximide, a protein synthesis inhibitor, with or without ODN1585. The cGAS protein remained at similar levels after 8 h and decreased 24 h after cycloheximide treatment (Figure 4B). The addition of ODN1585 to cycloheximide treatment induced a rapid decrease in cGAS protein levels as early as 2 h after treatment, consistent with ODN1585-only treatment-induced cGAS reduction in FRCs (Figure 4B). These data indicate that class A ODN does not transcriptionally or translationally regulate the cGAS level in FRC.

### Class A ODNs downregulate the cGAS level via interacting with cGAS and ZNF598

**FIGURE 5:**
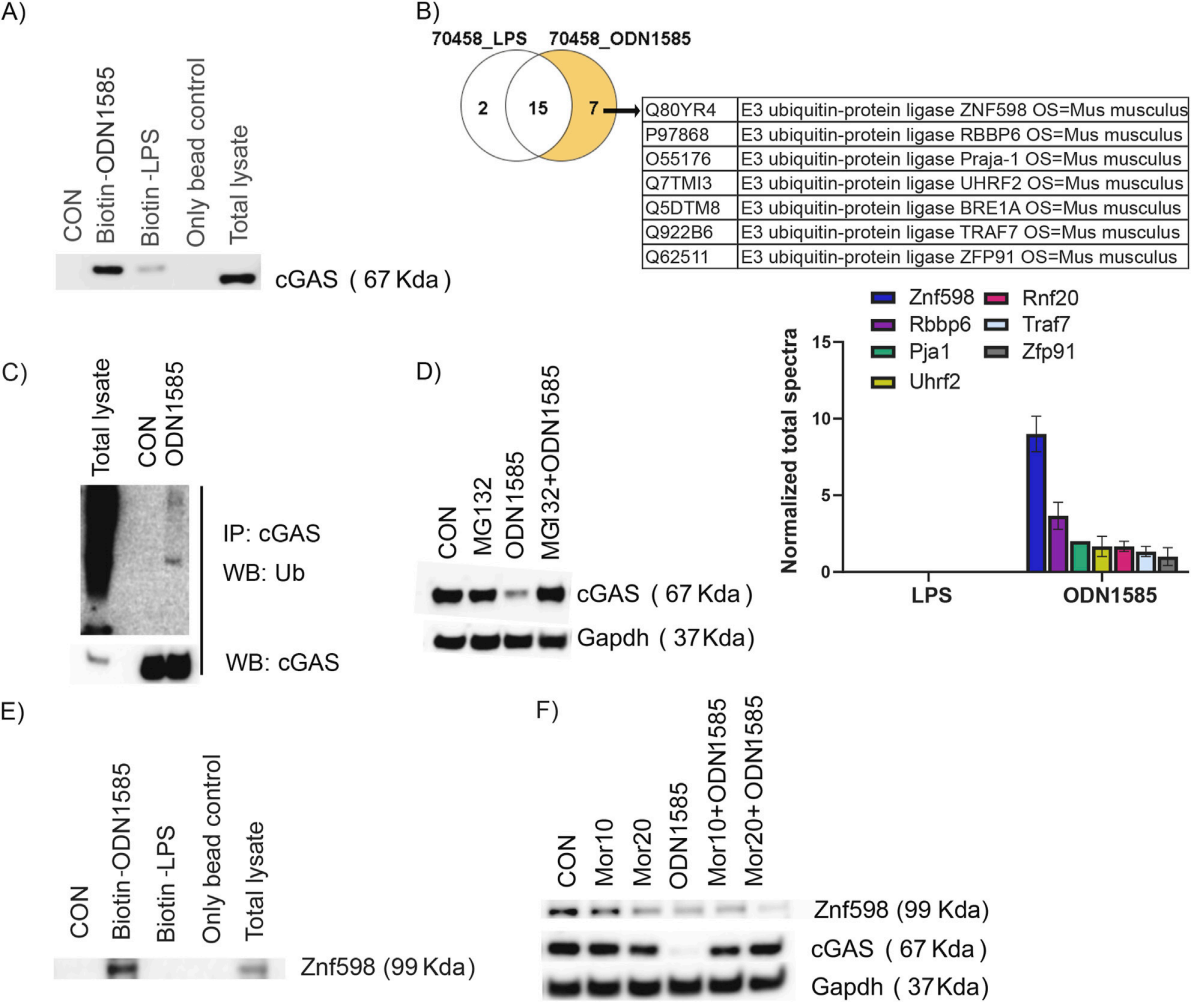
Class A ODN downregulates cGAS levels *via* interacting with cGAS and ZNF598 **(A)** Total cell lysates from FRCs were subjected to pulldown analysis using 5 μg of 3′-biotinylated ODN1585/LPS captured by streptavidin beads. Only bead and Whole lysate were included as controls. Immunoblots were probed for the presence of cGAS **(B)** Venn diagram and table showing ODN1585 associated with 7 E3 ubiquitin-protein ligases identified by mass spectrometry. Bar diagram showing quantification of E3 ubiquitin-protein ligases associated with ODN1585 **(C)** Immunoprecipation analysis of the cGAS-Ubiquitination in FRC treated with or without ODN1585 (5 µM) for 30 min **(D)** The level of cGAS was assessed using Western blot analysis in WT-FRCs treated with PBS (CON), MG132 (2.5 µM), ODN1585 (5 µM), or MG132 + ODN1585 **(E)** Total cell lysates from FRCs were subjected to pulldown analysis using 5 μg of 3′-biotinylated ODN1585/LPS and immunoblots were probed for the presence of ZNF598 **(F)** The levels of ZNF598 and cGAS were assessed using Western blot analysis in WT-FRCs treated with PBS (CON), Morpholinos (Mor10 μM or 20 μM), ODN1585, Mor (10 μM or 20 μM) + ODN1585. Data are representative blots of at least two repeated experiments.

Our results suggest class A ODN may post-translationally regulate cGAS levels in FRC. To test if class A ODNs interact with cGAS, we performed pulldown assays using biotinylated-ODN1585 to pulldown the associated proteins in WT-FRC cell lysate. Interestingly, we found that cGAS was pulled down by biotinylated-ODN1585, but not by biotinylated-LPS (biotin control) or beads-only control (Figure 5A). To further understand how class A ODNs regulate the cGAS level in FRC, we treated WT-FRCs with biotinylated-class A ODN and biotinylated-LPS for 1 h and subjected the cell lysates for proteomic analysis. Compared with biotinylated-LPS, class A ODN was uniquely associated with 7 E3 ubiquitin-protein ligases (Figure 5B). To test if class A ODNs regulate the cGAS level via E3 ubiquitin-protein ligases, we immunoprecipitated ODN1585-treated or control FRC protein lysate with anti-cGAS antibody and immunoblotted for cGAS-ubiquitin to assess the ubiquitination levels of cGAS in response to ODN1585 treatment. Notably, we found that treatment of ODN1585 increased the ubiquitination levels of cGAS compared with the control (Figure 5C). Furthermore, addition of MG-132, a potent proteasome inhibitor, inhibited ODN1585-induced decrease of cGAS levels (Figure 5D). As ZNF598 is one of the E3 ubiquitin-protein ligases identified by proteomic analysis with the highest association (Figure 5B). We next performed a pulldown assay with biotinylated-ODN1585 in WT-FRC cell lysate, we found that ZNF598 was pulled down by biotinylated-ODN1585, indicating that ZNF598 was associated with ODN1585 (Figure 5E). To test if ZNF598 is involved in class A ODN-induced downregulation of cGAS, we knockdown ZNF598 in FRC using Morpholino (Mor) antisense oligonucleotides. Of note, knockdown of ZNF598 inhibited ODN1585-induced decrease of cGAS levels (Figure 5F). These results suggest that Class A ODNs downregulate the cGAS level via interacting with cGAS and ZNF598.

### Class A ODNs suppress the cGAS levels and signaling in human fibroblasts

**FIGURE 6:**
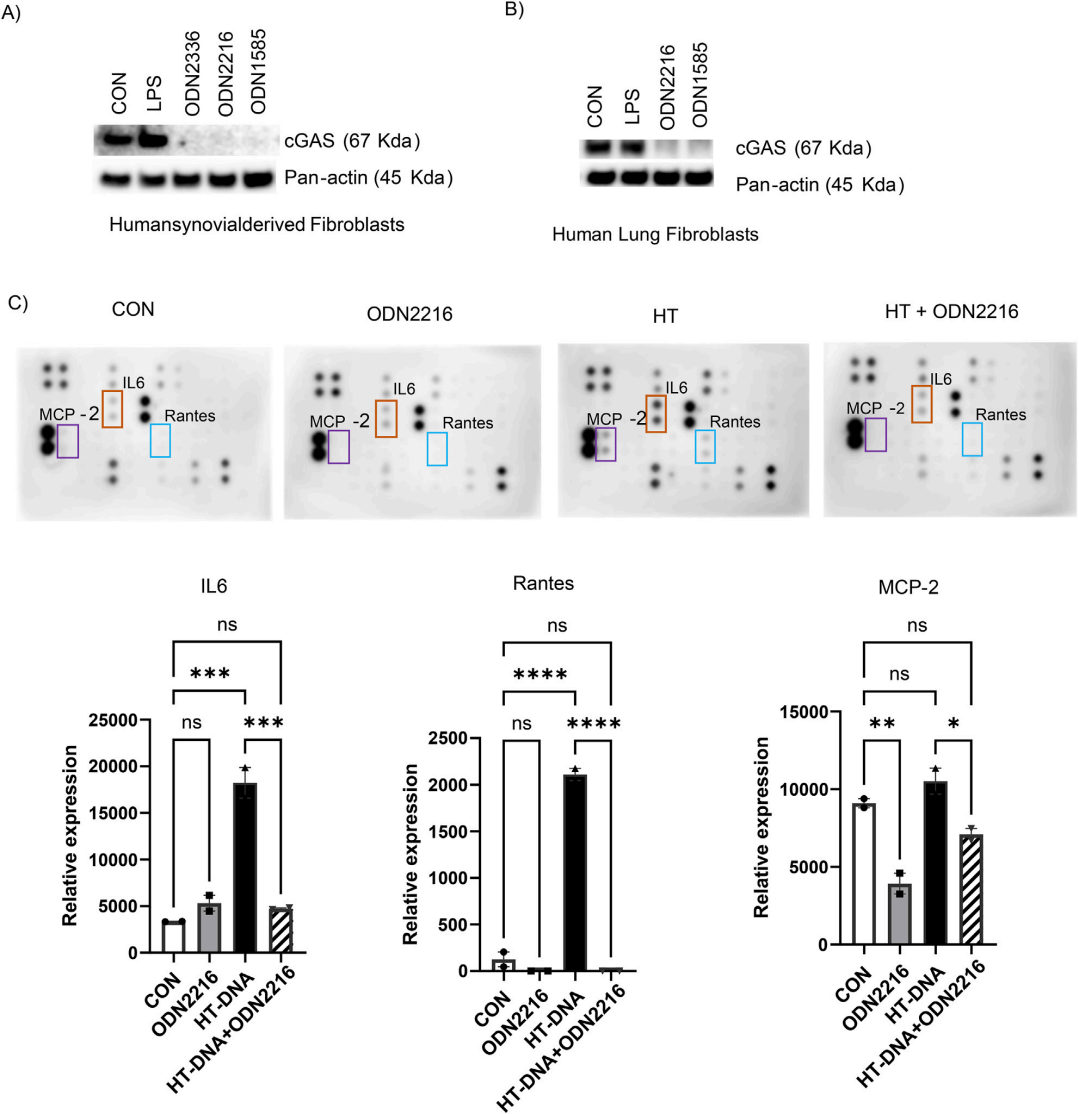
Class A ODNs suppress the cGAS levels and signaling in human fibroblasts. The level of cGAS was assessed using Western blot analysis in WT-FRCs treated with PBS (CON), LPS, ODN2216, or ODN1585 **(A)** human synovial-derived fibroblasts and **(B)** human lung fibroblasts (HLFs) **(C)** The supernatants were collected from HLFs treated with PBS (CON), ODN2216, HT-DNA, or HT-DNA + ODN2216 and subjected to cytokine antibody array. The relative expression values of IL6, Rantes, and MCP-2 from the cytokine array membrane were evaluated by densitometry and represented as graphs. Experiments have been repeated at least twice. Data are shown as mean ± SEM of one representative experiment. *p < 0.05, **p < 0.01, ***p < 0.001, ****p < 0.0001, ns = not significant.

To test if class A ODNs suppress the cGAS levels in human fibroblasts, we treated the human synovial fibroblasts and human lung fibroblasts with PBS control, LPS, and three class A ODNs (ODN1585, a mouse preferred TLR9 agonist, and ODN2336 and ODN2216, human preferred TLR9 agonists) for 18 h. Consistently, the level of cGAS decreased substantially after all 3 class A ODNs treatment (Figures 6A,B). Performing cytokine array to assess the cytokine levels in the media of human lung fibroblasts, we found that treatment with HT-DNA increased levels of IL-6, Rantes, MCP-2 (Figure 6C), which has been reported to be regulated by the cGAS signaling (Liu et al., 2022; Chu et al., 2024). Whereas treatment of ODN2216 did not increase the levels of cGAS-mediated cytokines (Figure 6C). Interestingly, the addition of ODN2216 suppressed HT-DNA-induced increase in levels of IL-6, Rantes, MCP-2 in the media (Figure 6C). In summary, these results indicate that class A ODNs suppress the cGAS signaling in human fibroblasts.

## Discussion

In this study, we found that class A ODN, but not class B or C ODNs, negatively regulated the cGAS level and cGAS-mediated inflammatory response in mouse and human FRC. Unexpectedly, the suppressive effect of class A ODN on the cGAS level is limited to FRC but not in immune cells, such as macrophage and dendritic cells, and other non-immune cells, such as the M38 murine colon adenocarcinoma cell line. Mechanistically, class A ODN interacts with cGAS and ZNF598 to downregulate the cGAS levels.

The TLR9 and cGAS DNA pathways are two major signaling pathways detecting self and non-self DNA to mount immune responses against infection and tumorigenesis (Motwani et al., 2019). We have shown here that class A ODN physically associates with cGAS and downregulates the cGAS level as early as 2 h after treatment. The downregulation of cGAS in FRC only happens in response to class A ODN treatment but not class B or C. TLR9 recognizes sequence-specific single-stranded DNA containing unmethylated CpG motifs (Hemmi et al., 2000). All four classes of CpG ODNs are synthetic oligodeoxynucleotides containing unmethylated CpG motifs, which are designed to activate TLR9 to mount immune responses (Bode et al., 2011). Canonically, cGAS binds to cytosolic double-stranded (ds) DNA in a sequence non-specific manner (Du and Chen, 2018). However, emerging studies indicate that cGAS can bind to cytosolic ssDNA shorter than 40 basepair (bp) to initiate the downstream signaling (Unterholzner et al., 2010; Jakobsen et al., 2013; Herzner et al., 2015; Bode et al., 2021). Herzner et al. has demonstrated that the minimal cGAS recognition motif consists of a stem-loop structure with 12–20 bp of dsDNA flanked by poly G tails at either side (Herzner et al., 2015). Class A ODN consists of a mixed phosphodiester/phosphorothioate backbone with a single CpG motif flanked by palindromic sequences and poly G tails at the 3′ and 5′ ends forming a stem-loop structure (Krug et al., 2001), which meets the minimal cGAS recognition motif. Consistently, a recent study reported that class A ODN binds to cGAS and activates the downstream signaling in human primary monocyte or monocyte-derived macrophage (Bode et al., 2021). The unique structure of class A ODN allows class A ODN to interact with cGAS directly. In line with this, we have shown here that class A ODNs downregulate cGAS in a TLR9 independent manner. We have previously shown that class A ODN suppresses chemokine production and retinoid metabolism in FRC in a TLR9-dependent manner (Xu et al., 2019; Jiang et al., 2022; Jiang et al., 2024). As the role of cGAS signaling in chemokine production and retinoid metabolism in FRC is unknown, whether class A ODN-induced cGAS disappearance contributes to the suppressive effect on chemokine production and retinoid metabolism in FRC is unclear.

Different from the activation effect of class A ODNs on the cGAS signaling in human monocyte and monocyte-derived macrophages (Bode et al., 2021), we have shown here that class A ODN binds to cGAS and induces cGAS disappearance in murine and human FRCs. Interestingly, the class A ODN-induced cGAS disappearance is only observed in FRC, but not in bone marrow-derived macrophages, bone marrow-derived dendritic cells, or murine and human cancer cell lines. To further understand the mechanisms of how class A ODN induced cGAS disappearance in FRC, we have performed a series of experiments and found that class A ODN does not regulate cGAS at transcriptional or translational levels. Mass spectrum analysis revealed class A ODN was associated with a series of E3 ubiquitin ligases. ZNF598 is one of E3 ubiquitin ligases associated with class A ODNA in FRC. ZNF598 regulates ribosome-associated protein quality control via binding to the 40S-40S interface of collided ribosomes to mediate the ubiquitination of specific small ribosomal subunit proteins in the disomal context (i.e., RPS3, RPS10, RPS20) (Juszkiewicz et al., 2018; Vind et al., 2020). A recent study has shown that ZNF598 regulates cGAS activation in U2OS cells, the human osteosarcoma cell line via modulating ribosome collision and translation stress (Wan et al., 2021). However, our data indicate that class A ODN regulates the cGAS levels in FRC at the post-translational level. Class A ODN interacts with cGAS and ZNF598 in FRC. It is conceivable that the class A ODN may be forming a complex with cGAS and ZNF598. However, future studies are required to understand whether ZNF598 directly interacts with cGAS to induce cGAS ubiqutination, how class A ODN affects the interactions between ZNF598 and cGAS to induce cGAS disappearance, and whether other E3 ubiquitin ligases associated with class A ODN identified in this study are involved in regulating class A ODN-induced cGAS disappearance in FRC.

Class A ODNs have been applied to treat inflammatory diseases and cancer in preclinical studies and clinical trials (Bode et al., 2011). Most of these studies utilize class A ODNs to modulate the immune response in immune cells (Bode et al., 2011). FRC widely exist in both immune and non-immune tissues (Fletcher et al., 2015) controlling the innate and adaptive immunity organs and tissues including lymph nodes (Li et al., 2021), the spleen (Perez-Shibayama et al., 2019), the lung (Cupovic et al., 2021) and tumors (Lin and Gordon, 2023; Onder et al., 2024). We have shown that class A ODN is a potent regulator of the immune functions in FRC during peritonitis (Xu et al., 2019) and cancer metastasis (Jiang et al., 2022). Furthermore, activating the cGAS signaling in cancer-associated fibroblast promotes platinum resistance of ovarian cancer cells (Liu et al., 2024). These data suggest that the cGAS signaling is essential for the cell fate decisions and functions of FRC. Our results indicate that class A ODNs regulate both TLR9 and cGAS signaling in FRC. Therefore, the effect of class A ODNs on FRCs should be considered when designing class A ODN therapies for inflammatory diseases and cancer.

In summary, our results unravel an unrecognized off-target effect of class A ODN on suppressing the cGAS level and signaling in FRC, which provides critical information for designing class A ODN therapies for inflammatory diseases and cancer in the future.

## Data Availability

The raw data supporting the conclusions of this article will be made available by the authors, without undue reservation.

## Supplementary material

SUPPLEMENTARYFIGURE S1 The quantitative analysis of western blots in Figure 1A. **(A)** cGAS protein level normalized to the level of Gapdh. **(B)** p-STING protein level normalized to the level of T-STING and Tubulin. **(C)** p-IRF3 protein level normalized to the level of T-IRF3 and Gapdh. **(D)** p-P65 protein level normalized to the level of Gapdh.

SUPPLEMENTARYFIGURE S2 The quantitative analysis of western blots in Figures 3A–G cGAS protein level normalized to the level of Actin or Gapdh.
