# Supplementary figures and images for: An off-target effect of class A CpG-oligonucleotides on suppressing the cyclic GMP-AMP synthase signaling in fibroblastic reticular cells
Source: Front Pharmacol. 2025 Apr 23;16:1576151. doi: 10.3389/fphar.2025.1576151 (PMC12055788; doi:10.3389/fphar.2025.1576151)

Figure S2

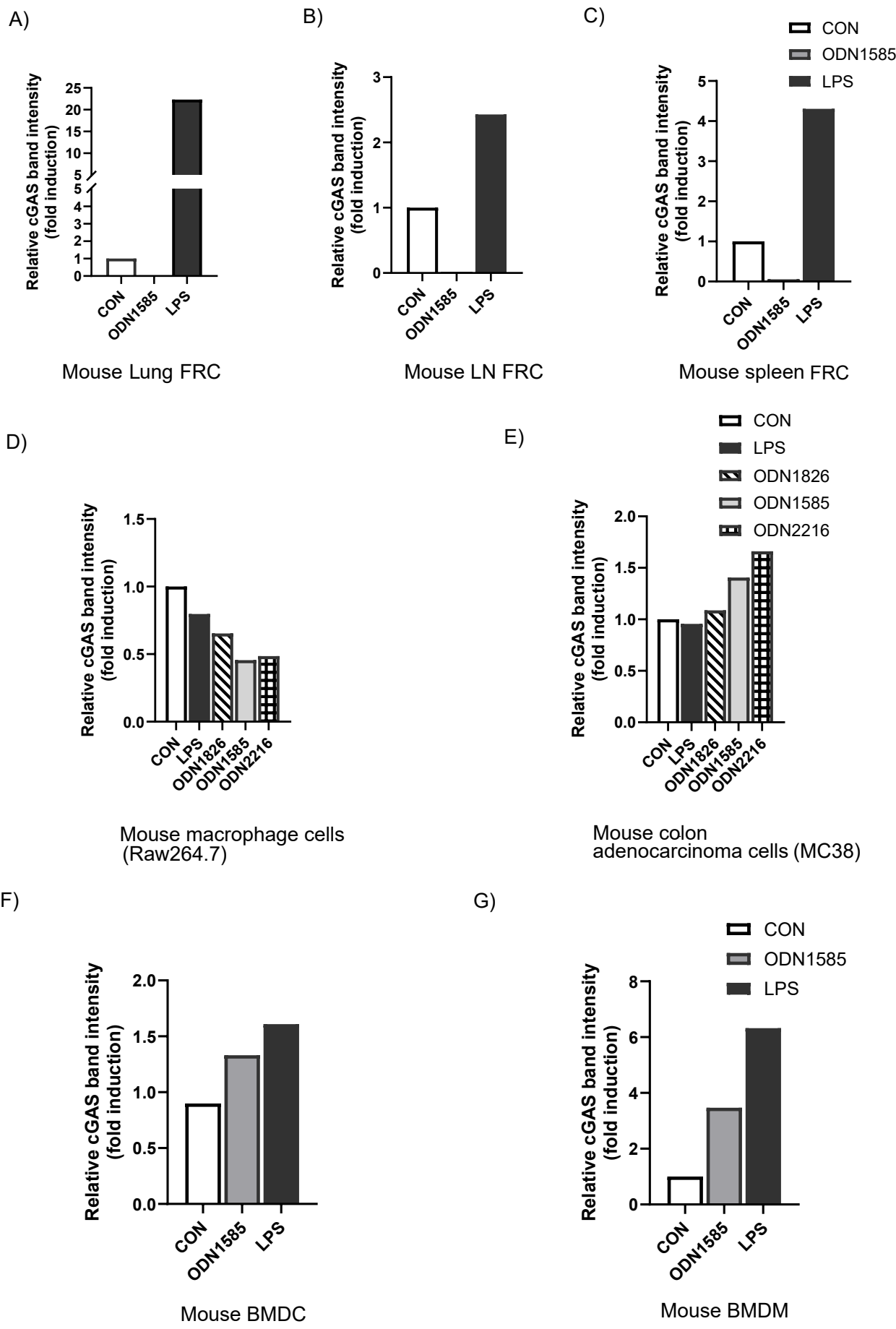

Supplement: Supplementary file 1 [file DataSheet2.pdf]

Figure S1

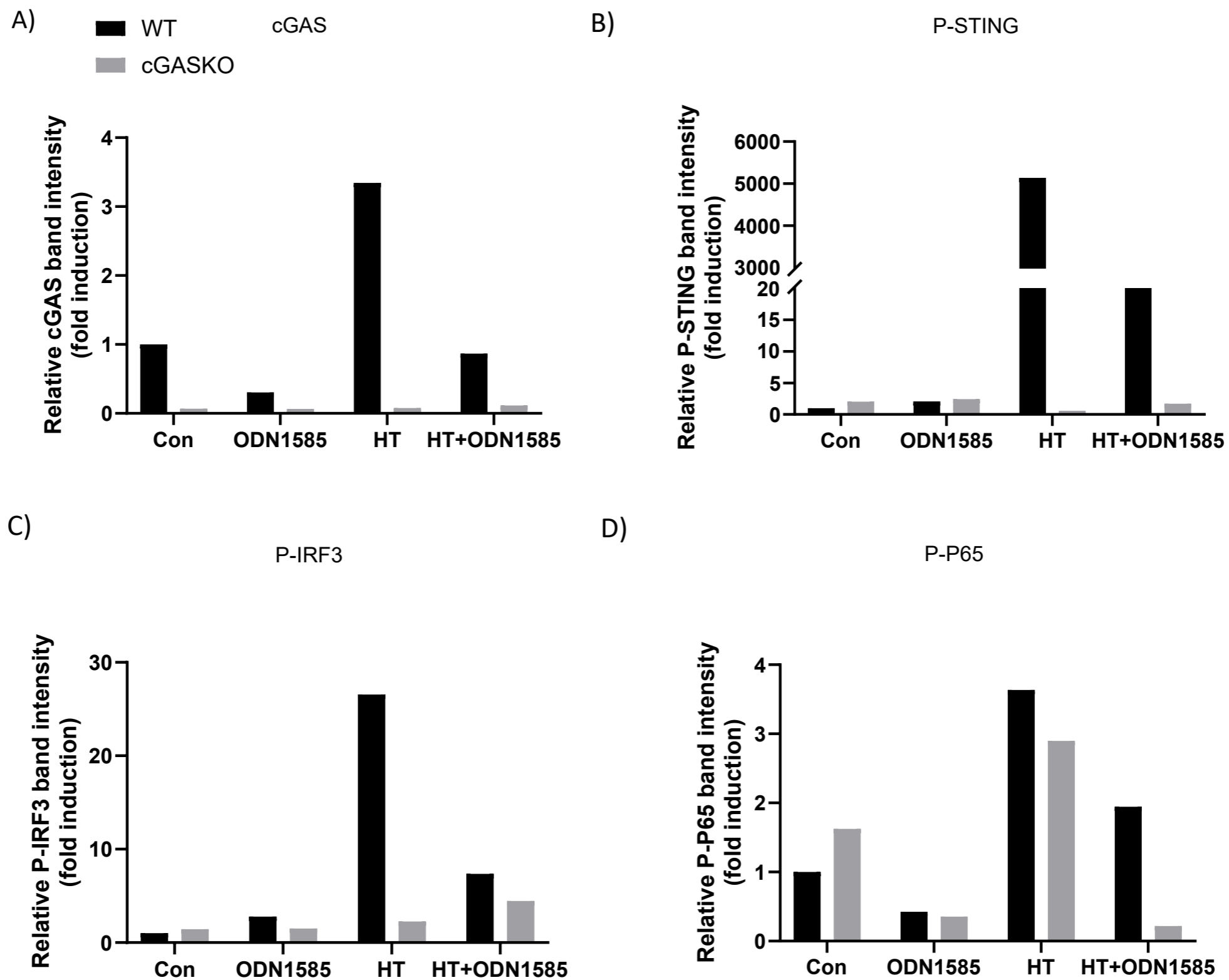

Supplement: Supplementary file 3 [file DataSheet1.pdf]
