# Supplementary material for: An off-target effect of class A CpG-oligonucleotides on suppressing the cyclic GMP-AMP synthase signaling in fibroblastic reticular cells
Source: Front Pharmacol. 2025 Apr 23;16:1576151. doi: 10.3389/fphar.2025.1576151 (PMC12055788; doi:10.3389/fphar.2025.1576151)

Figure 1A & 2E)

T-STING (40Kda)

P-STING (40Kda)


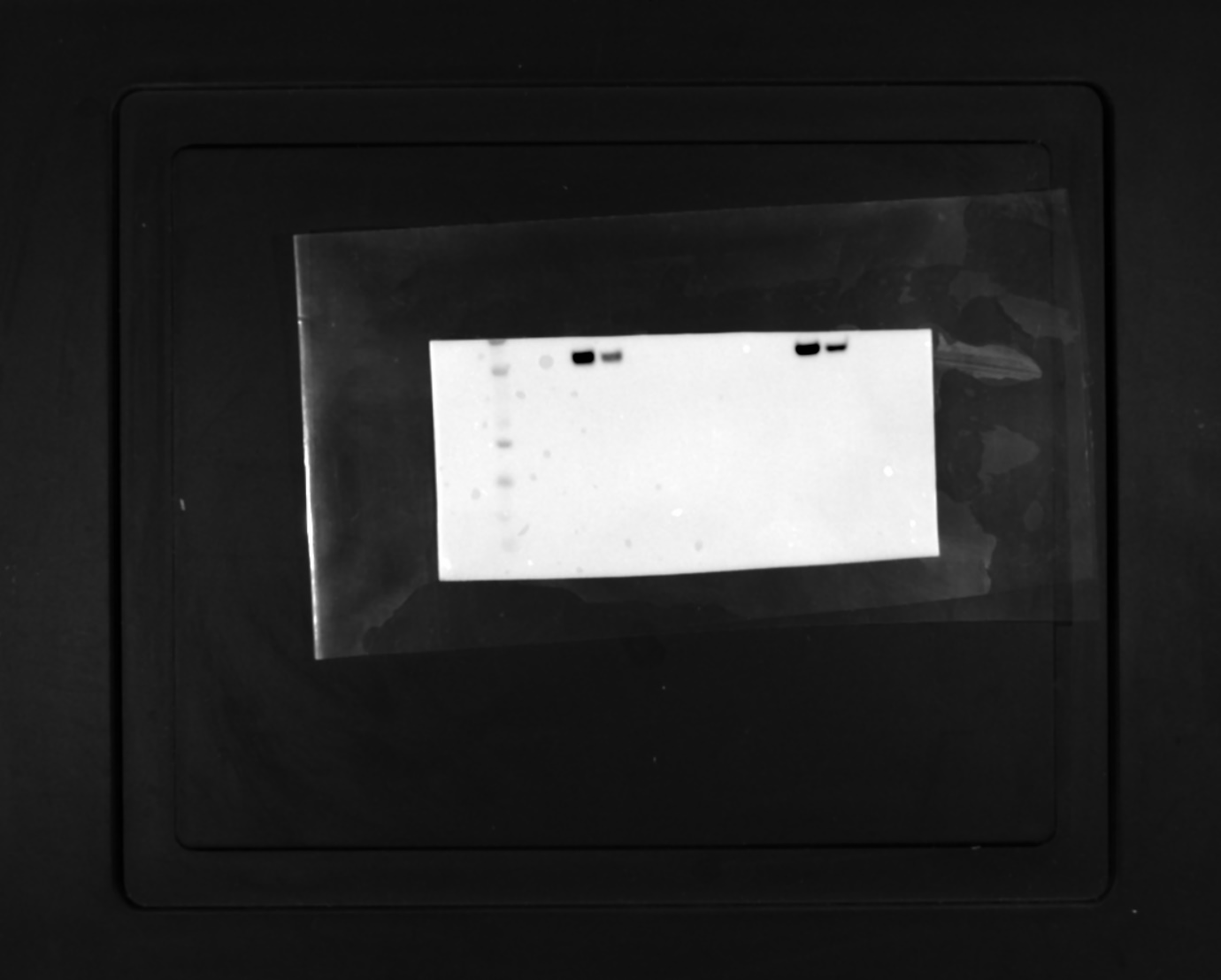

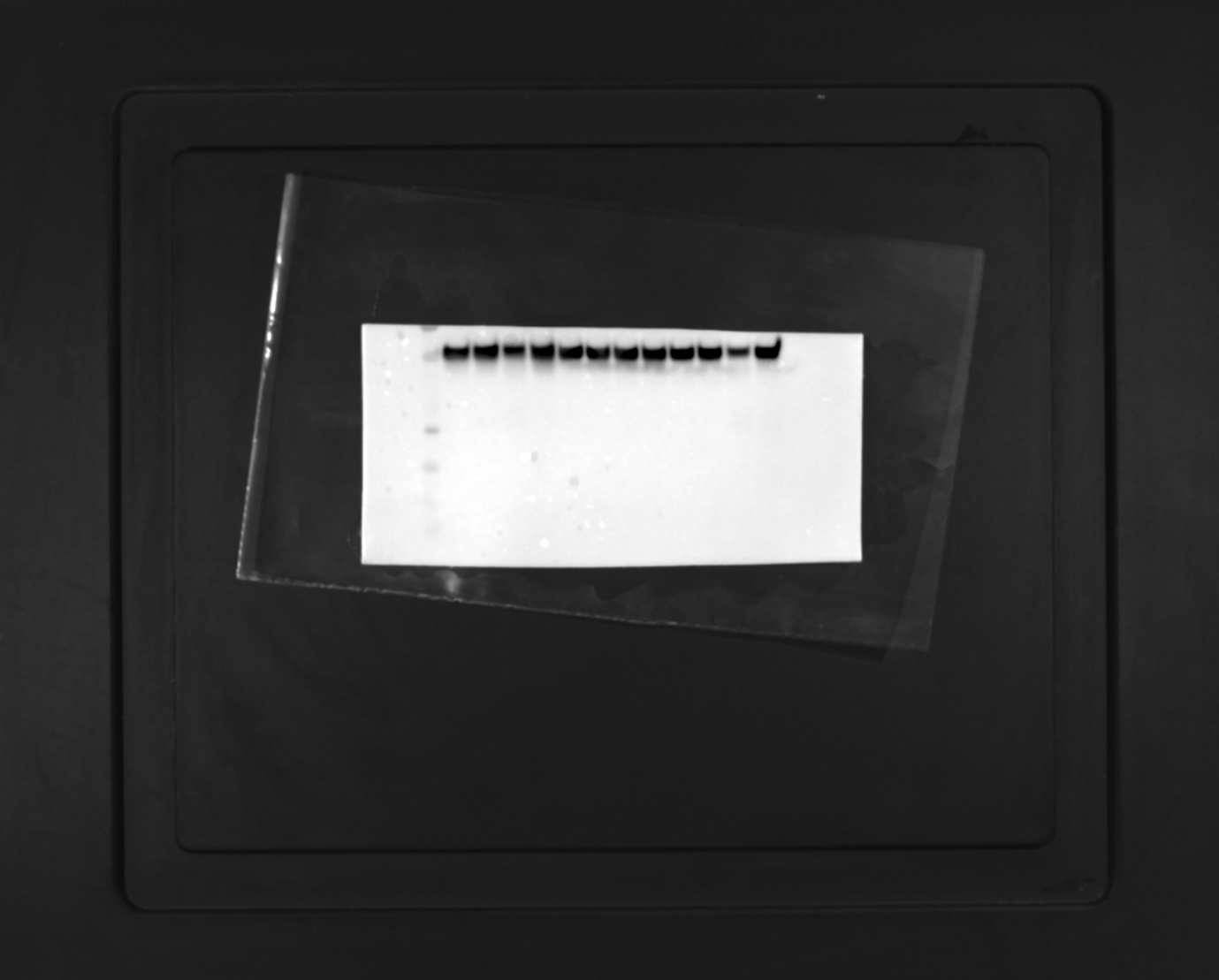


WT

cGAS-/-

TLR9-/-

TLR9-/-

cGAS-/-

WT

37Kda

37Kda

50Kda

T-IRF3 (45Kda)

P-IRF3 (45Kda)


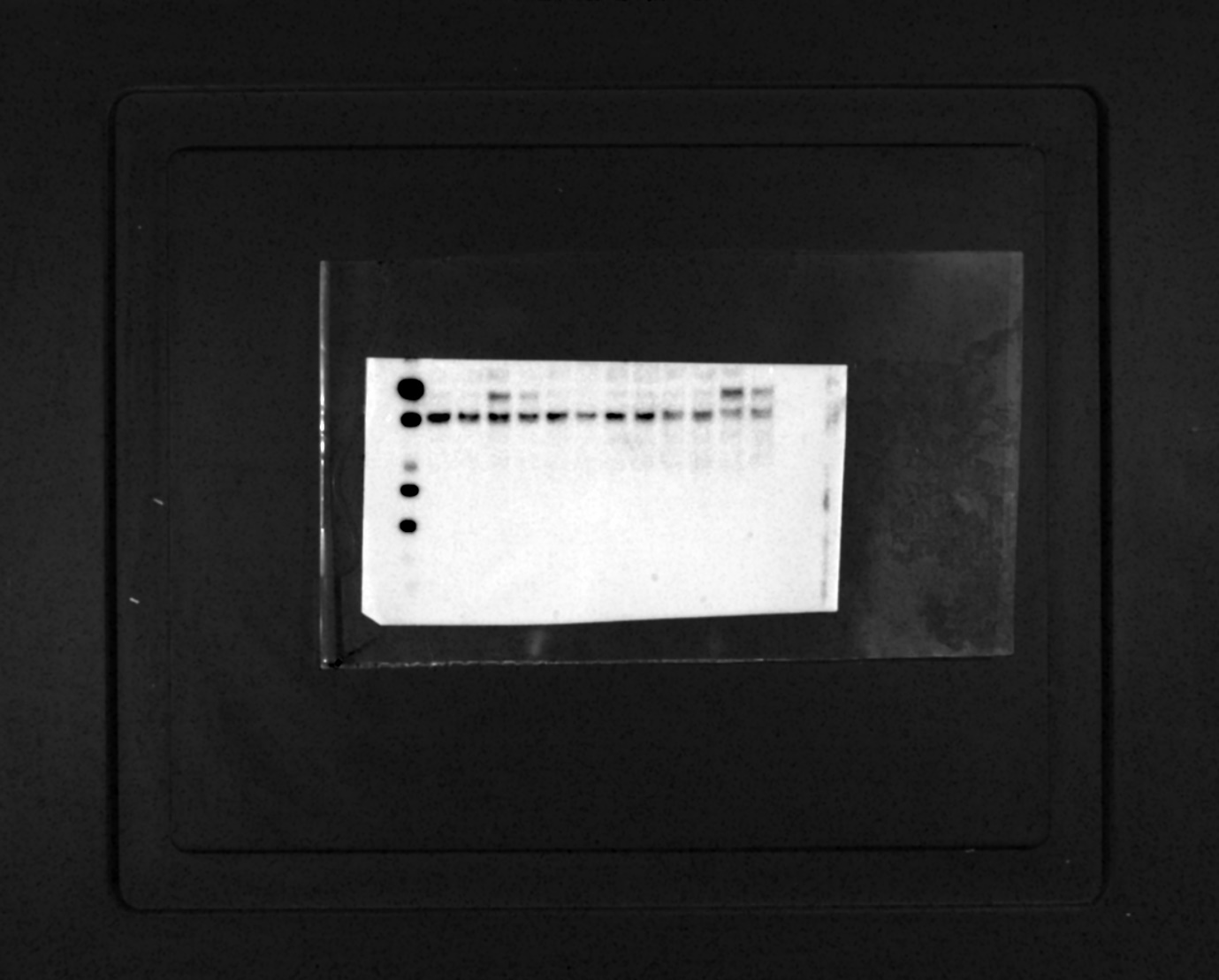

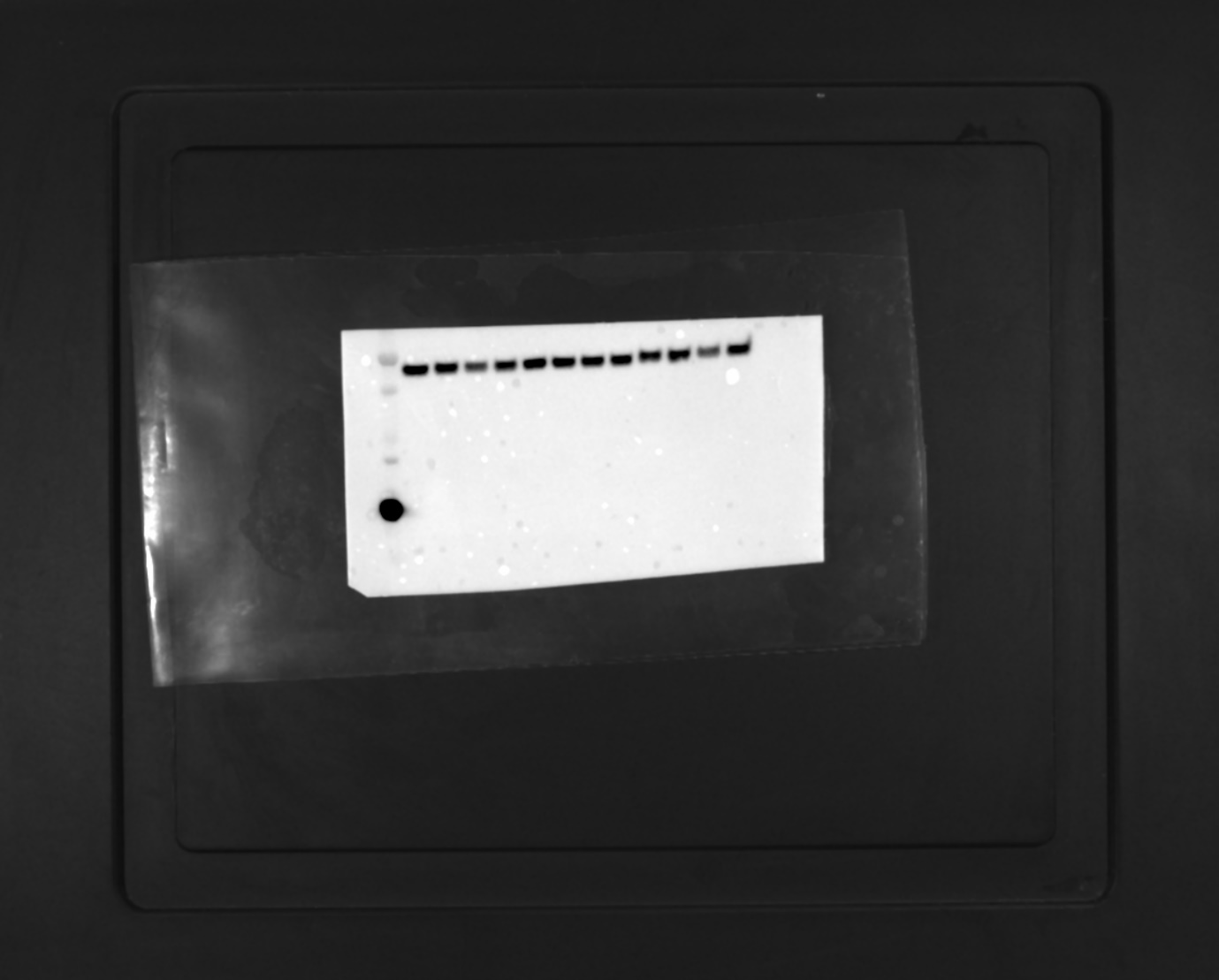


WT

cGAS-/-

TLR9-/-

WT

cGAS-/-

TLR9-/-

37Kda

50Kda

50Kda

37Kda

Gapdh (37Kda)


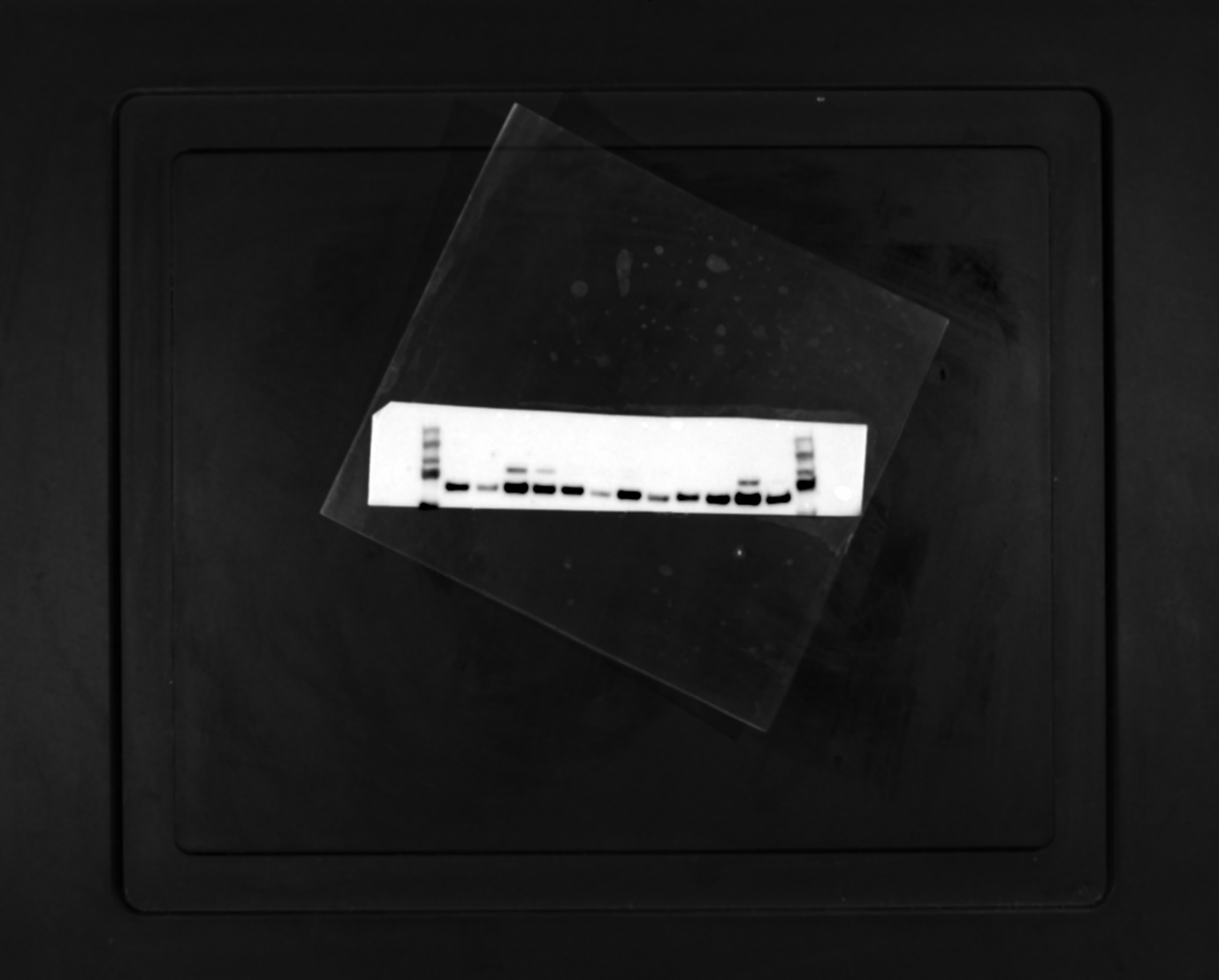

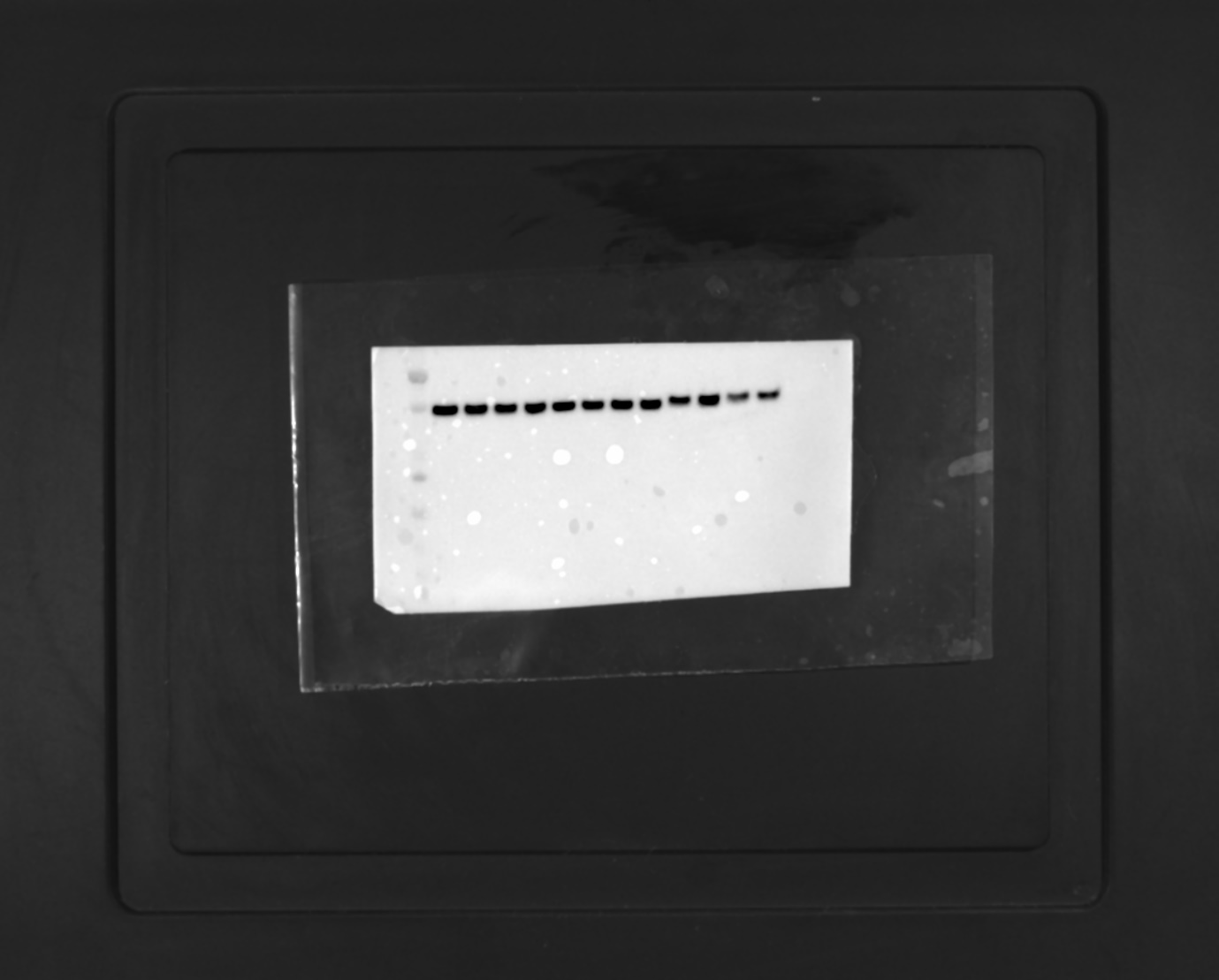


WT

cGAS-/-

TLR9-/-

WT

cGAS-/-

TLR9-/-

37Kda

P-P65 (67Kda)

75Kda

50Kda

Tubulin (52Kda)


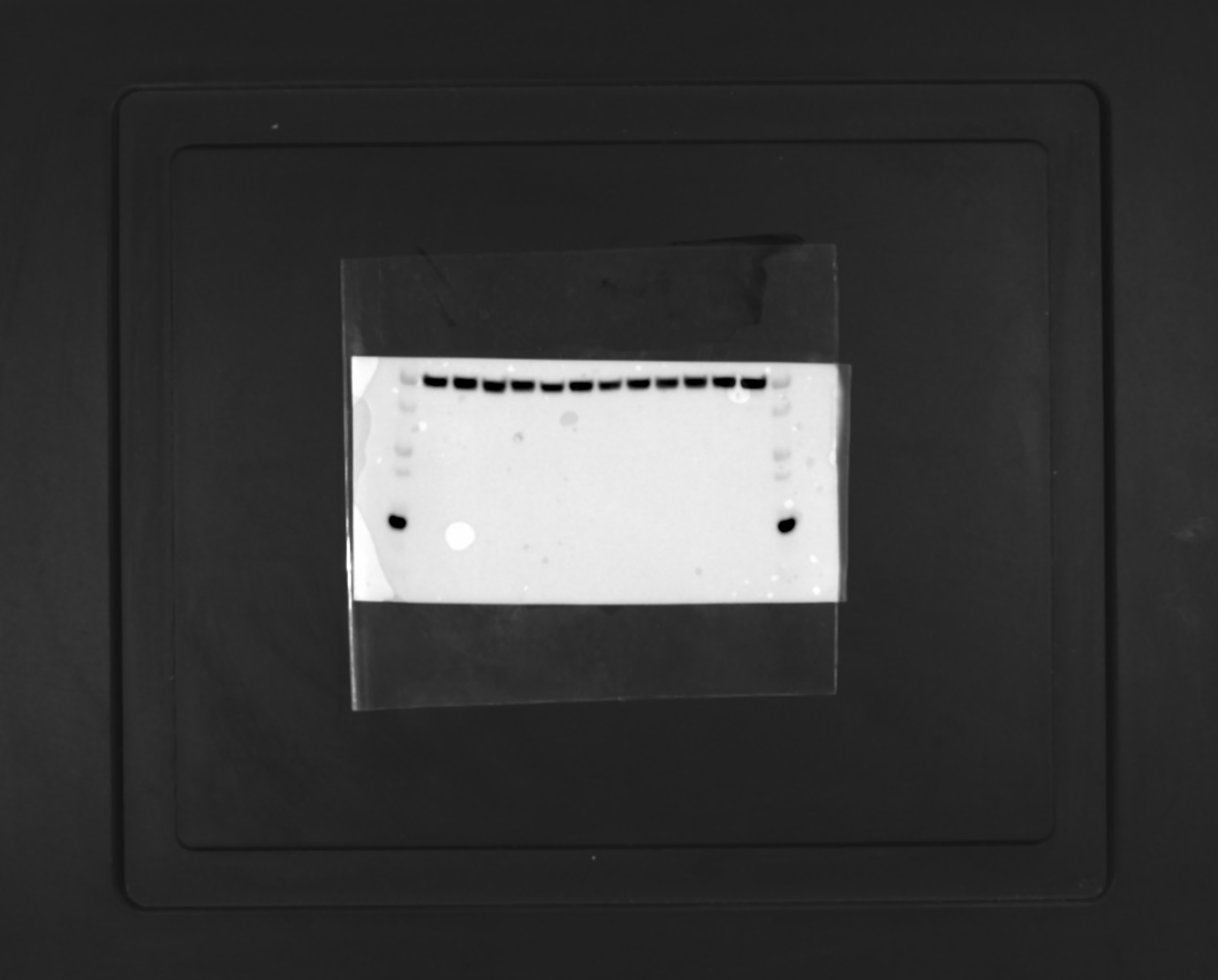

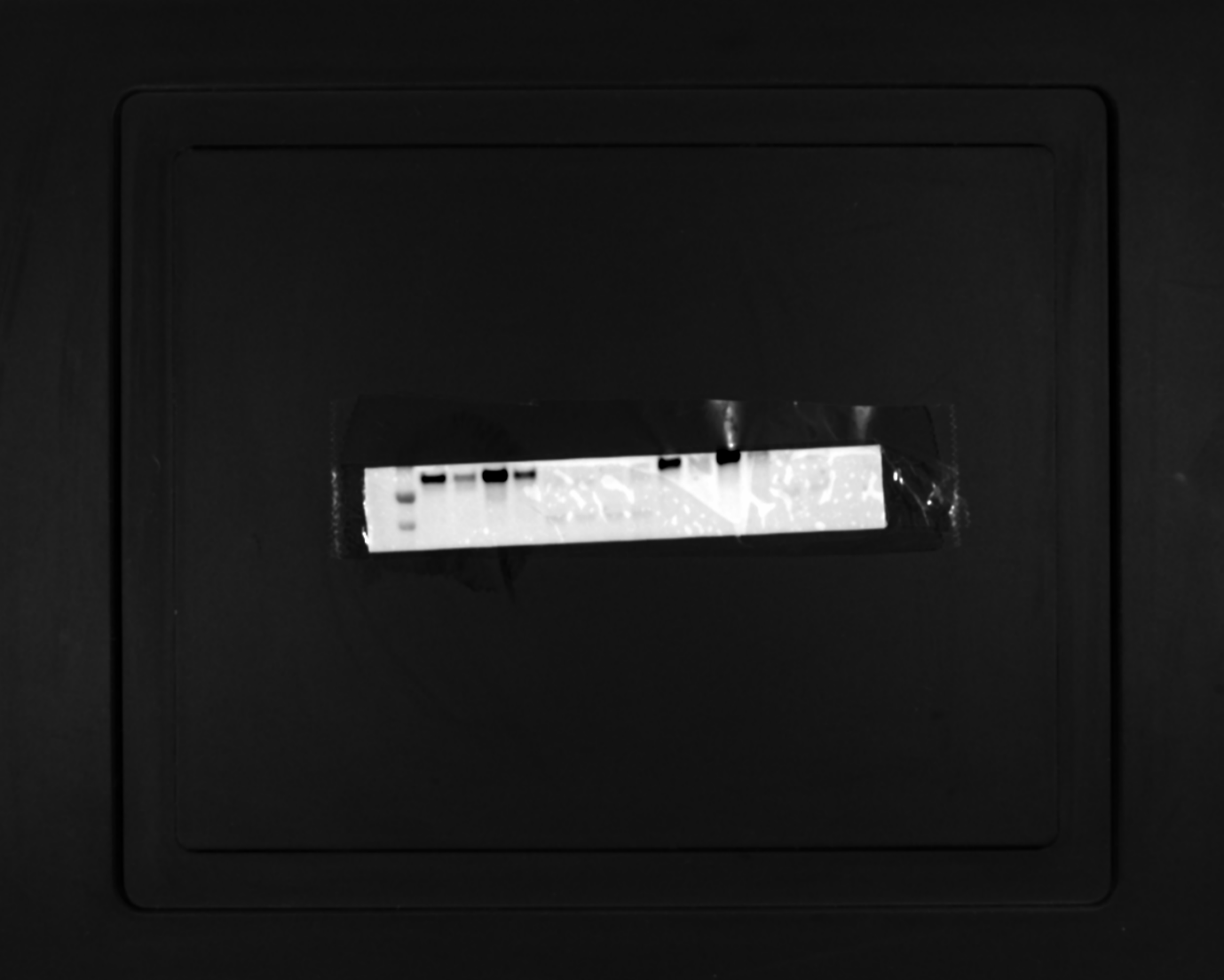

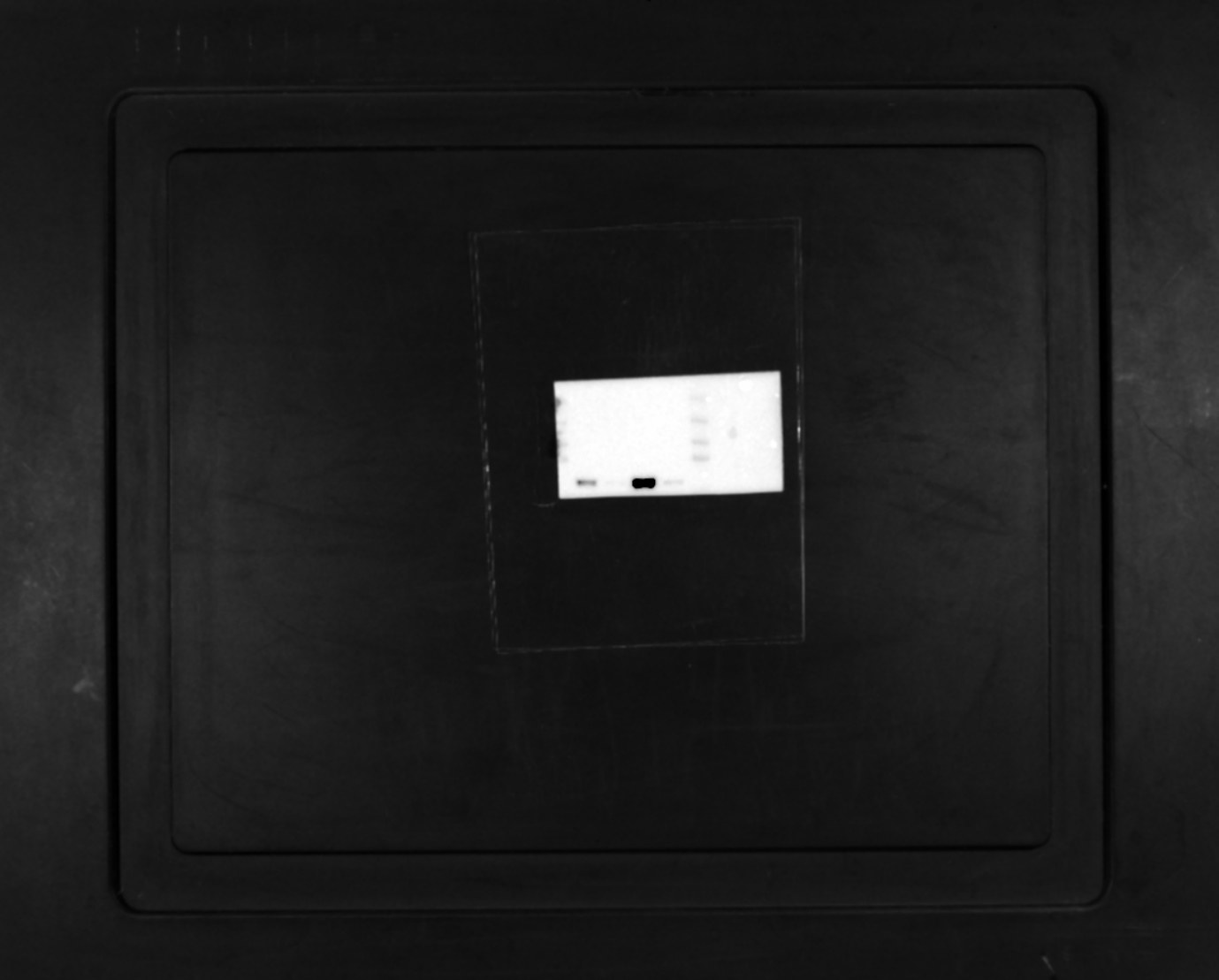


WT

cGAS-/-

TLR9-/-

WT

cGAS-/-

TLR9-/-

50Kda

cGAS

Figure 2

2A)

Gapdh (37Kda)

cGAS(67 Kda)


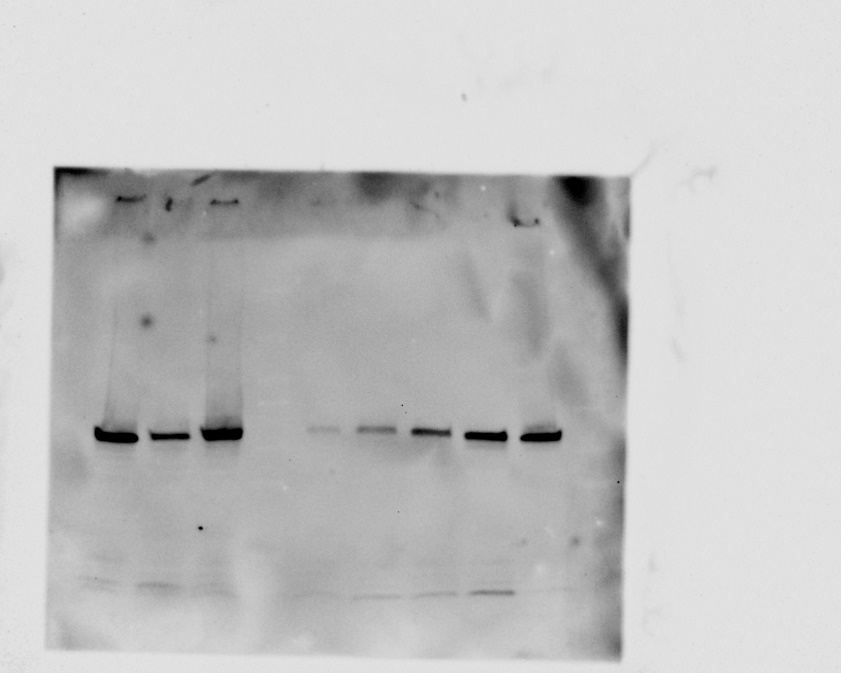

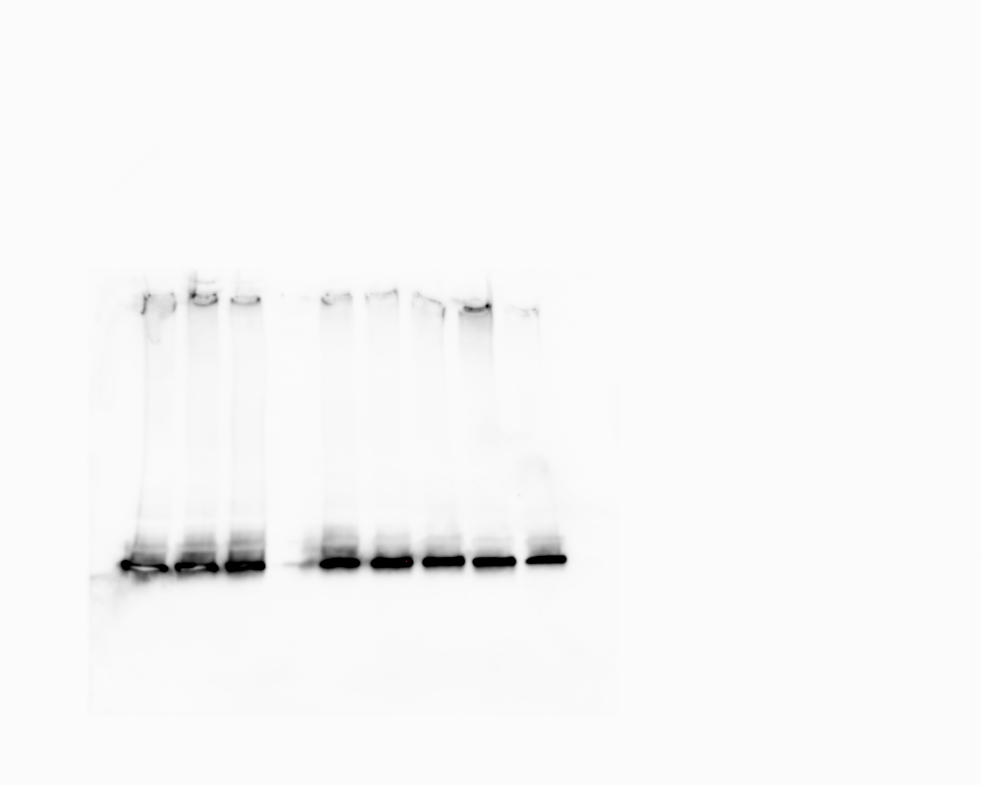


2B)

Gapdh (37Kda)

cGAS (67Kda)


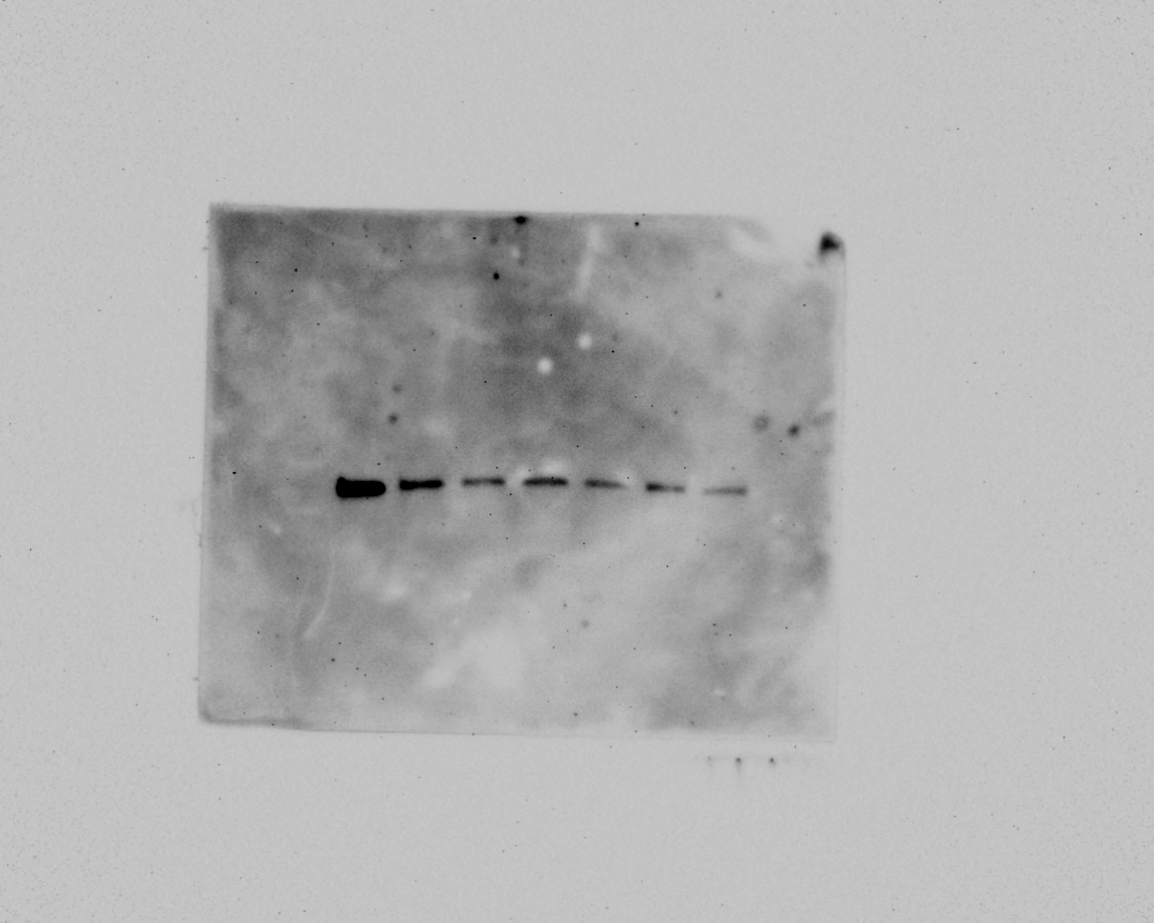

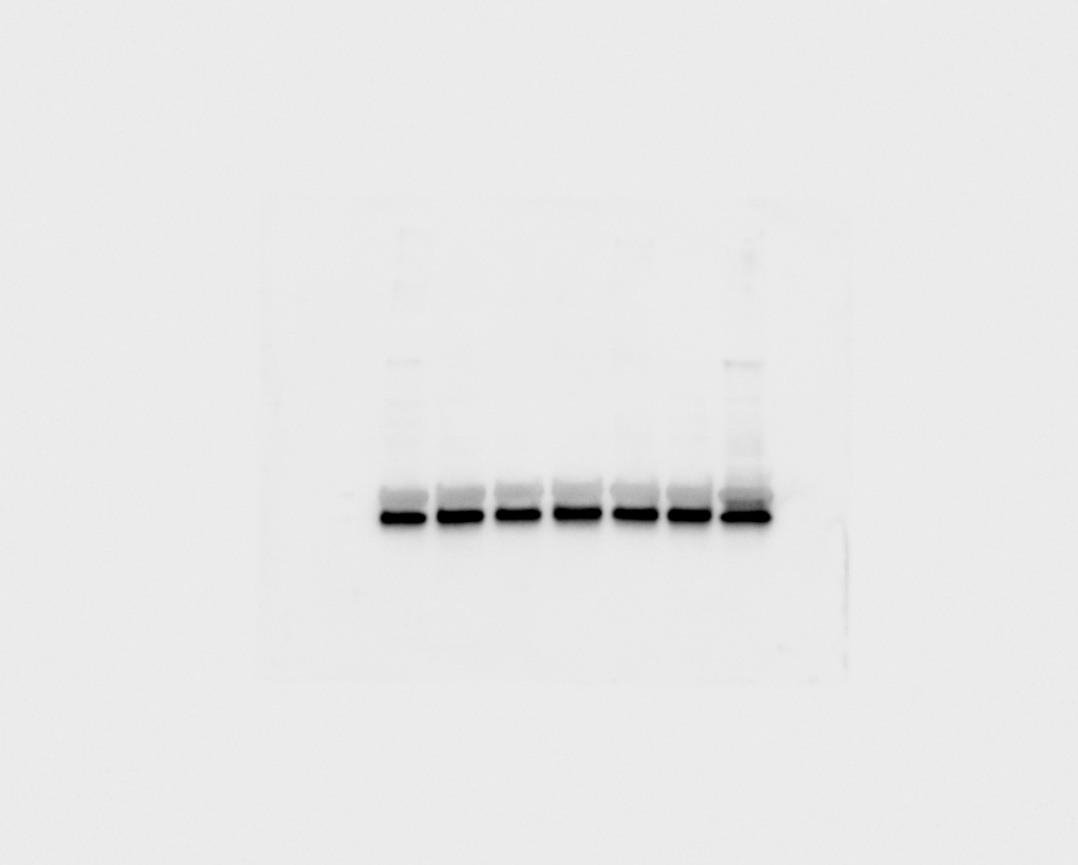


2C)

Gapdh (37Kda)

cGAS (67Kda)


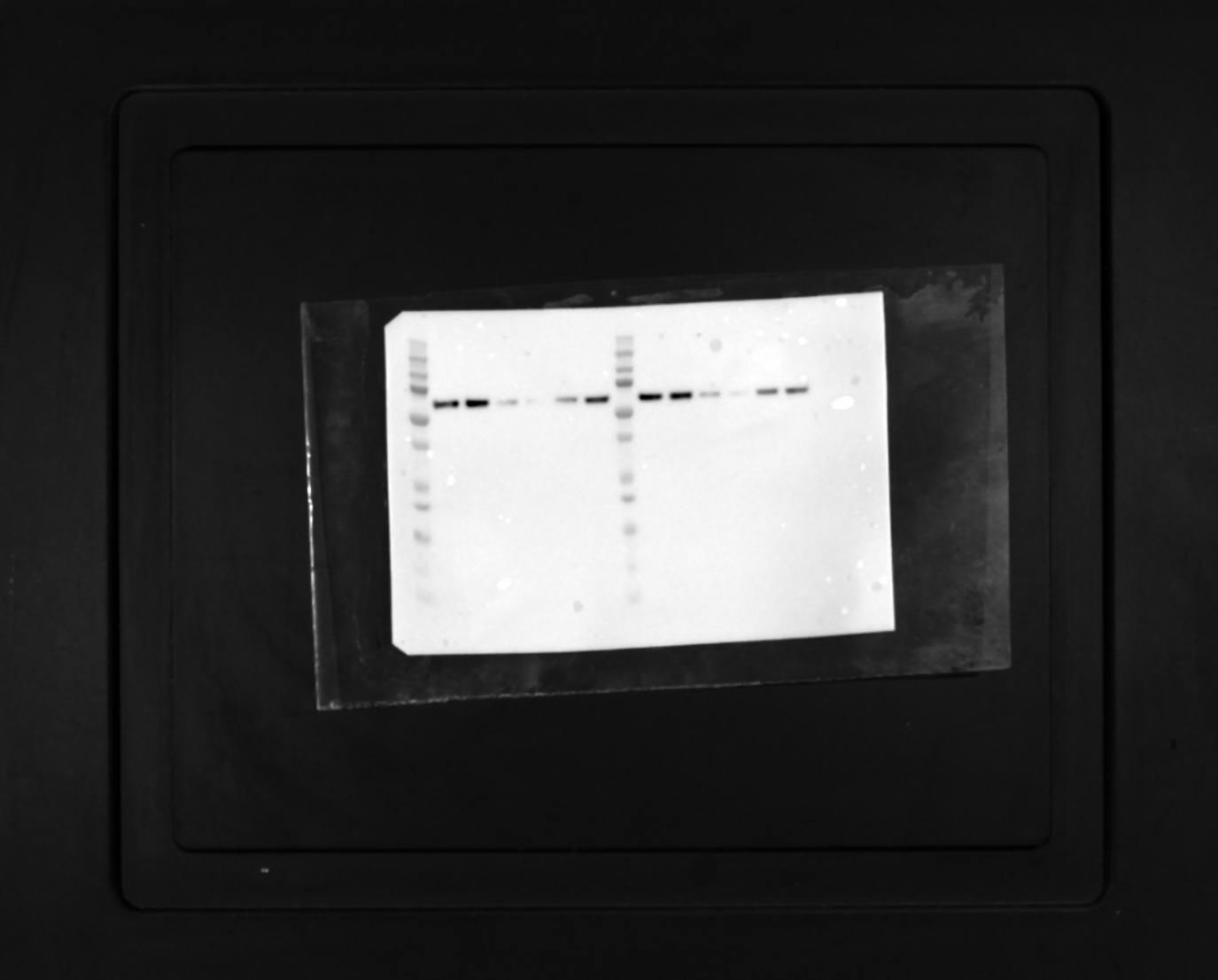

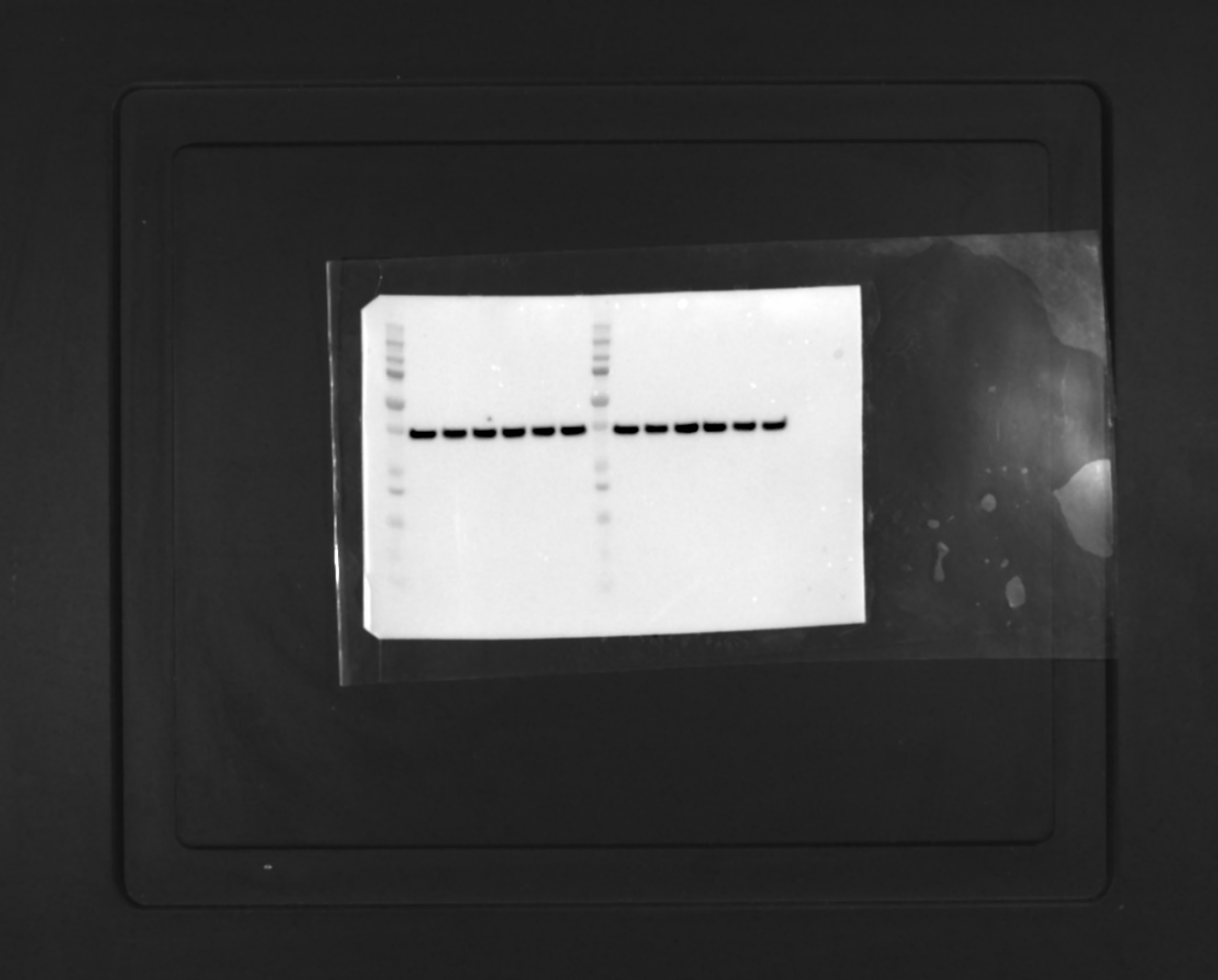


37Kda

50Kda

75Kda

50Kda

2D)

Gapdh (37Kda)

cGAS (67Kda)


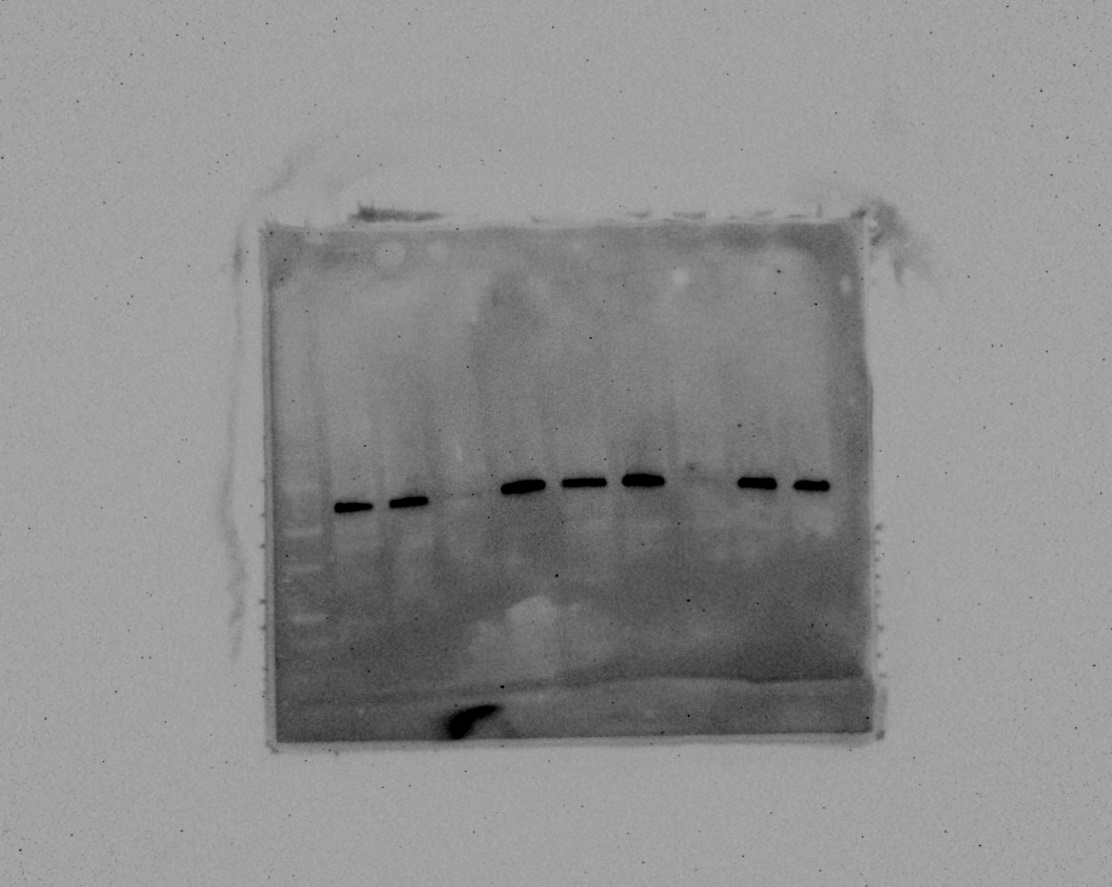

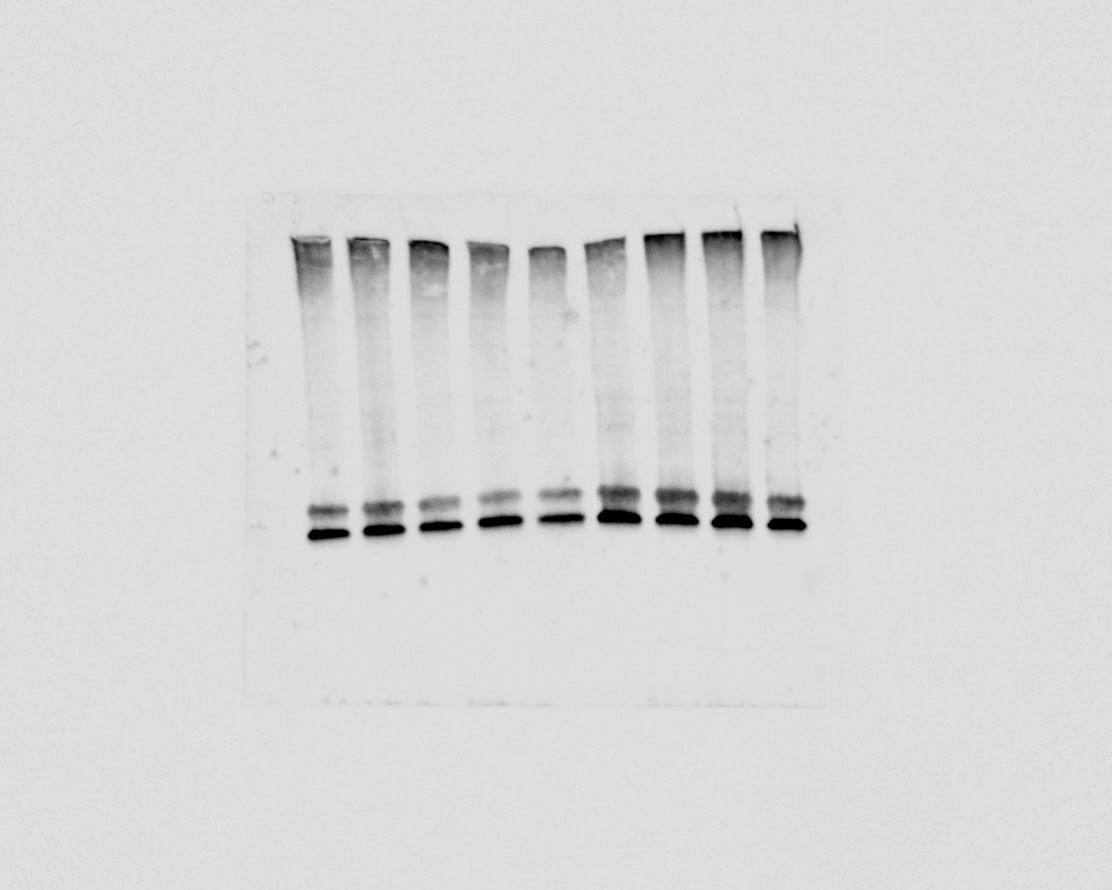


50Kda

75Kda

Figure 3

Actin (37Kda)

3A)


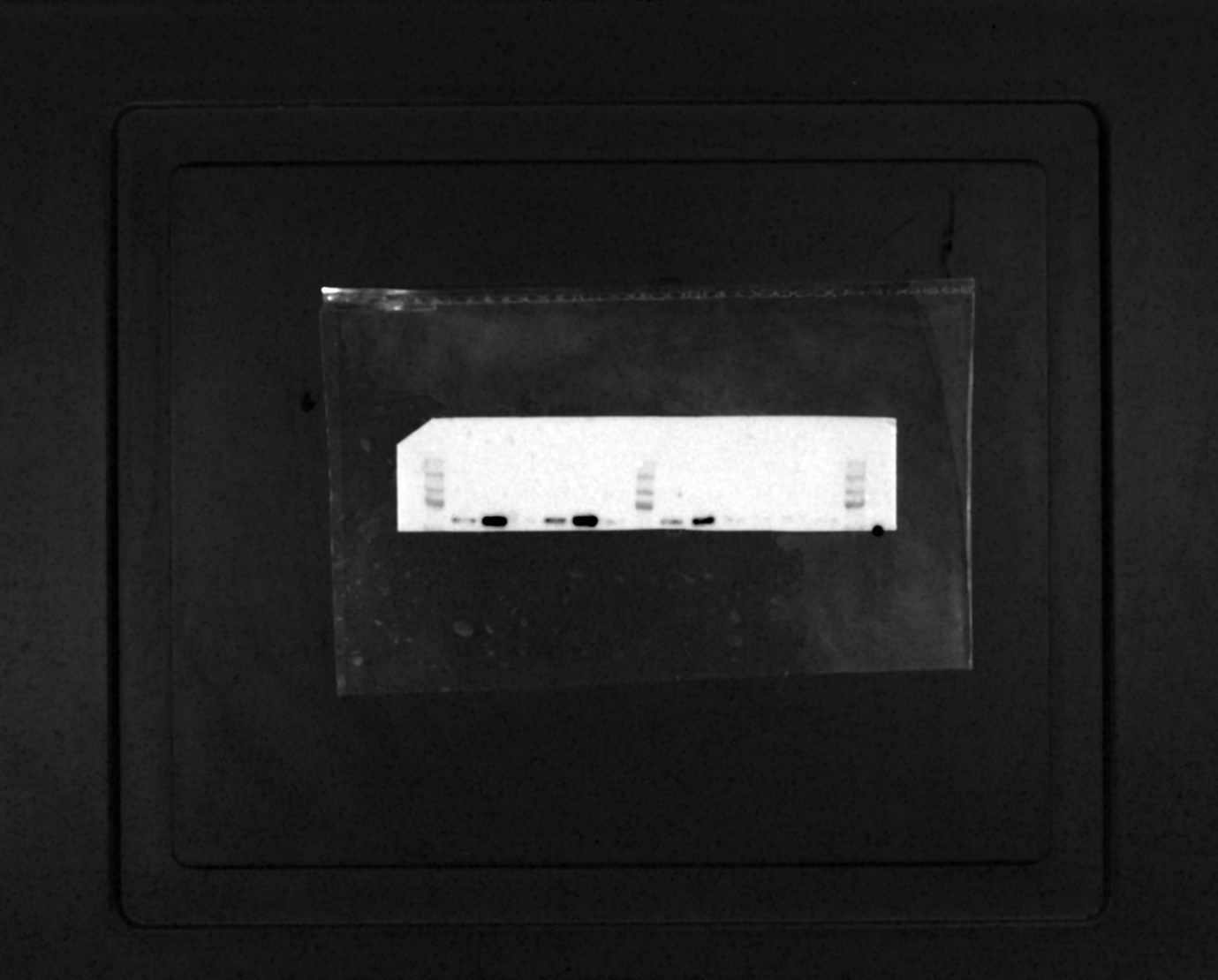

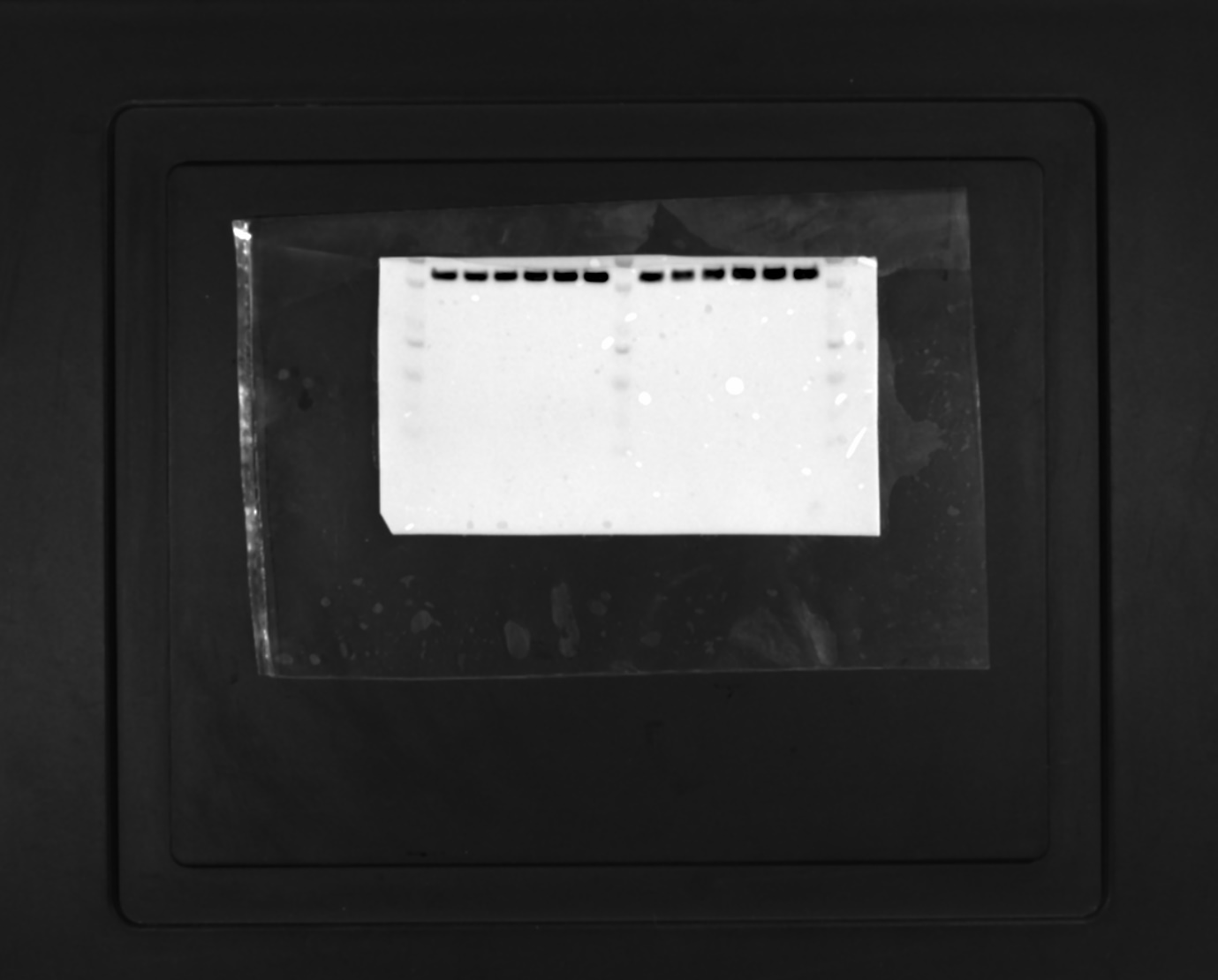


cGAS (67Kda)

75Kda

37Kda

3B)

Actin (37Kda)

cGAS (67Kda)


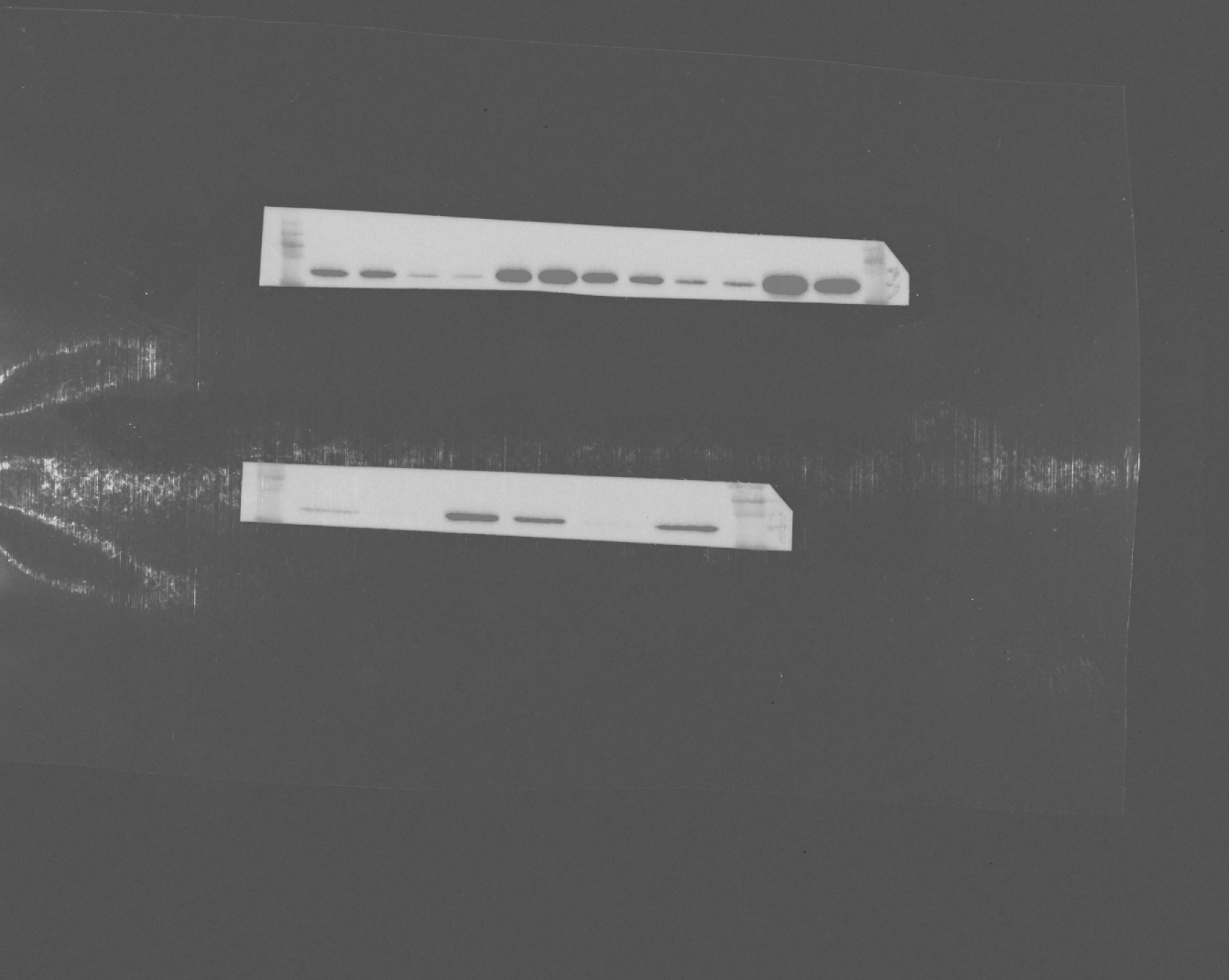

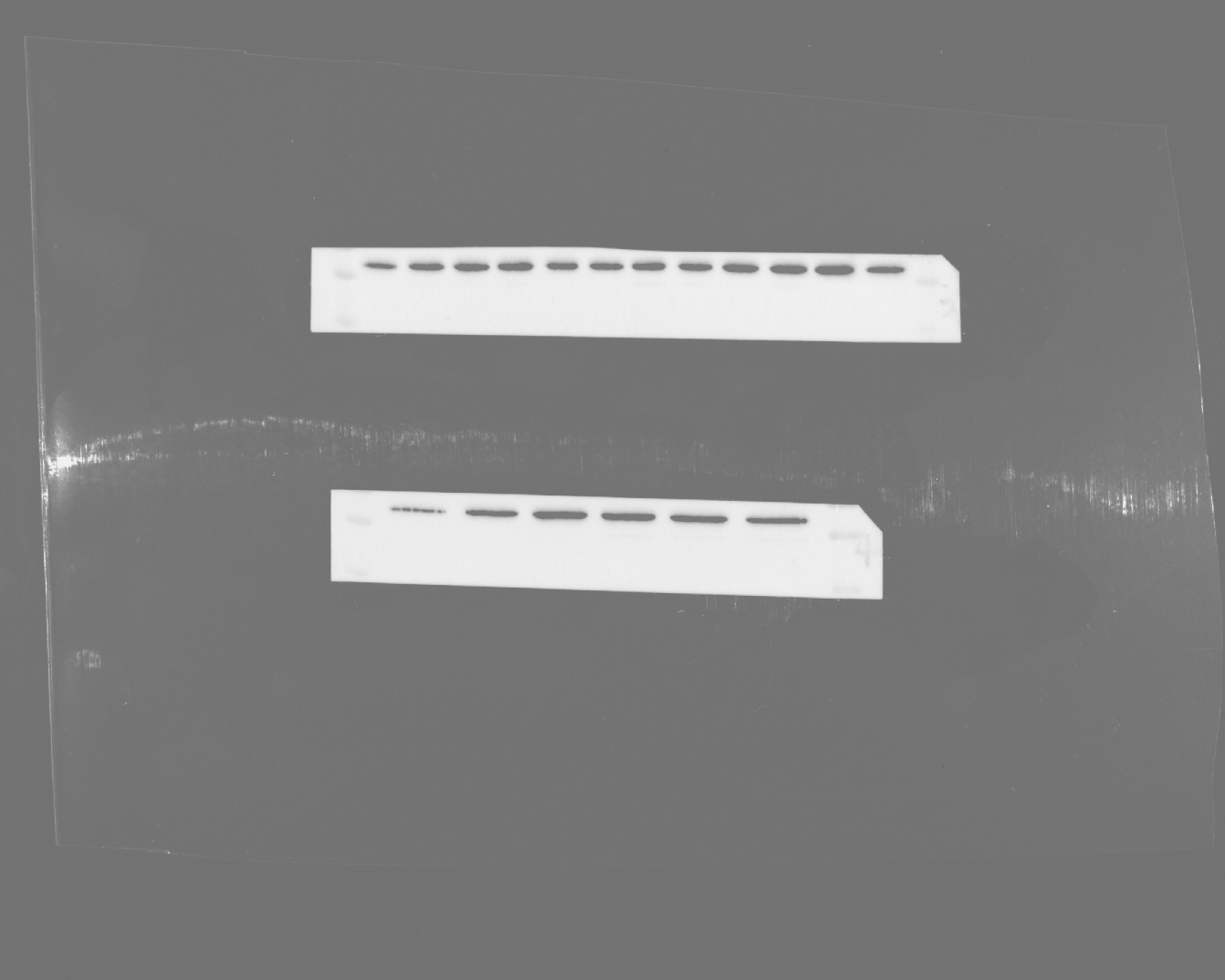


37Kda

50Kda

3C)

Actin (37Kda)

cGAS (67Kda)


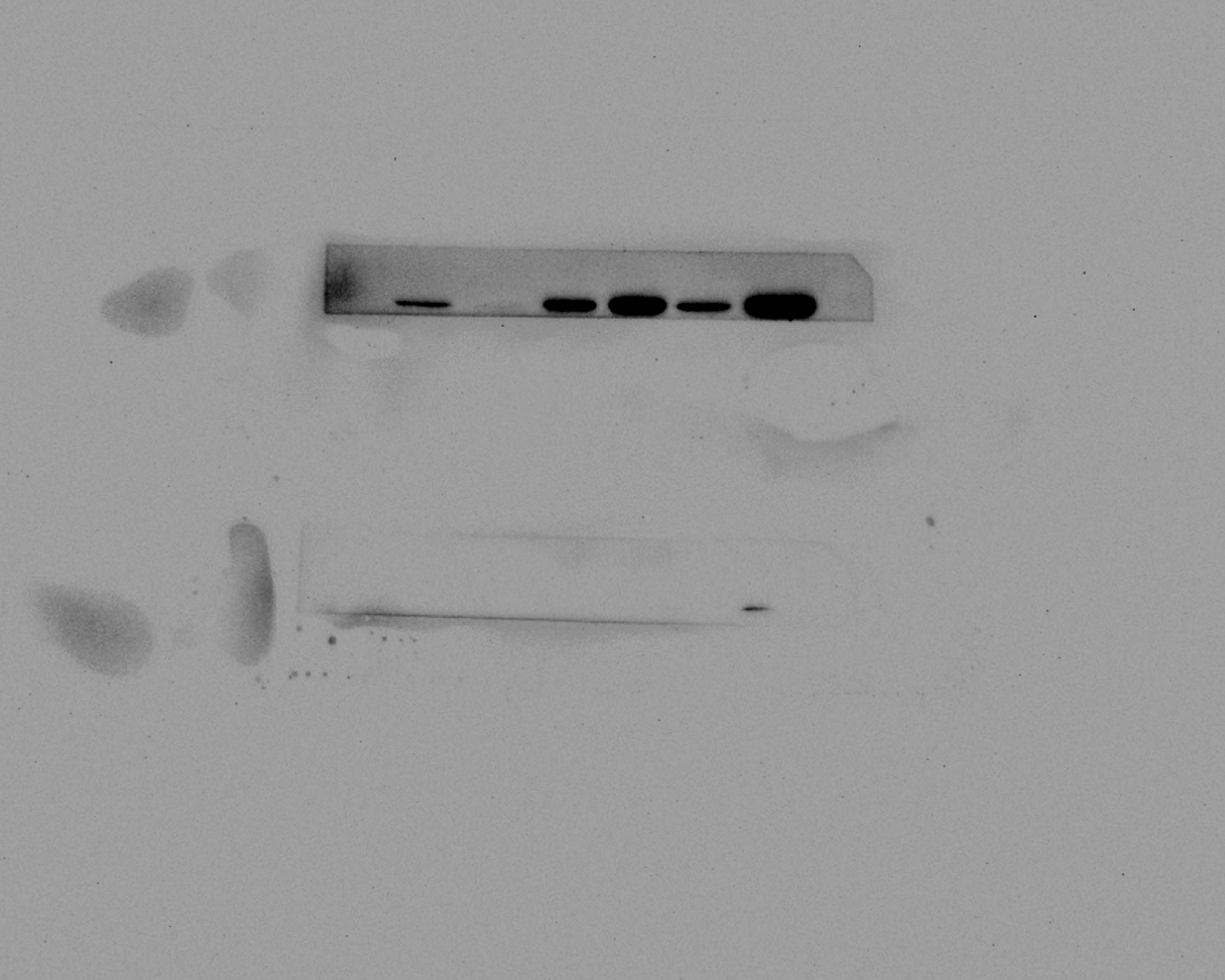

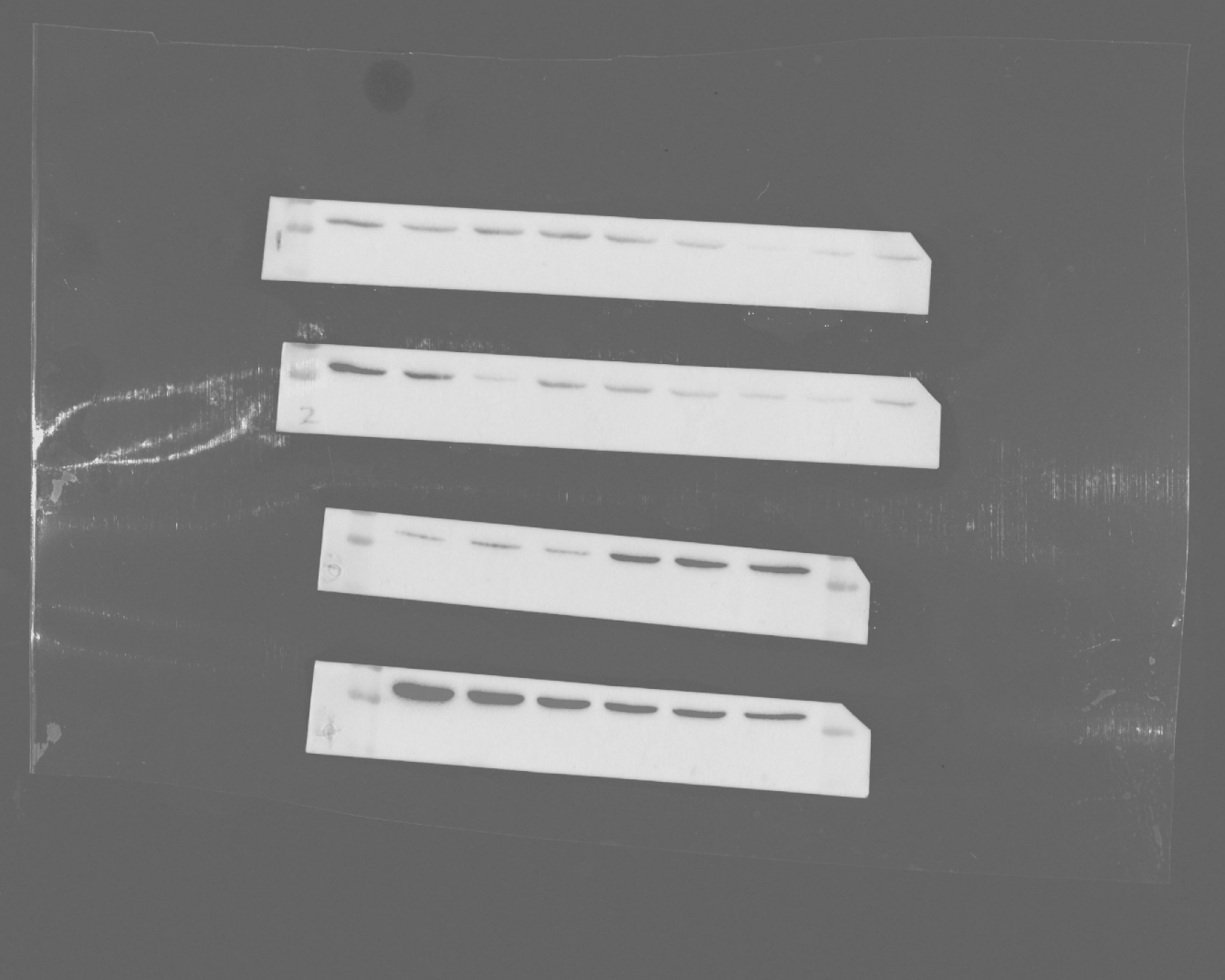


37Kda

3D&E

Gapdh (37Kda)

cGAS (67Kda)


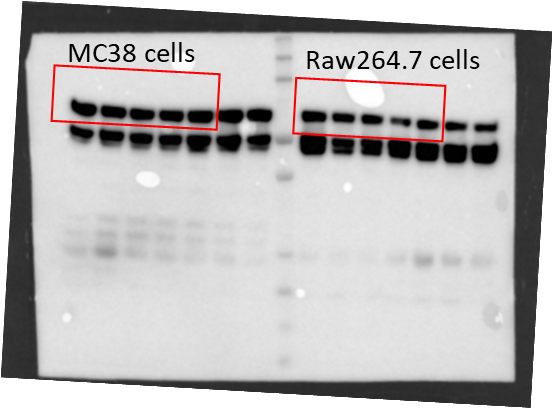

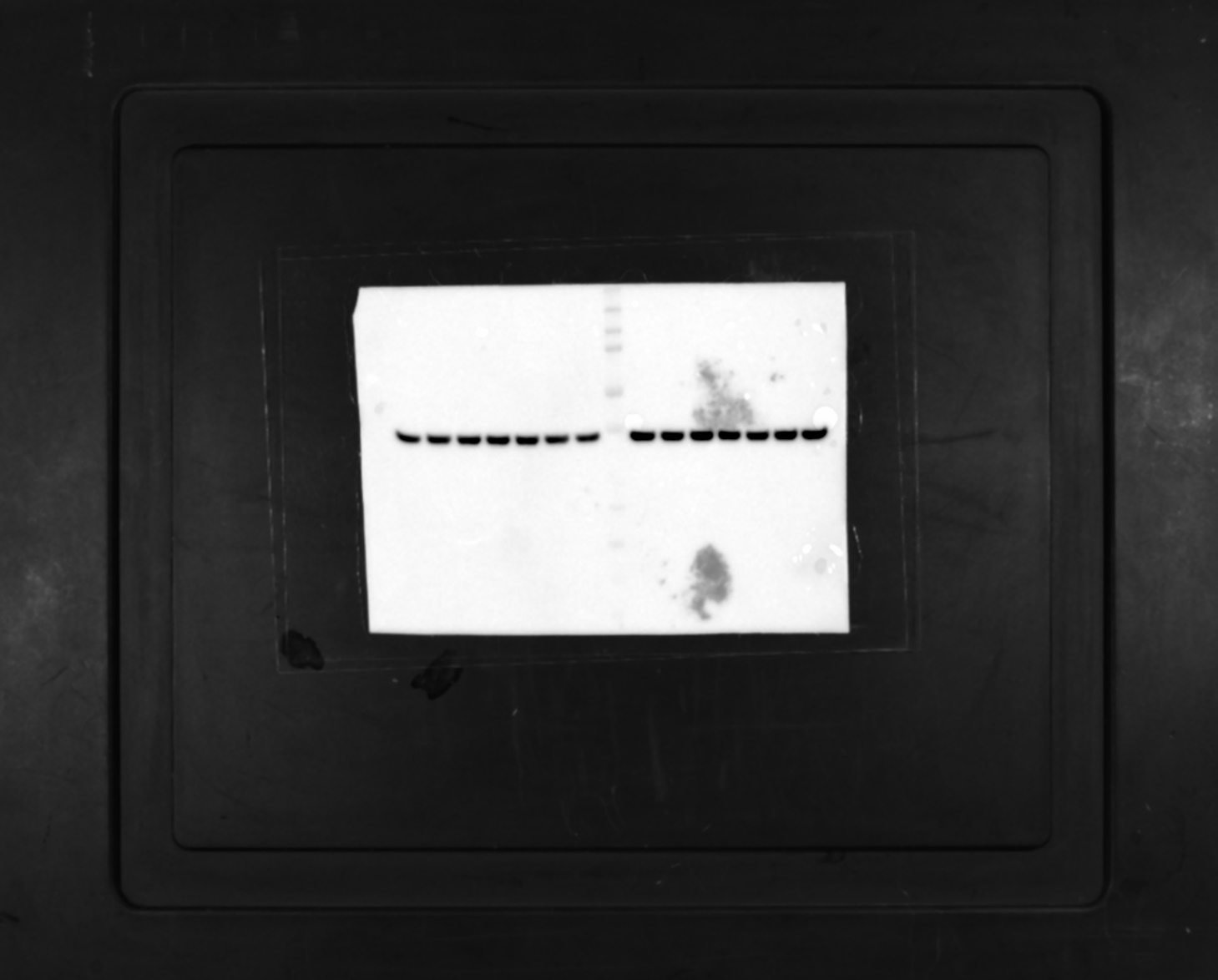


50Kda

37Kda

3F&G)


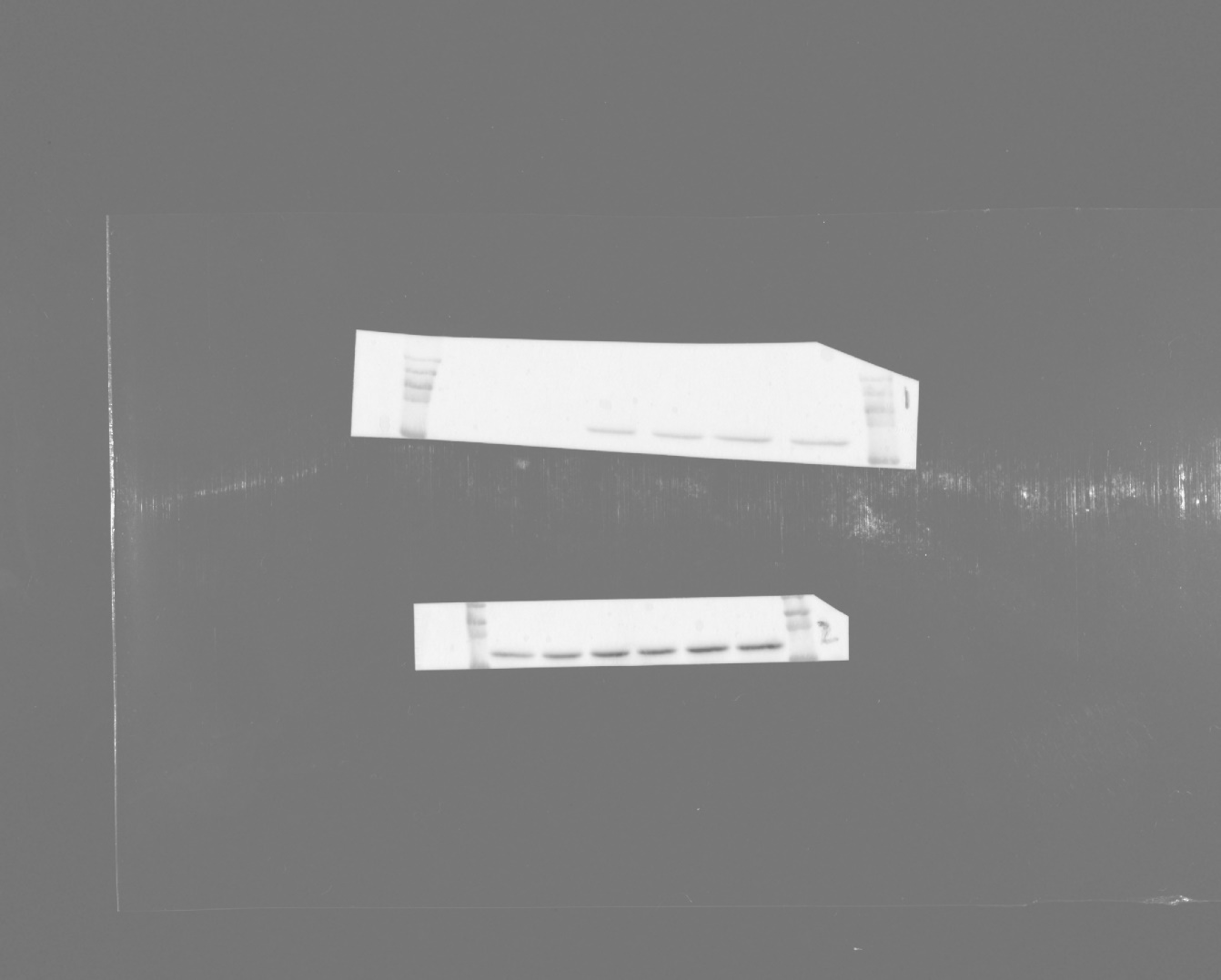

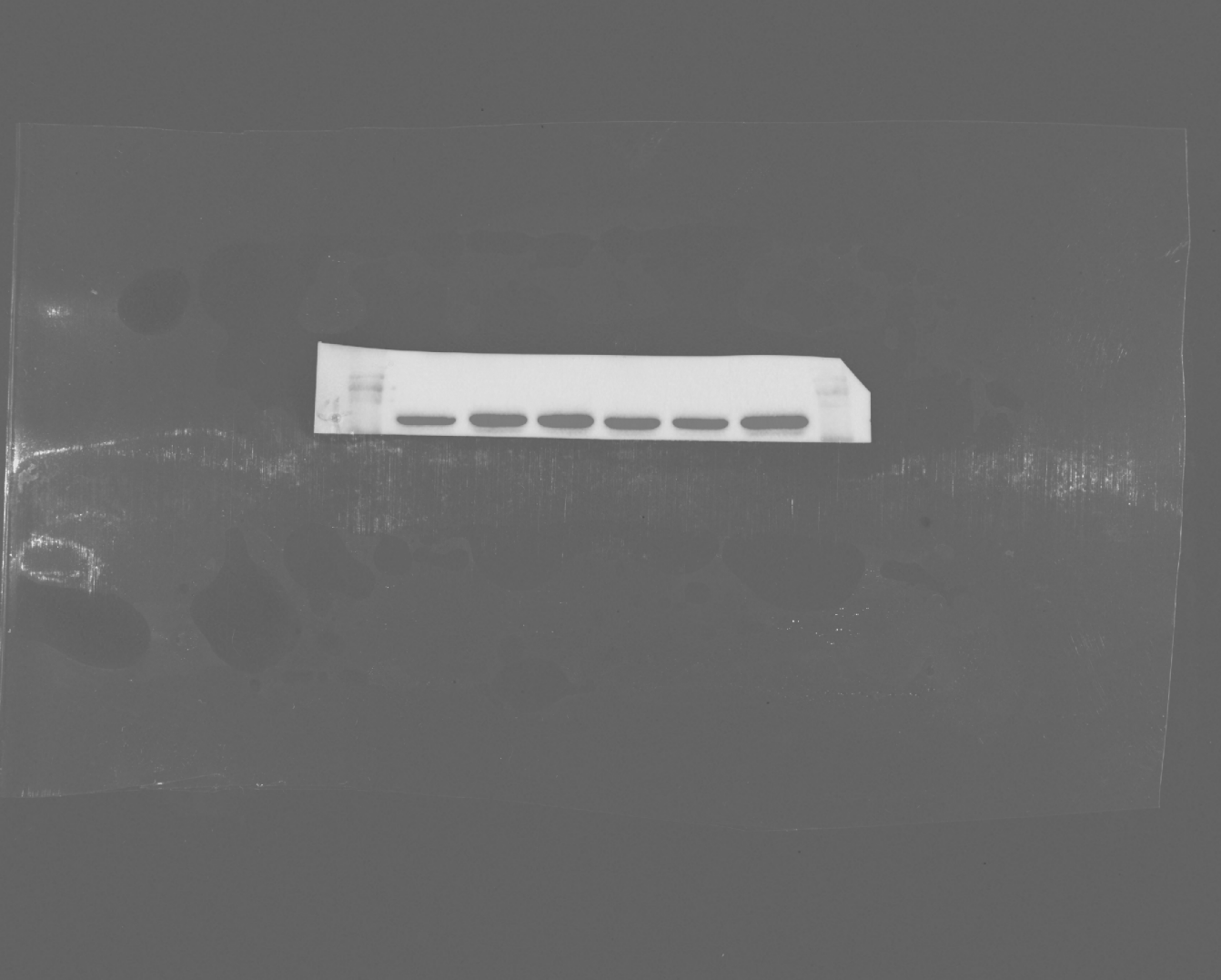


cGAS (67Kda)

cGAS (67Kda)

50Kda

50Kda


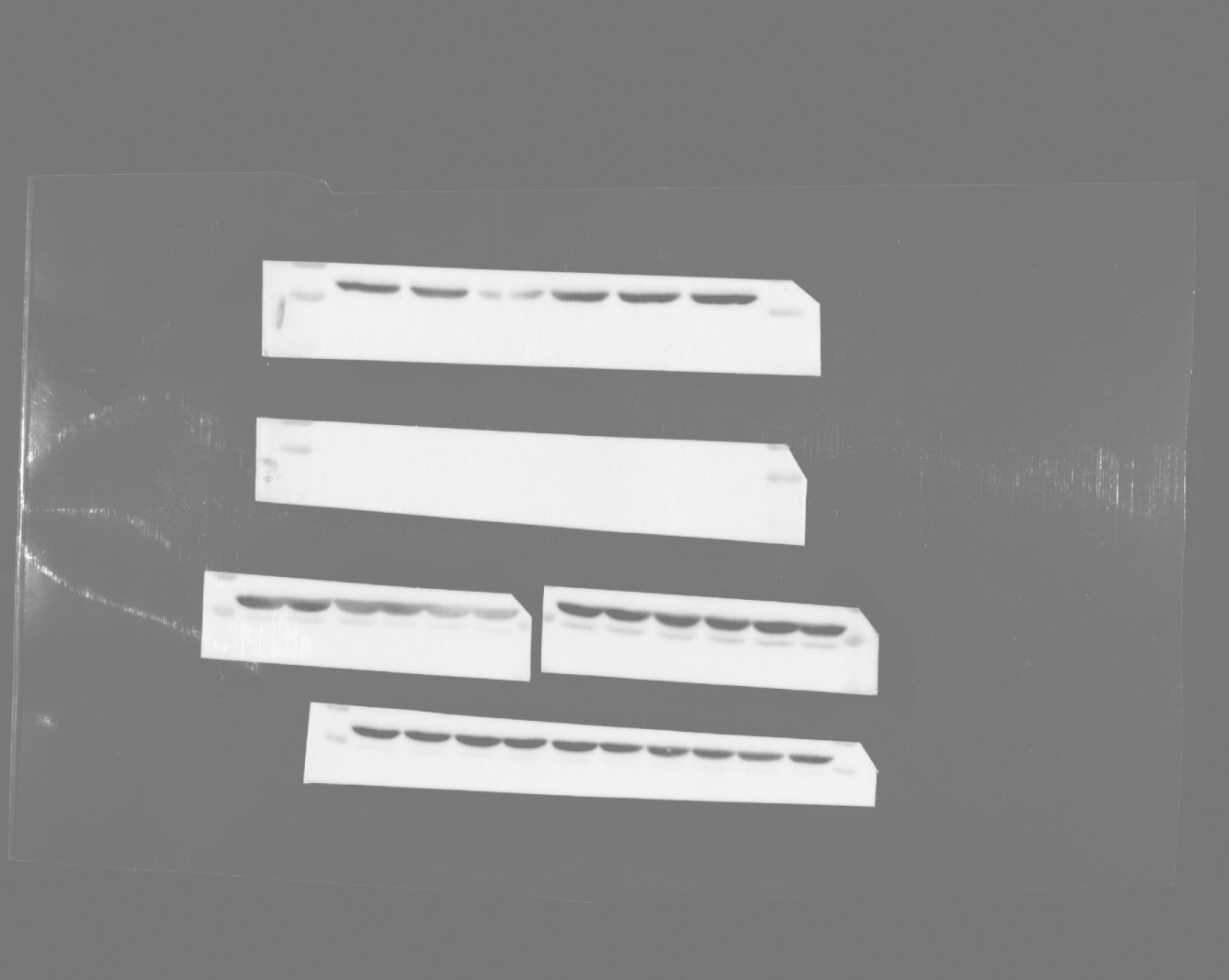

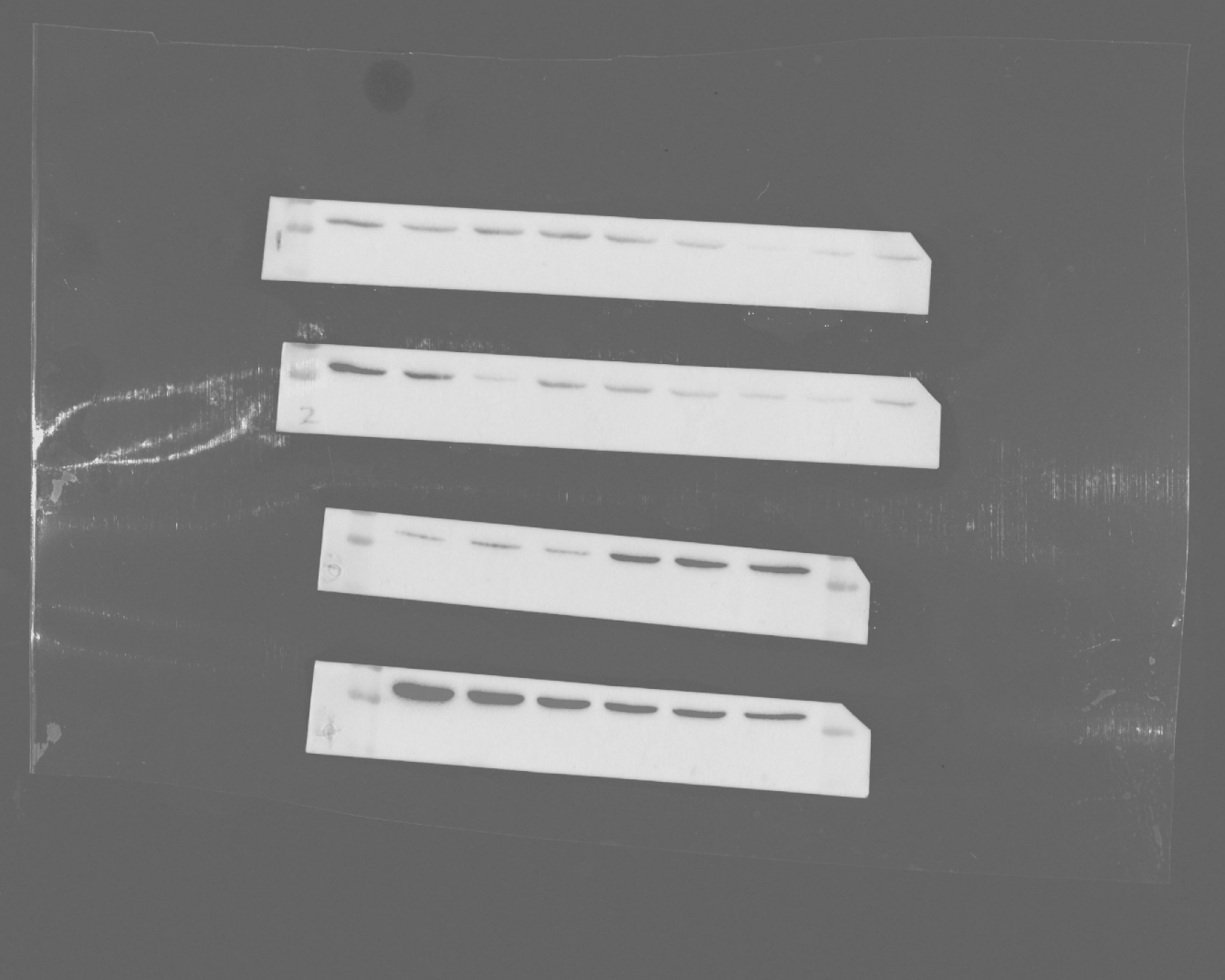


37Kda

Actin(42Kda)

Actin(42Kda)

37Kda

Figure4B)

cGAS (67Kda)


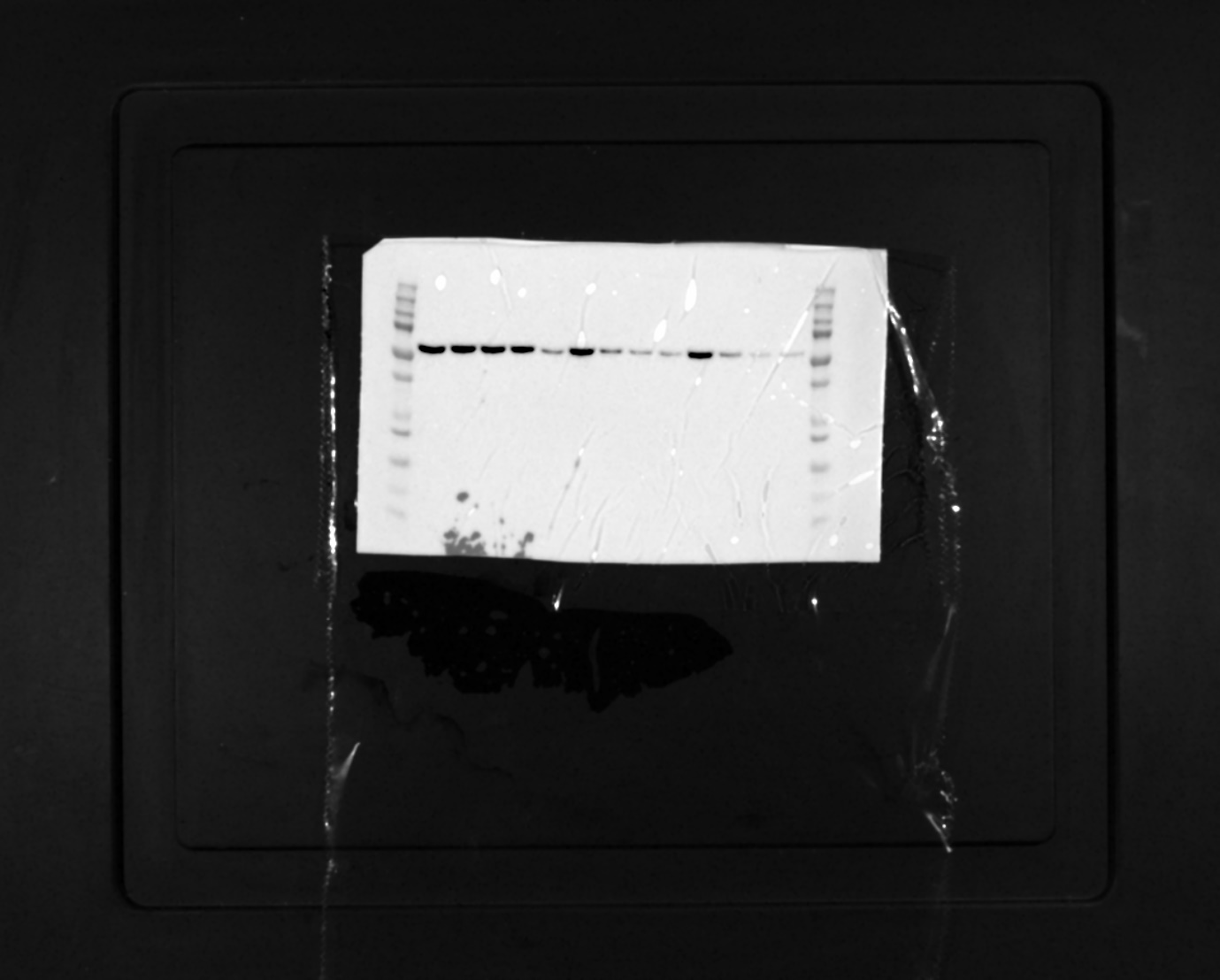

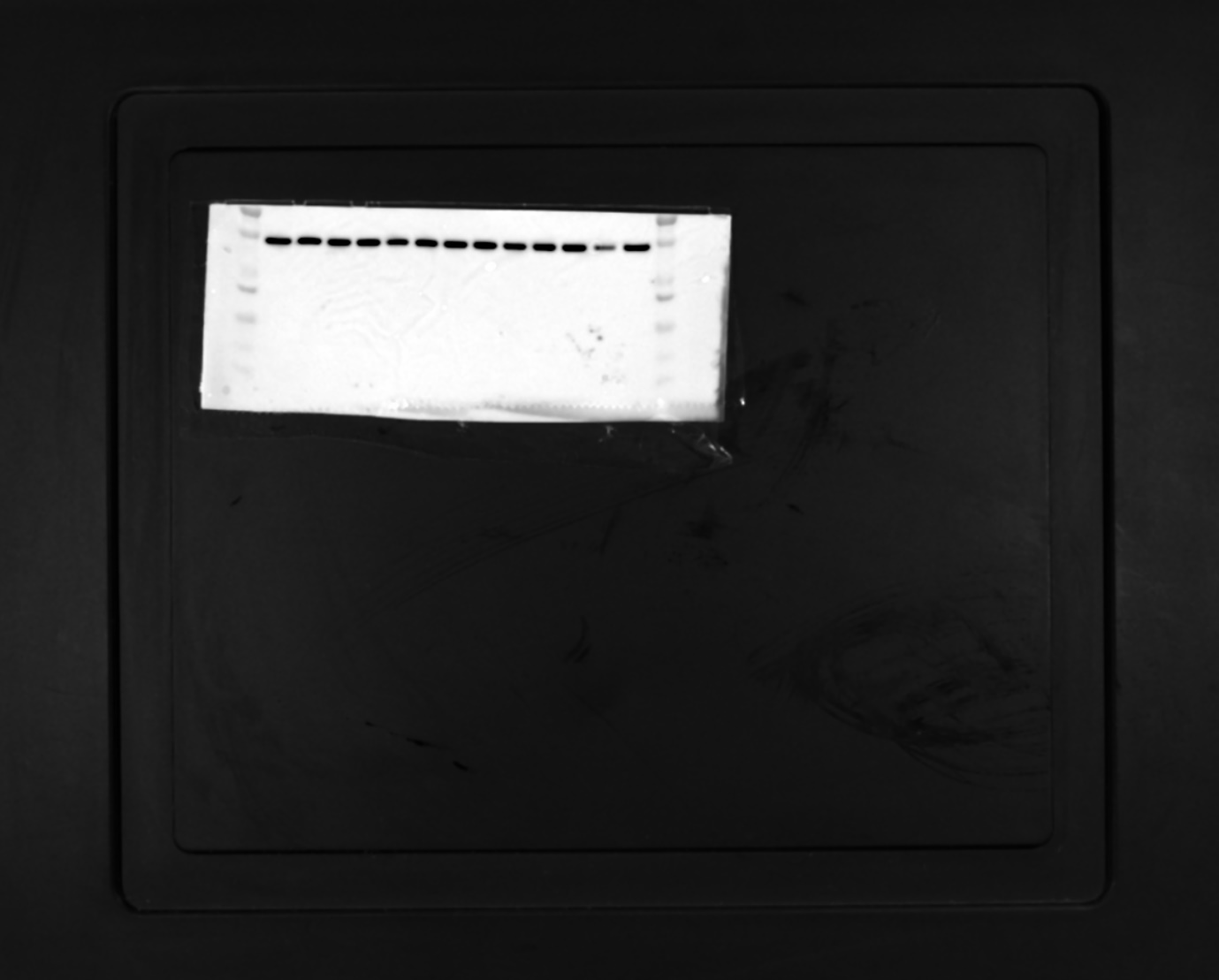


Gapdh (37Kda)

37Kda

50Kda

Figure 5)

5A)

cGAS (67Kda)


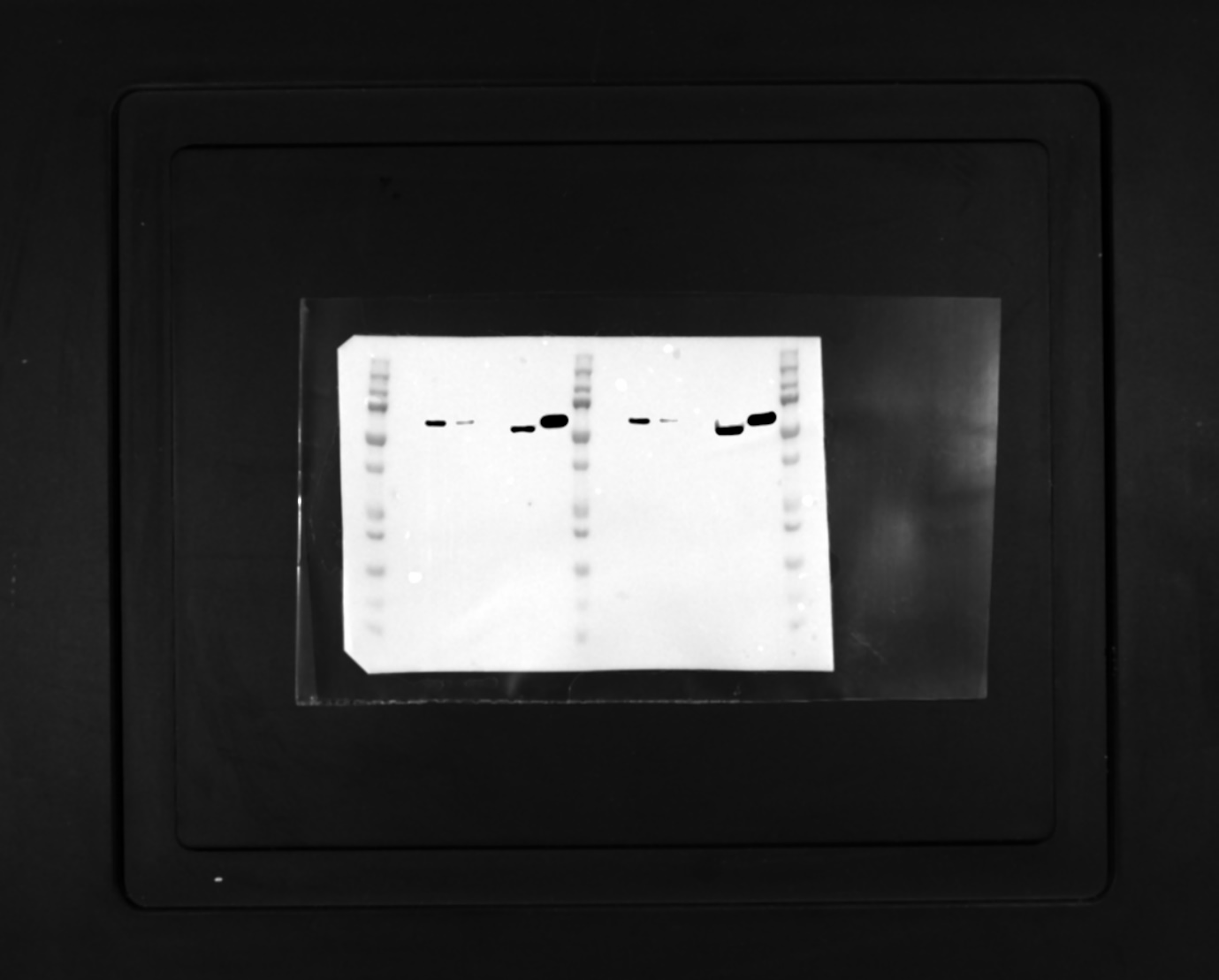


50Kda

5C)

cGAS (67Kda)

Ubiquitin


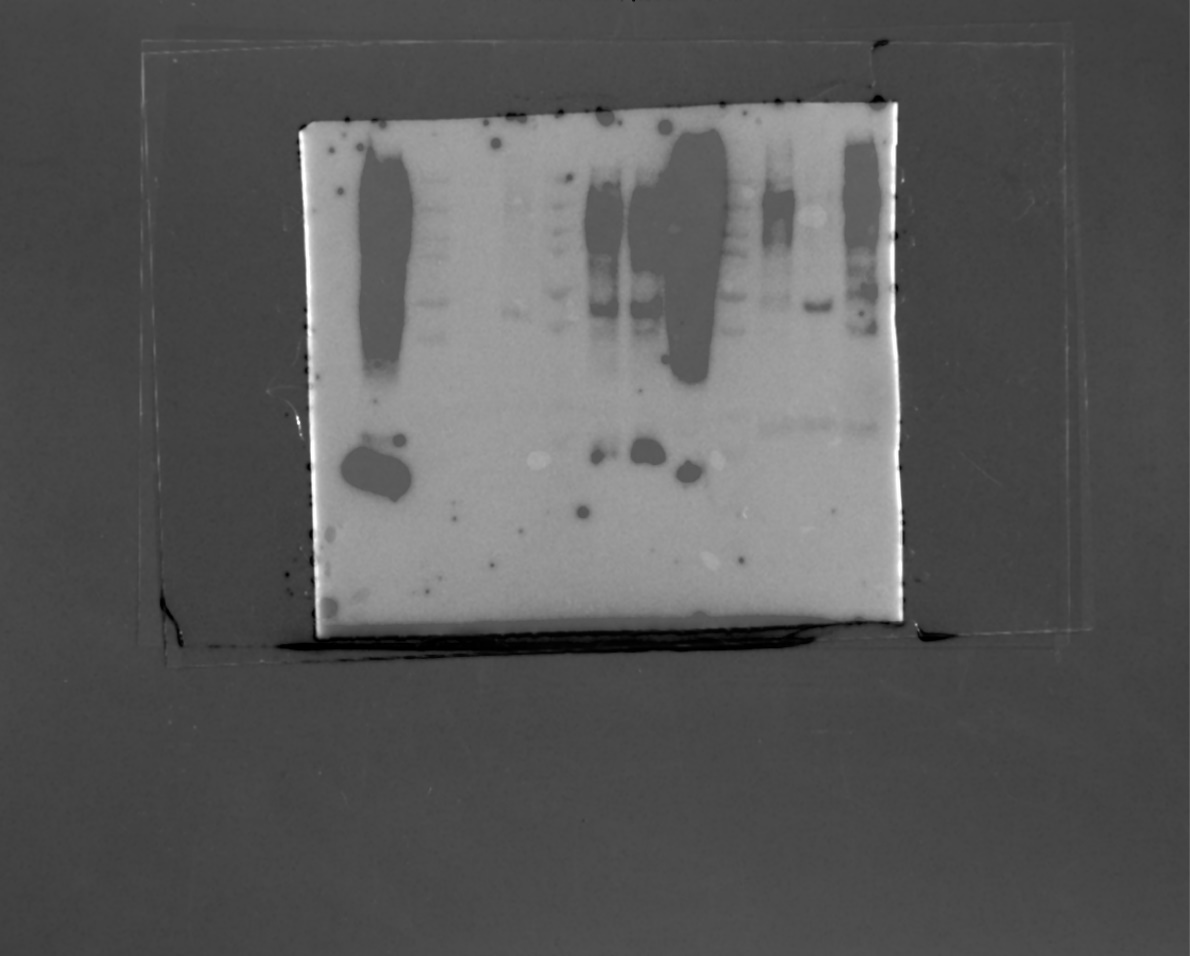

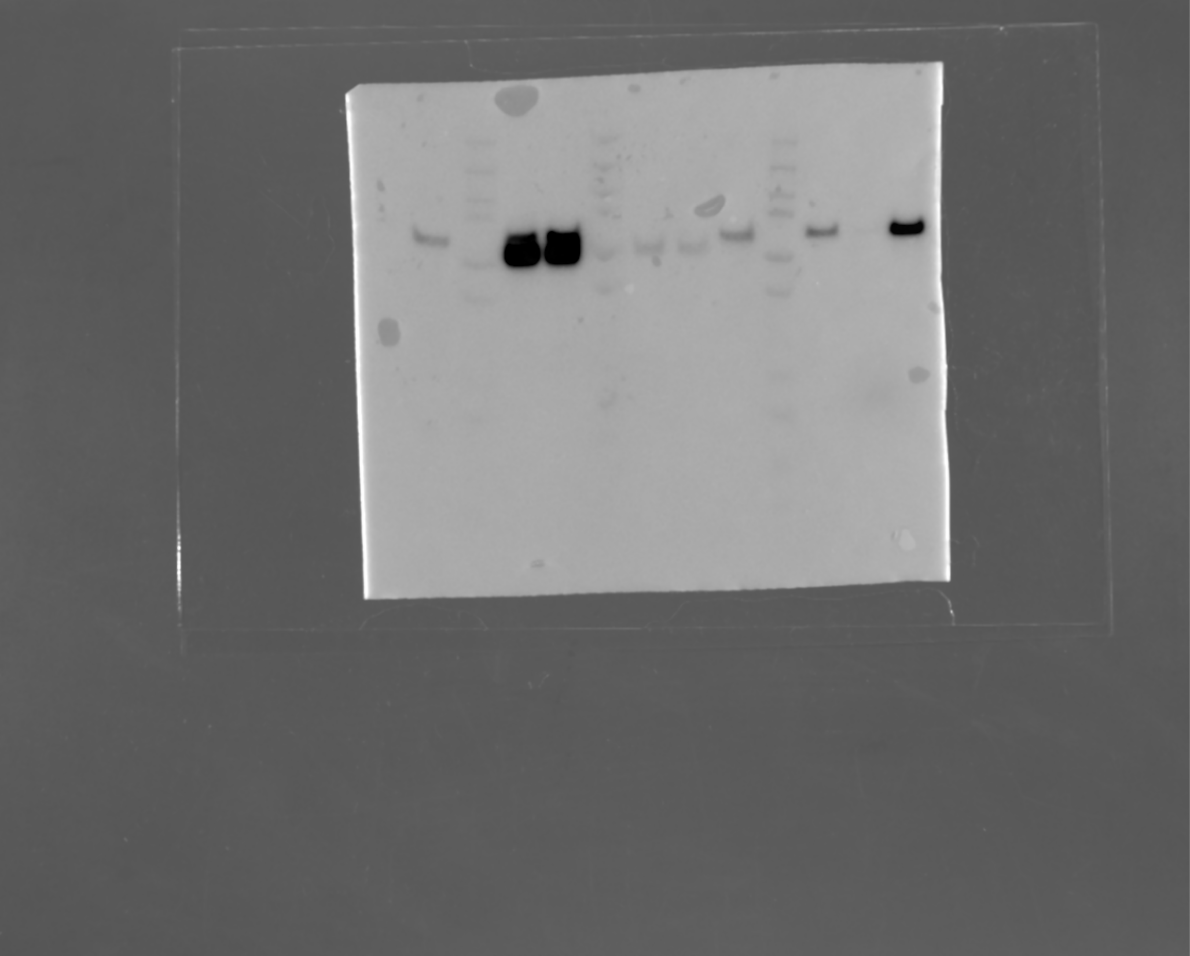


50da

50Kda

50Kda

50da

5D)

Gapdh (37Kda)

cGAS (67Kda)


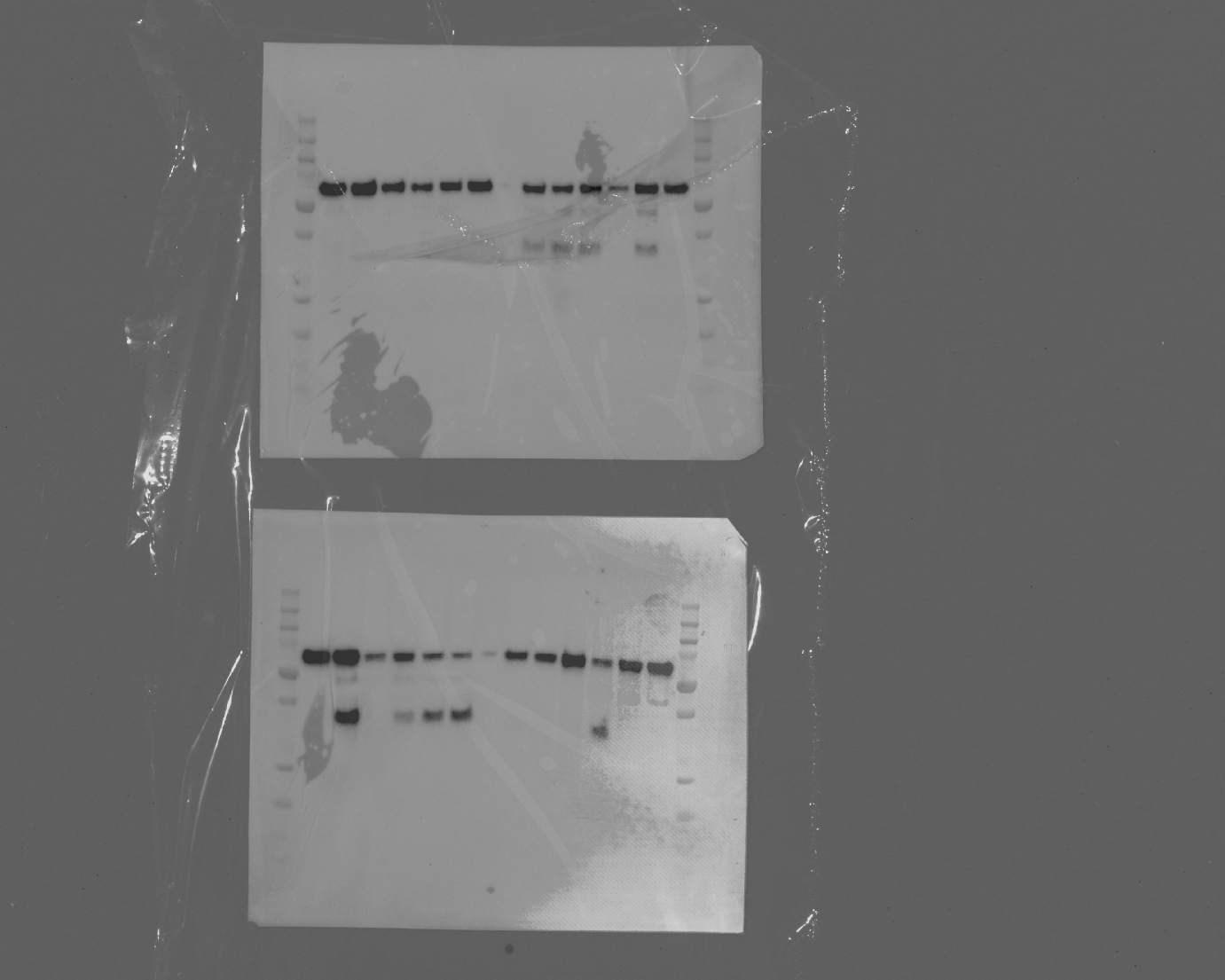

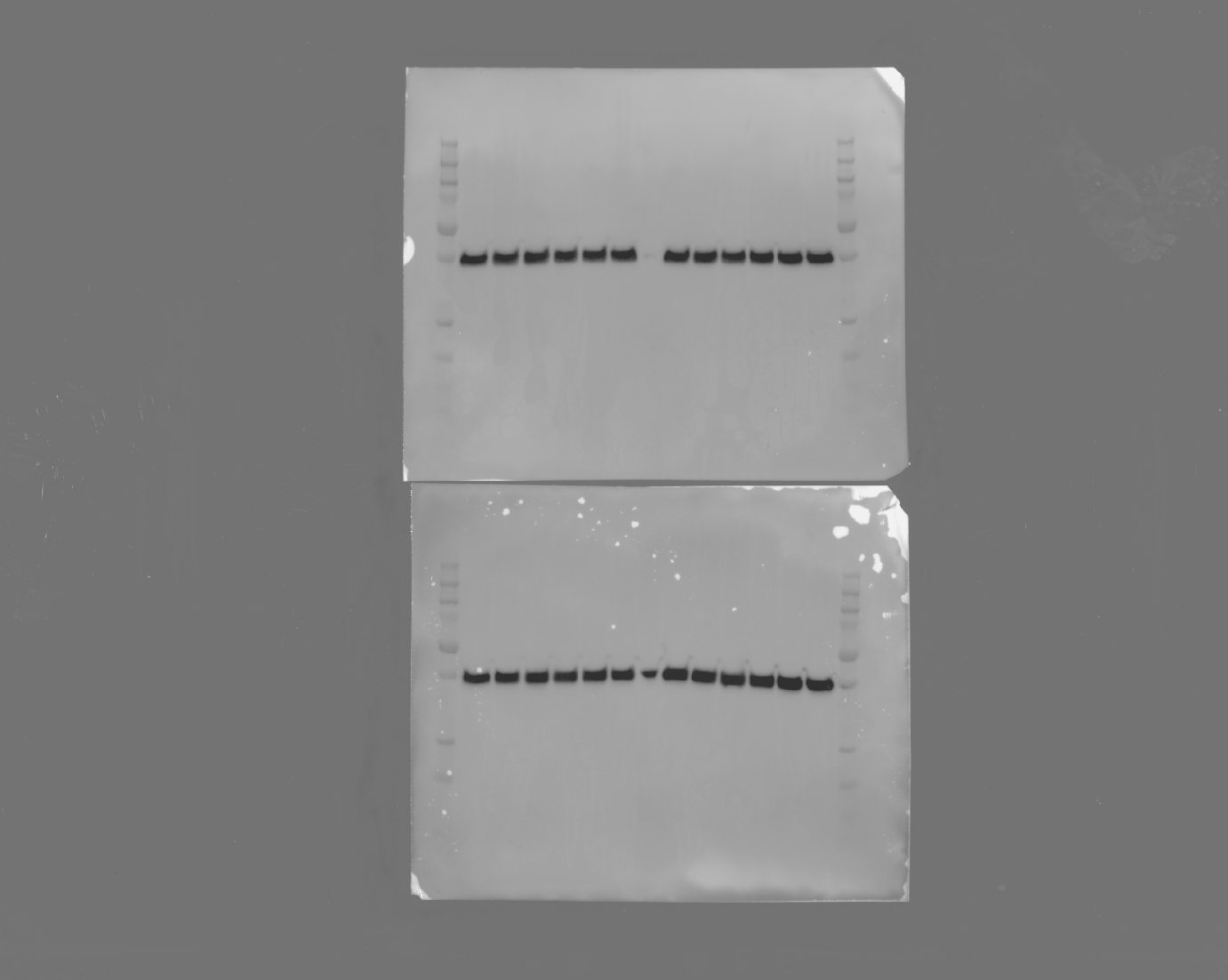


75Kda

50Kda

37Kda

5E)

Znf599Kda


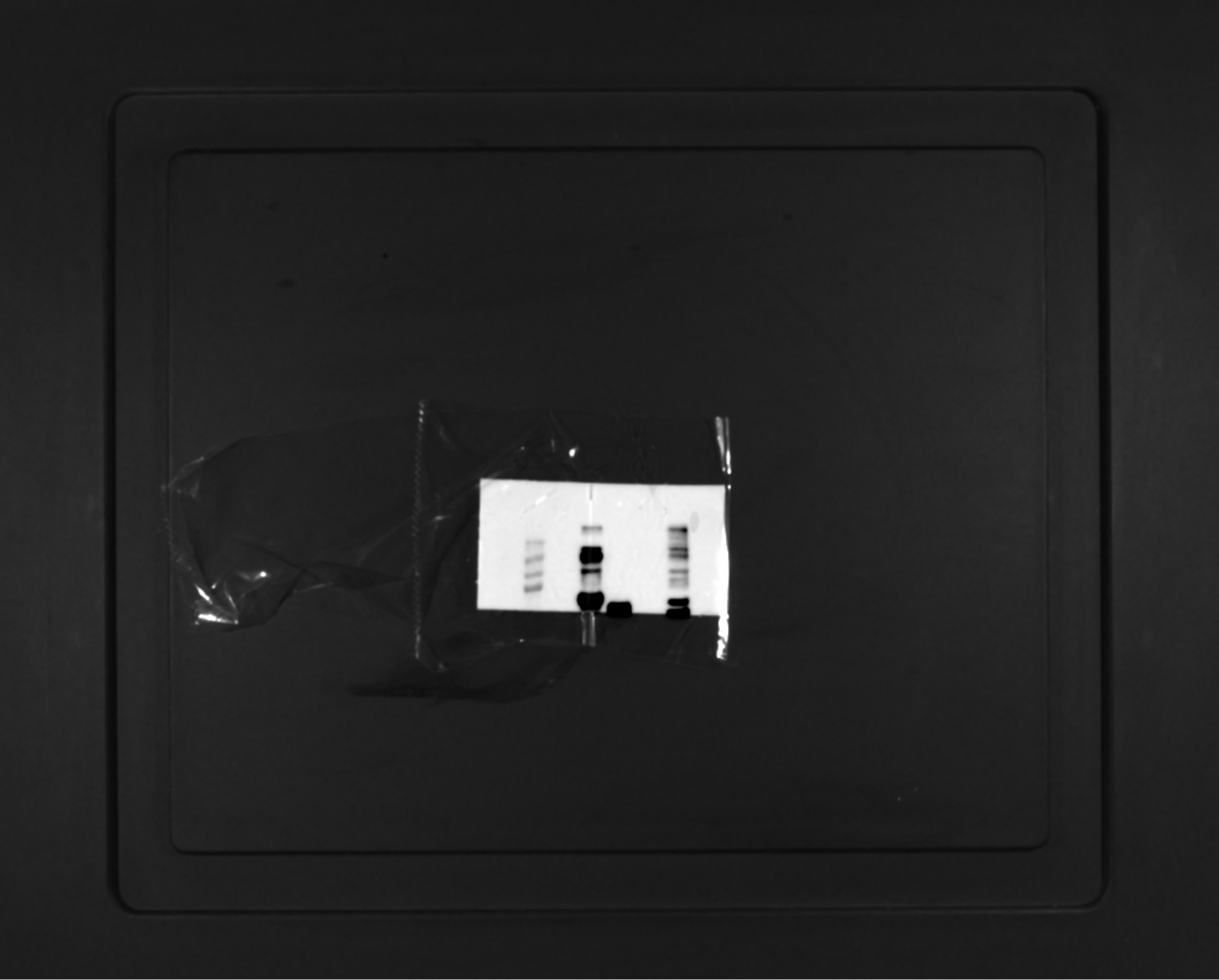


100Kda

5F)

cGAS (67Kda)

50Kda


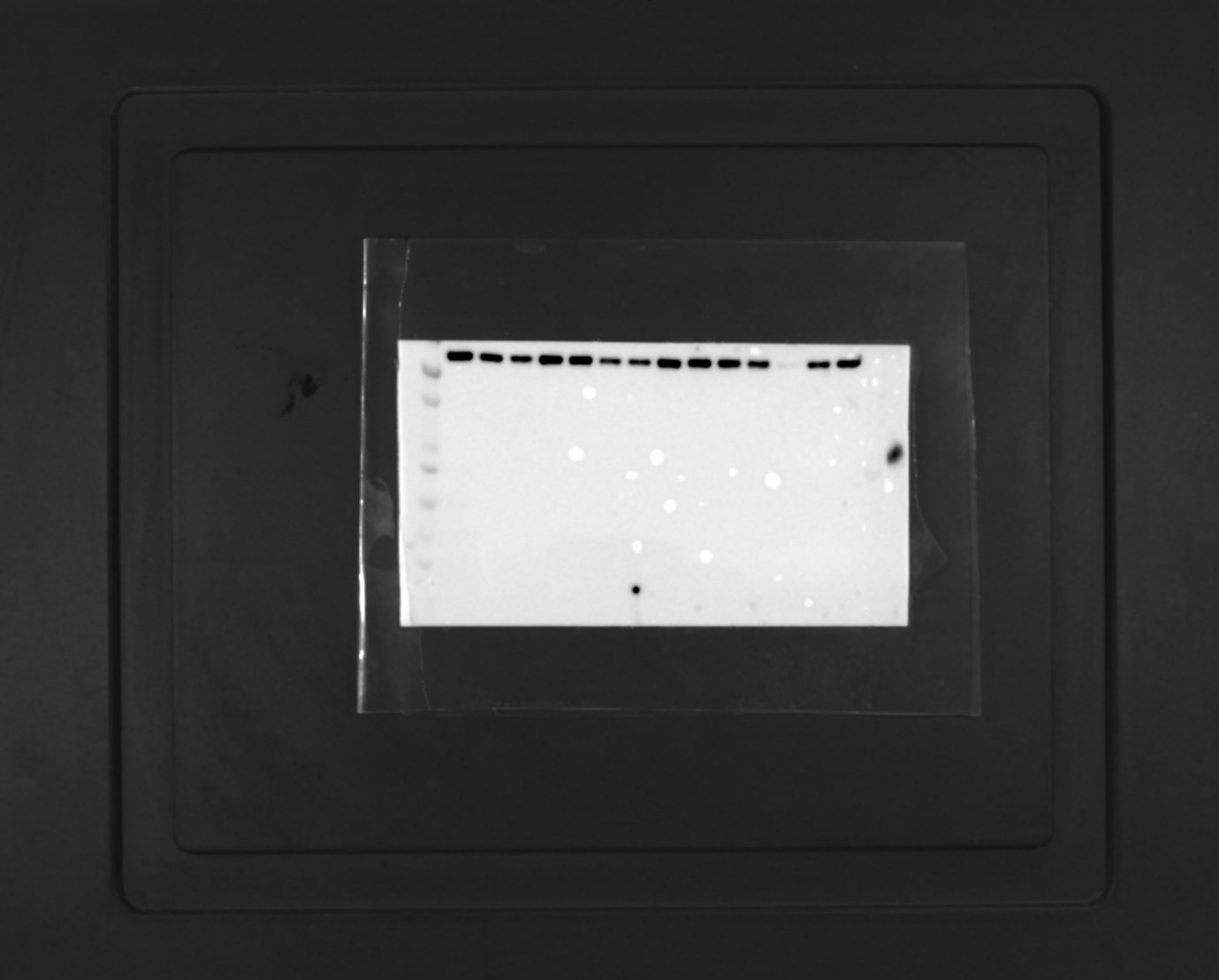


Gapdh (37Kda)

Znf599Kda

37Kda

100Kda


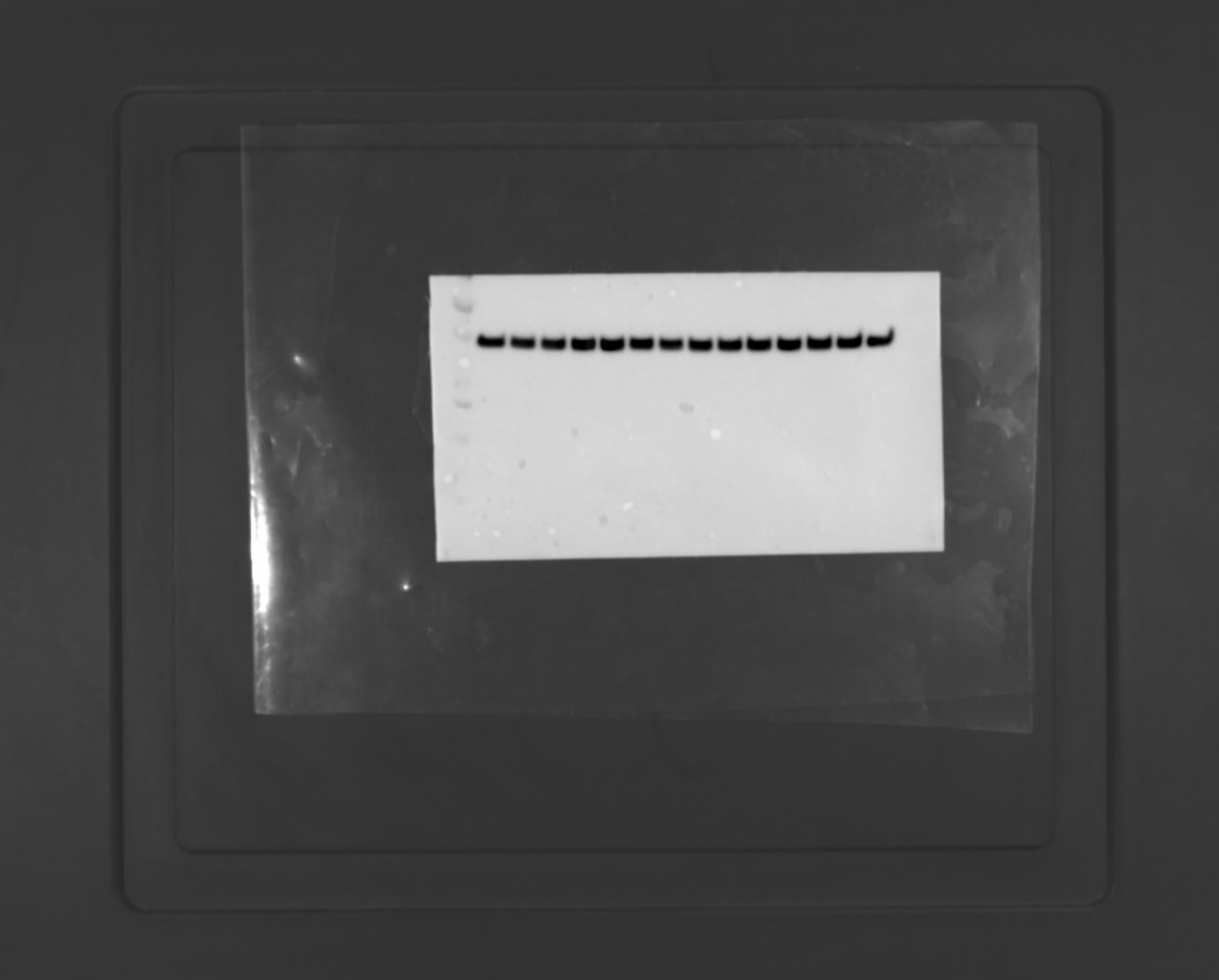

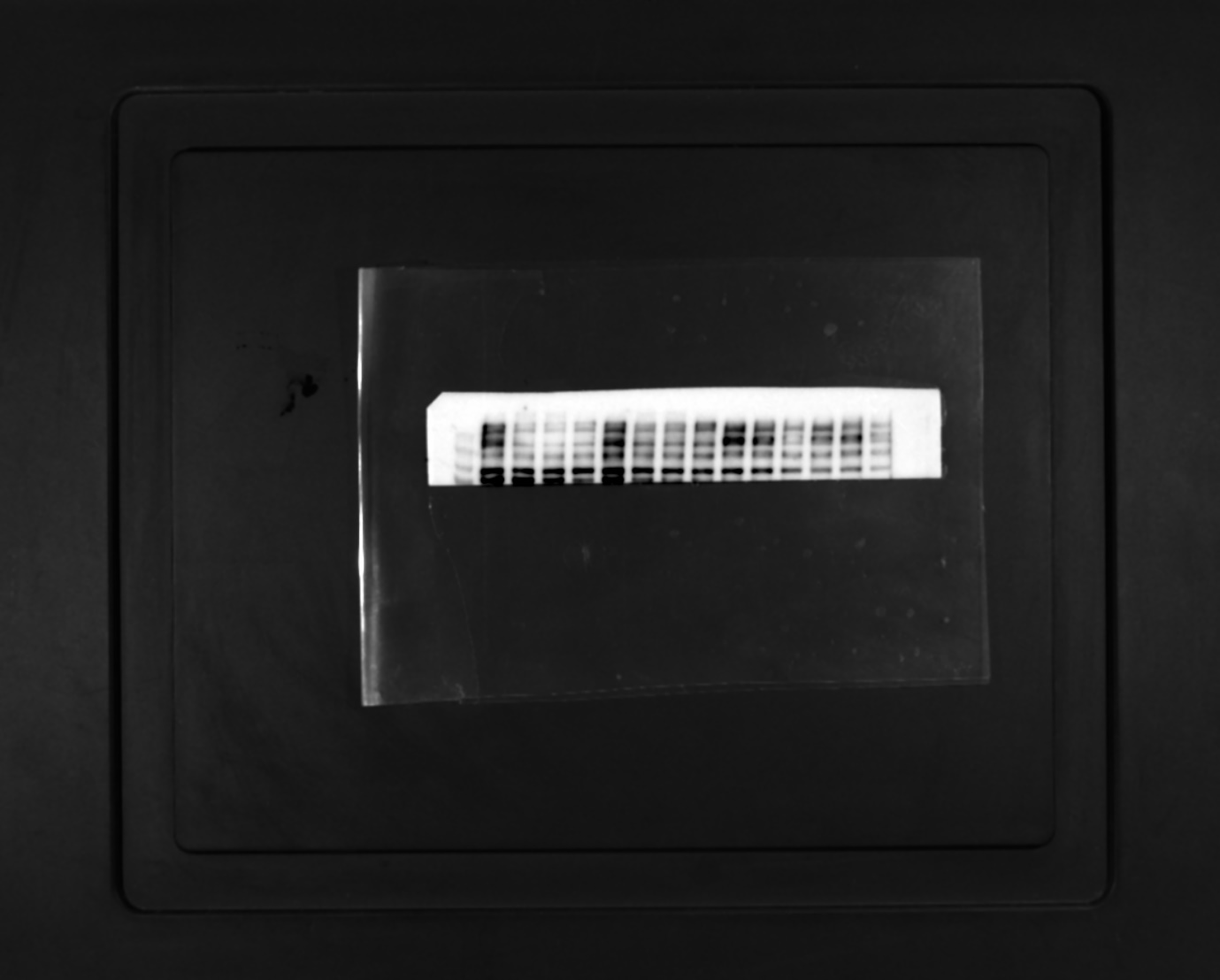


cGAS (67Kda)

Figure6A)

Pan-actin (45Kda)


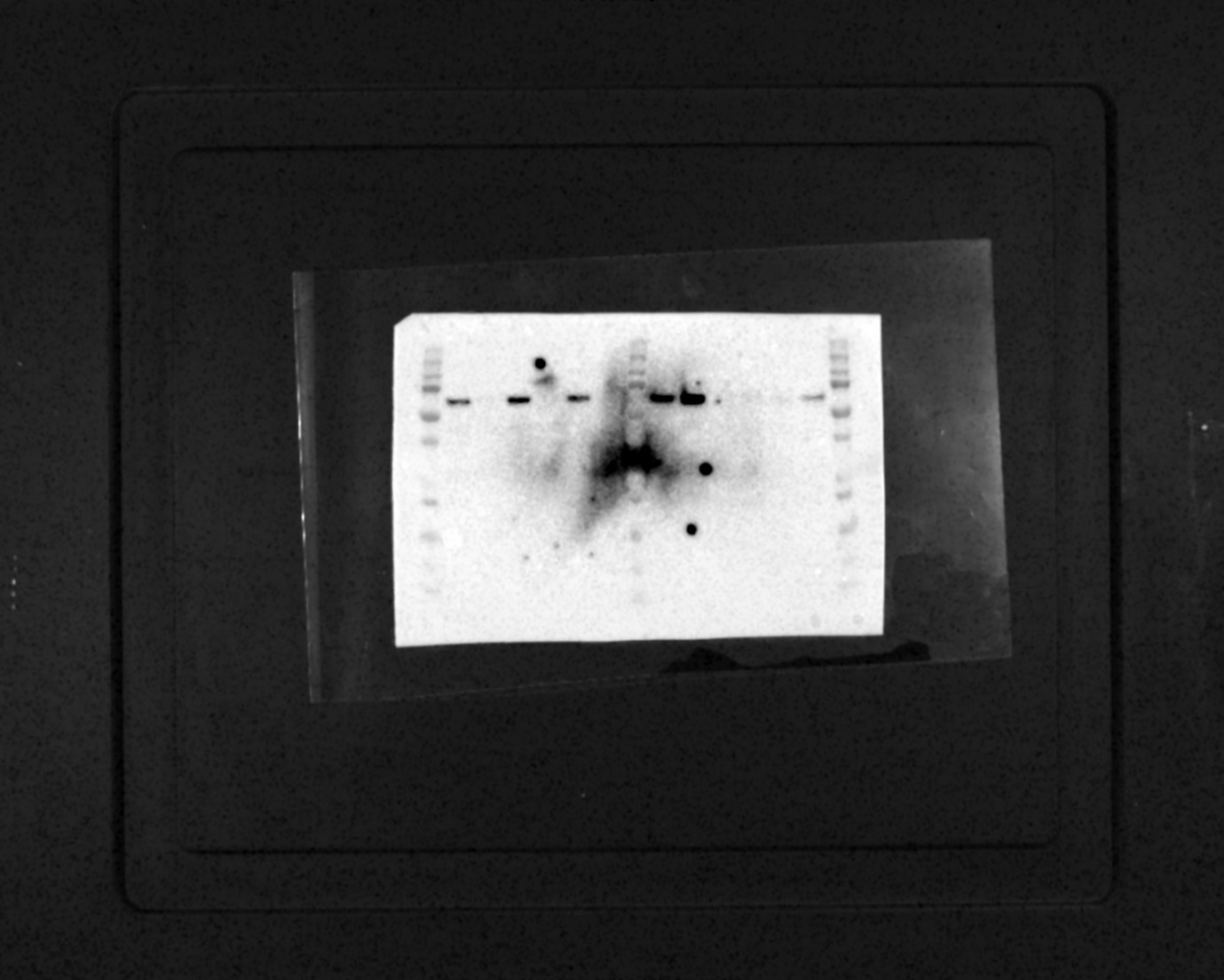

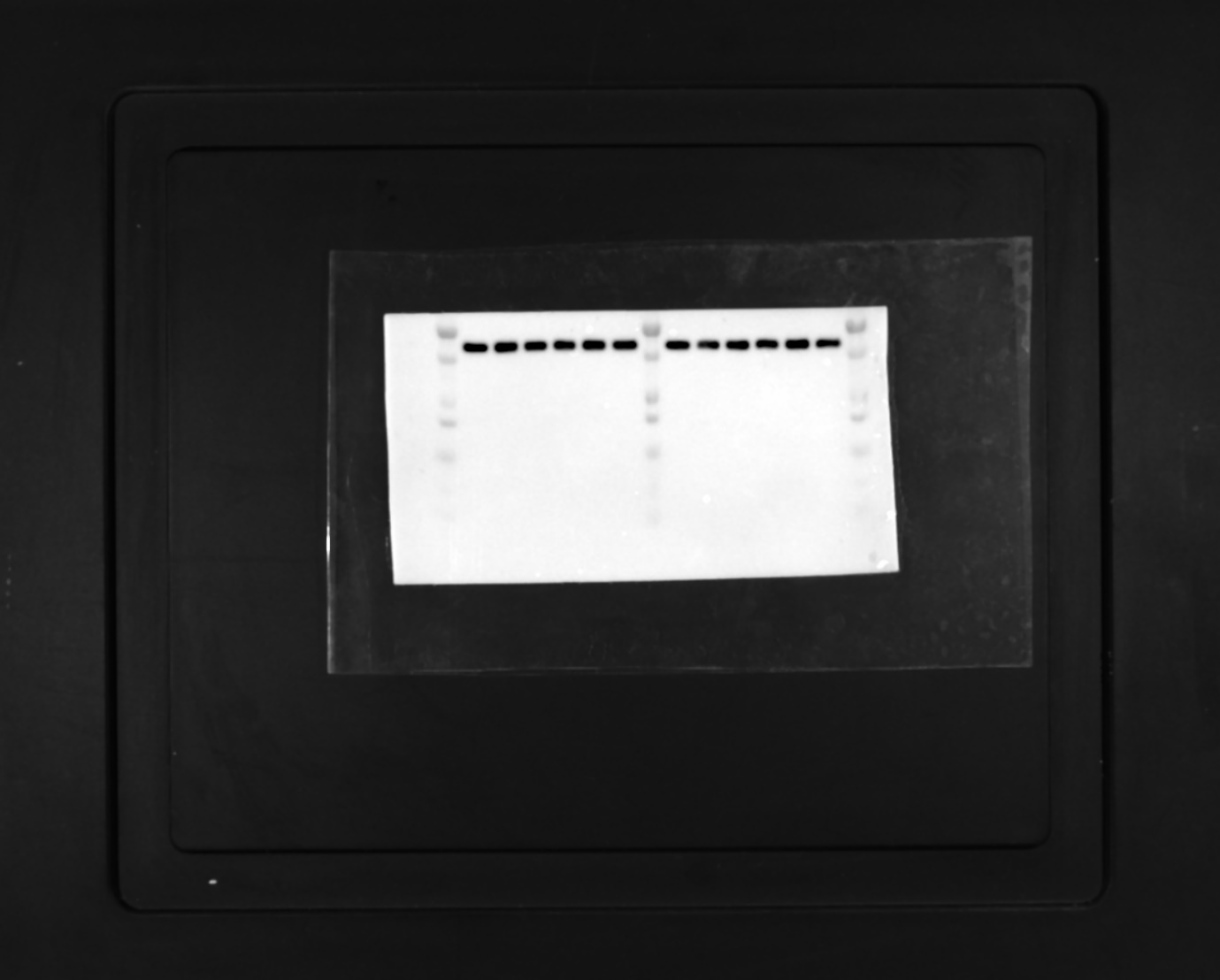


37Kda

50Kda

cGAS (67Kda)

Pan-actin (45Kda)


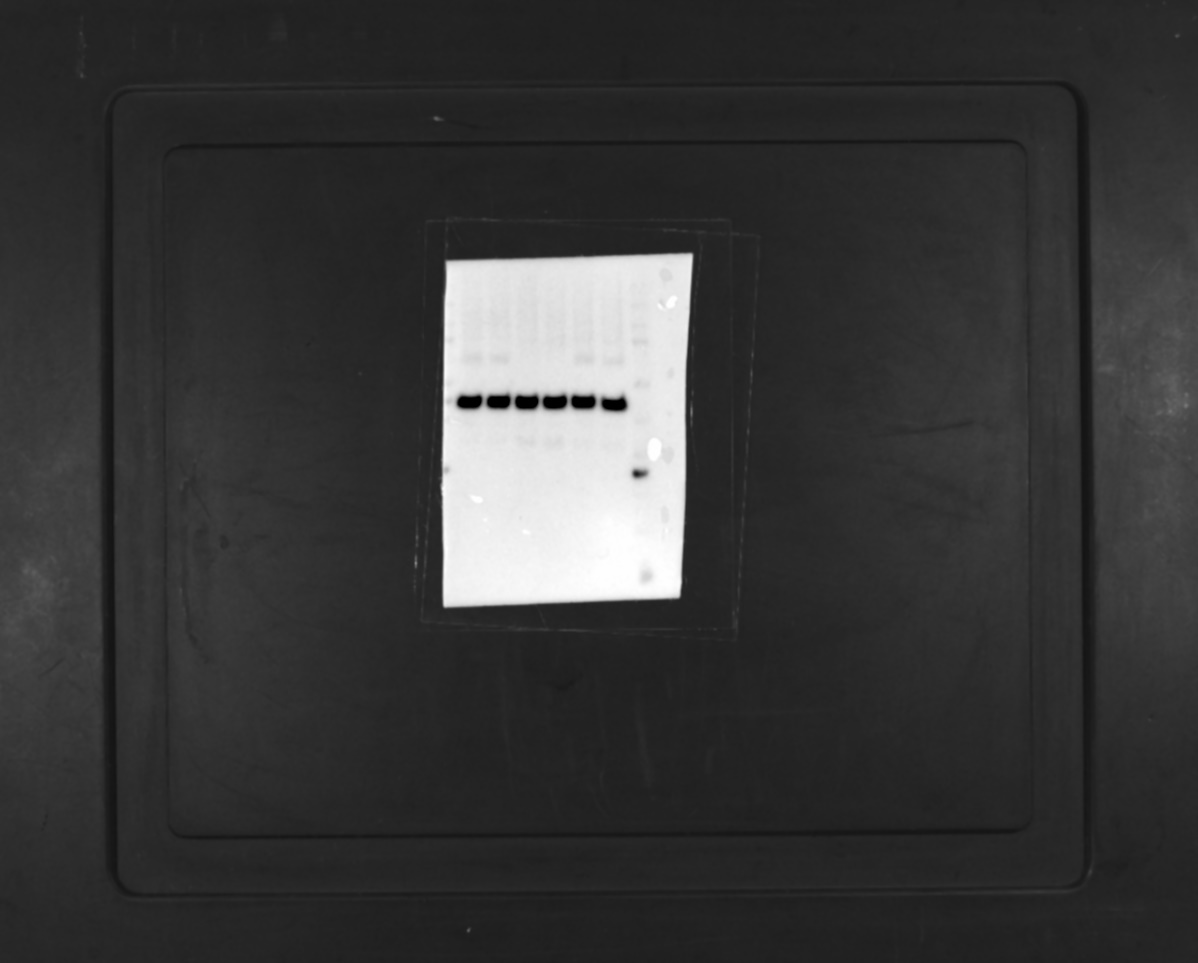


37Kda


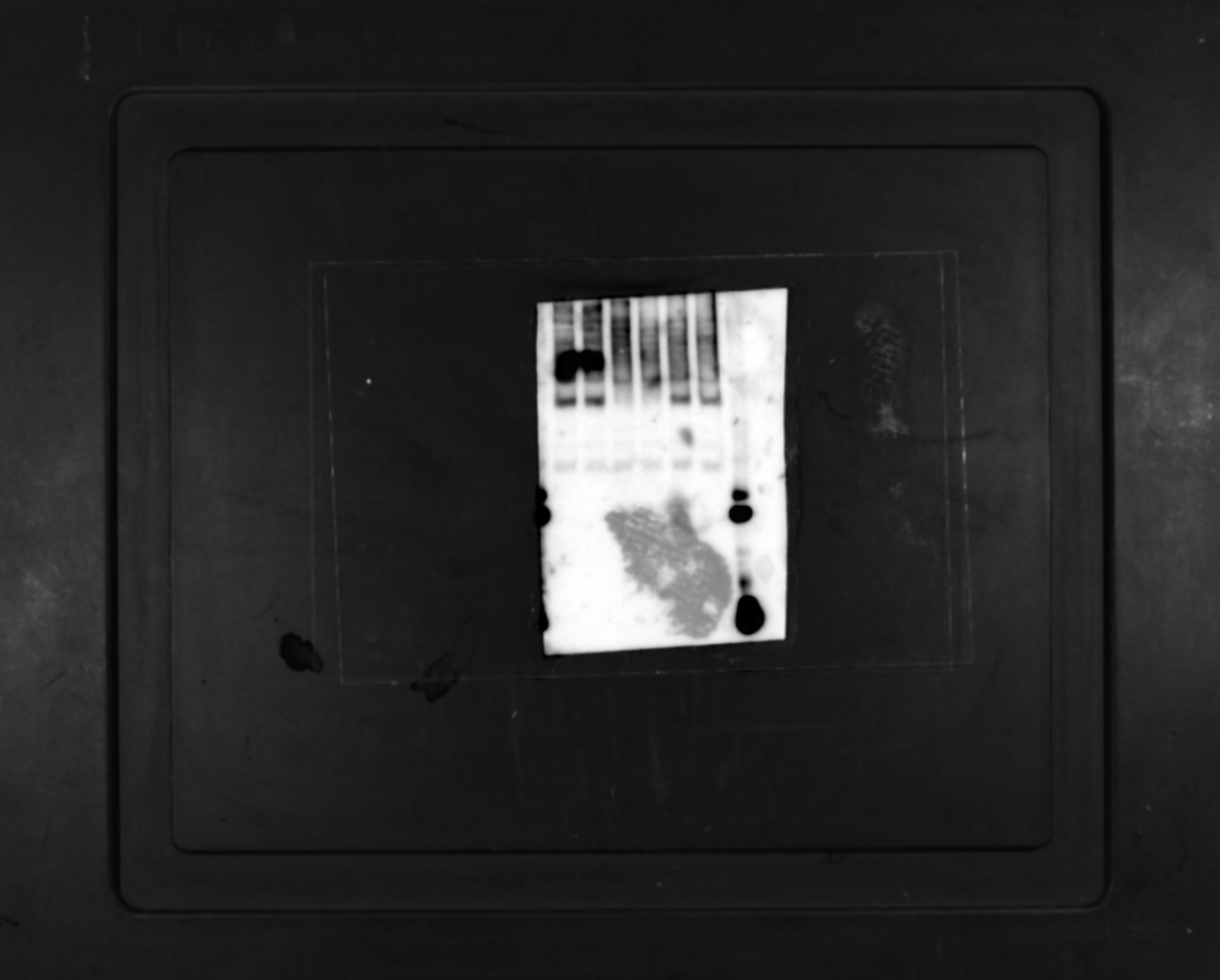

Supplement: Supplementary file 2 [file DataSheet3.docx]
